# Genome-Wide Definition of Promoter and Enhancer Usage during Neural Induction of Human Embryonic Stem Cells

**DOI:** 10.1371/journal.pone.0126590

**Published:** 2015-05-15

**Authors:** Valentina Poletti, Alessia Delli Carri, Guidantonio Malagoli Tagliazucchi, Andrea Faedo, Luca Petiti, Emilia Maria Cristina Mazza, Clelia Peano, Gianluca De Bellis, Silvio Bicciato, Annarita Miccio, Elena Cattaneo, Fulvio Mavilio

**Affiliations:** 1 Division of Genetics and Cell Biology, Scientific Institute H. San Raffaele, Milan, Italy; 2 Genethon, Evry, France; 3 Department of Biosciences, University of Milano, Milan, Italy; 4 Department of Life Sciences, University of Modena and Reggio Emilia, Modena, Italy; 5 Institute of Biomedical Technologies, National Research Council, Milan, Italy; 6 Imagine Institute, Paris, France; Pohang University of Science and Technology (POSTECH), KOREA, REPUBLIC OF

## Abstract

Genome-wide mapping of transcriptional regulatory elements is an essential tool for understanding the molecular events orchestrating self-renewal, commitment and differentiation of stem cells. We combined high-throughput identification of transcription start sites with genome-wide profiling of histones modifications to map active promoters and enhancers in embryonic stem cells (ESCs) induced to neuroepithelial-like stem cells (NESCs). Our analysis showed that most promoters are active in both cell types while approximately half of the enhancers are cell-specific and account for most of the epigenetic changes occurring during neural induction, and most likely for the modulation of the promoters to generate cell-specific gene expression programs. Interestingly, the majority of the promoters activated or up-regulated during neural induction have a “bivalent” histone modification signature in ESCs, suggesting that developmentally-regulated promoters are already poised for transcription in ESCs, which are apparently pre-committed to neuroectodermal differentiation. Overall, our study provides a collection of differentially used enhancers, promoters, transcription starts sites, protein-coding and non-coding RNAs in human ESCs and ESC-derived NESCs, and a broad, genome-wide description of promoter and enhancer usage and of gene expression programs characterizing the transition from a pluripotent to a neural-restricted cell fate.

## Introduction

Human embryonic stem cells (ESCs) are pluripotent, blastocyst-derived cells endowed with the potential to give rise to all three embryonic germ layer’s derivatives. Several protocols have been developed to obtain neural stem cells from ESCs, attempting to recapitulate in vitro the intermediate stages of neural induction, a process marked by the down-regulation of the pluripotency markers OCT4 and NANOG and up-regulation of neuroectodermal-specific markers such as NESTIN, SOX1 and PAX6. However, the regulatory circuitry driving cell fate restriction, and in particular neural commitment, is still ill-defined. The identification of the transcriptional regulatory elements involved in neural commitment is challenging, due to the difficulty in obtaining a suitable neural stem cell model. A protocol for the derivation of a homogeneous population of long-term self-renewing neuroepithelial-like stem cells (NESCs) from human ESCs has been recently established [1]. NESCs retain a stable SOX2^+^/SOX1^+^ phenotype in long-term culture and a high differentiation potential towards neuronal and glial fates with synaptic integration ability [1]. NESCs are therefore considered a valuable in vitro model to study early stages of human neural commitment and differentiation [2] and the pathogenesis of human neurodegenerative diseases [3, 4].

At present, little is known about the differential usage of promoters and enhancers occurring at ESCs restriction towards a neural fate. Specific histone modifications are currently used to define chromatin regions with different regulatory functions. In particular, monomethylation of lysine 4 of histone 3 (H3K4me1) characterizes enhancer regions, whereas its trimethylation (H3K4me3) defines active promoters [5, 6]. When present at the same genomic region, the ratio between H3K4me3 and H3K4me1 indicates the tendency of the region to act as either a promoter or an enhancer [7]. On the contrary, H3K27me3 characterizes transcriptionally silent, compact chromatin structures [8, 9]. A peculiar histone modification pattern, consisting of large regions of H3K27me3 harboring smaller regions of H3K4me3, was first described in mouse ESCs [10]. These “bivalent” domains overlay developmentally regulated genes and are thought to maintain low levels of transcription, “poised” for activation during cell commitment and differentiation, when they become selectively marked by either H3K27me3 or H3K4me3 [10].

In addition to epigenetic marks, transcription by RNA polymerase-II (Pol-II) from active enhancers was recently described, first in mouse cortical neurons [11, 12], and then in other murine and human cells [13, 14]. Enhancer RNAs (eRNAs) form a novel class of regulatory RNAs that highlight a previously unsuspected feature of active enhancers, and add complexity to the mechanisms regulating gene expression [13, 14].

The epigenetic changes occurring during human ESC induction to neuroectodermal spheres (NECs), a heterogeneous culture system of neural stem/precursor cells, were recently described [15]. Enhancers were defined as genomic regions bearing H3K4me1, H3K27me3 and H3K27ac histone modifications and binding the chromatin remodeling factors p300 and BRG1. About two hundreds putative neural commitment enhancers were defined as poised in ESCs (H3K4me1^+^/H3K27me3^+^) and active in NECs, where they lose H3K27 methylation in favor of acetylation [15]. In this study, we aimed at mapping transcriptionally active promoters and enhancers in the homogeneous and well-defined NESC cell model, by combining direct high-throughput identification of capped Pol-II RNAs defined by Cap Analysis of Gene Expression (CAGE-seq) [16, 17] with genome-wide profiling of histones modifications determined by chromatin immunoprecipitation (ChIP-seq). We inferred cell-specific, genome-wide networks of transcriptional regulation by integrating CAGE-seq, ChIP-seq and gene expression microarray data, in an attempt to better define the molecular circuitry associated with the transition from pluripotent to neural restricted stem cells.

## Materials and Methods

### Cell cultures

The human Embryonic Stem Cell line H9 (NIH code WA09, ISL1 Ds-Red) was previously published [18], and was kindly provided by the group of K.R. Chien. ESCs were maintained on irradiated mouse embryonic fibroblasts (MEFs) (S1 Materials and Methods). Neural differentiation was induced according to the procedure described in [1]. Briefly, 4-days embryoid bodies were transferred to polyornithine-coated dishes and propagated in N2-supplemented DMEM/F12 (Invitrogen). Within 10 days, small rosettes were mechanically isolated and propagated for 1 to 7 days as free-floating neurospheres, dissociated into single cells in trypsin/EDTA and re-plated on polyornithine/laminin pre-coated plastic dishes in FGF2/EGF-containing medium. Expression of SOX2 and Nestin on NESCs, and TUBB3, MAP2 and GAD65-67 on terminal neurons, was detected by immunofluorescence assay as previously described [19]. Images were acquired with a Leica DMI 6000B microscope (10× and 20× objectives), and analyzed with LAS-AF imaging software [20]. The following antibodies were used: anti-SOX2 (MAB4343, Millipore), anti-Nestin, (MAB1259, R&D Systems), anti-beta-3 tubulin (G7121, Promega), anti-GAD-65 and -67 (AB1511, Millipore), anti MAP2 (556320, BD Pharmingen) and secondary antibodies conjugated to Alexa fluorophores 488 or 568 (Molecular Probes, Invitrogen). Nuclei were stained with Hoechst 33258 (Molecular Probes, Invitrogen).

### Microarray gene expression profiling

Total RNA was extracted from 1–2 10^6^ cells from triplicate cultures of ESCs and NESCs, transcribed into biotinylated cRNA and hybridized onto GeneChip HG-U133 Plus 2.0 Arrays (Affymetrix) according to the manufacturer protocol (Affymetrix, Santa Clara, CA). Signals were converted to expression values by the RMA algorithm [21] and HG-U133 Plus 2.0 custom Chip Definition Files (CDF) based on GeneAnnot [22]. Differentially expressed genes were identified by Significance Analysis of Microarray method (SAM, [23]) setting the q-value [24] threshold equal to 0 (or ≤0.01) and considering fold change (FC) levels increasing from 2 to 10.

### Gene functional annotation, gene pathways and transcription factor binding site analysis

Gene Ontology (GO) functional annotation of differentially expressed genes was performed by DAVID v6.7. GO-term enrichments were based on a modified one-tail Fisher Exact p-value (EASE score) after Benjamini correction for multiple testing, and considered significant at p<0.05. Networks of genes associated to cell-specific and up- and down- regulated CAGE promoters were made with the Ingenuity Pathway Analysis (IPA) software, with the tool “my pathways” based on the Ingenuity Knowledge Base dataset. For a complete IPA legend refer to IPA website (www.ingenuity.com).

The search for transcription factor binding site (TFBS) enrichment in cell-specific promoters, enhancers and CAGE-enhancers was performed by the HOMER tool [25] (http://homer.salk.edu/homer/index.html). In CAGE-enhancers, the search for TFBSs was made on a region of 400bp centered on the middle-point of CAGE-enhancer region.

### CAGE-seq promoter profiling

CAGE-seq was performed by DNAFORM Inc. at RIKEN Omics Science Center on an Illumina Genome Analyzer as previously described [16] (see S1 Materials and Methods). For CAGE tags mapping to multiple genome locations, a weighting strategy based on the number of CAGE tags within a 200-bp interval around each candidate mapping location was applied [26]. Equal weights were used if no unique tags were found within the 200-bp region for all candidate mapping locations. Level-1 promoters (Transcription Start Sites, TSSs) were created by summing the weighted number of CAGE tags at each genome position and clustered into Level-2 promoters (CAGE promoters) if closer than 20 bp on the same chromosomal strand, and if resulting an expression level of at least 10 tags per million (tpm) in at least one experimental condition. The tpm values were calculated for each Level-1 and Level-2 promoter dividing the number of CAGE tags of each promoter in each experimental condition by the total number of mapped CAGE tags in that condition, and multiplied by 10^6^. For the promoter annotation, RefSeq and Gencode (release 14) genes and transcripts coordinates were downloaded from the UCSC table browser for the hg19 genome assembly. The significance of differential promoter expression was determined by a χ^2^ test.

### Chip-seq library preparation and sequencing

Chromatin was prepared from pellets of 5 10^6^ NESCs following the truChIP Chromatin Sheering Kit standard protocol (Covaris Inc.) and sonicated to obtain DNA fragments averaging 200 bp. 10 ng of DNA immunoprecipitated with anti-H3K4me1 and anti-H3K4me3 antibodies (Ab8895 and Ab8580, Abcam Plc.) or control input DNA were used to prepare the sequencing libraries, which were checked by capillary electrophoresis and sequenced in one lane of a single-strand 50 bp GAIIx Illumina Run (S1 Materials and Methods). Raw reads were mapped against the human reference genome (hg19 assembly) using Bowtie [27] allowing up to 2 mismatches. The results of multiple mapping step were pooled together into a single BAM file that was then processed by using SAMtools [28] and converted into a bed file using BEDTools [29] (data are available at GEO with the accession number GSE61267). The quality of each sequenced sample was then checked using cross-correlation analysis implemented in spp R package [30]. ChIP-seq peak calling was performed using SICER default parameters [31] and using each INPUT data (control DNA) to model the background noise. ChIP-Seq data on H9 ESCs (GSM667626, GSM605316, GSM667622) were downloaded from the NIH Roadmap Epigenomics Mapping Consortium web site.

### Definition of promoters and enhancers from ChIP-seq data

A custom R-workflow was developed to identify promoters and enhancers on the basis of H3K4me1 and H3K4me3 enrichment at particular genomic regions. The procedure starts from ChIP-seq peaks generated by SICER and identifies, using BEDtools [29], the genomic regions where H3K4me1 and H3K4me3 peaks were present. Then, regions bearing H3K4me3 were considered promoters and regions bearing H3K4me1 peaks were considered enhancers. When H3K4me3 and H3K4me1 peaks overlapped, the regions were classified either promoters or enhancers if the log ratios of H3K4me3 and H3K4me1 normalized tag count was, respectively, greater or lower than 0. The intensity of H3K4me3 around the CAGE TSS, and the intensity of H3K4me1 inside the enhancers were calculated by ngs plot [32]. The statistical significance of the differential H3K4me1 or H3K4me3 intensity was calculated by Wilcoxon test, implemented in the R package “wilcox.test”, correcting the resulting p values by Bonferroni method.

CAGE-enhancers were predicted as described in [33], pooling together the samples and excluding regions of 1kb around TSSs and exons +/-200bp. Differential expression of CAGE-enhancers was assessed by the R package EdgeR [34], setting a dispersion value of 0.3^2^.

## Results

### *In vitro* derivation of human neuroepithelial stem cells from ESCs

**Fig 1:**
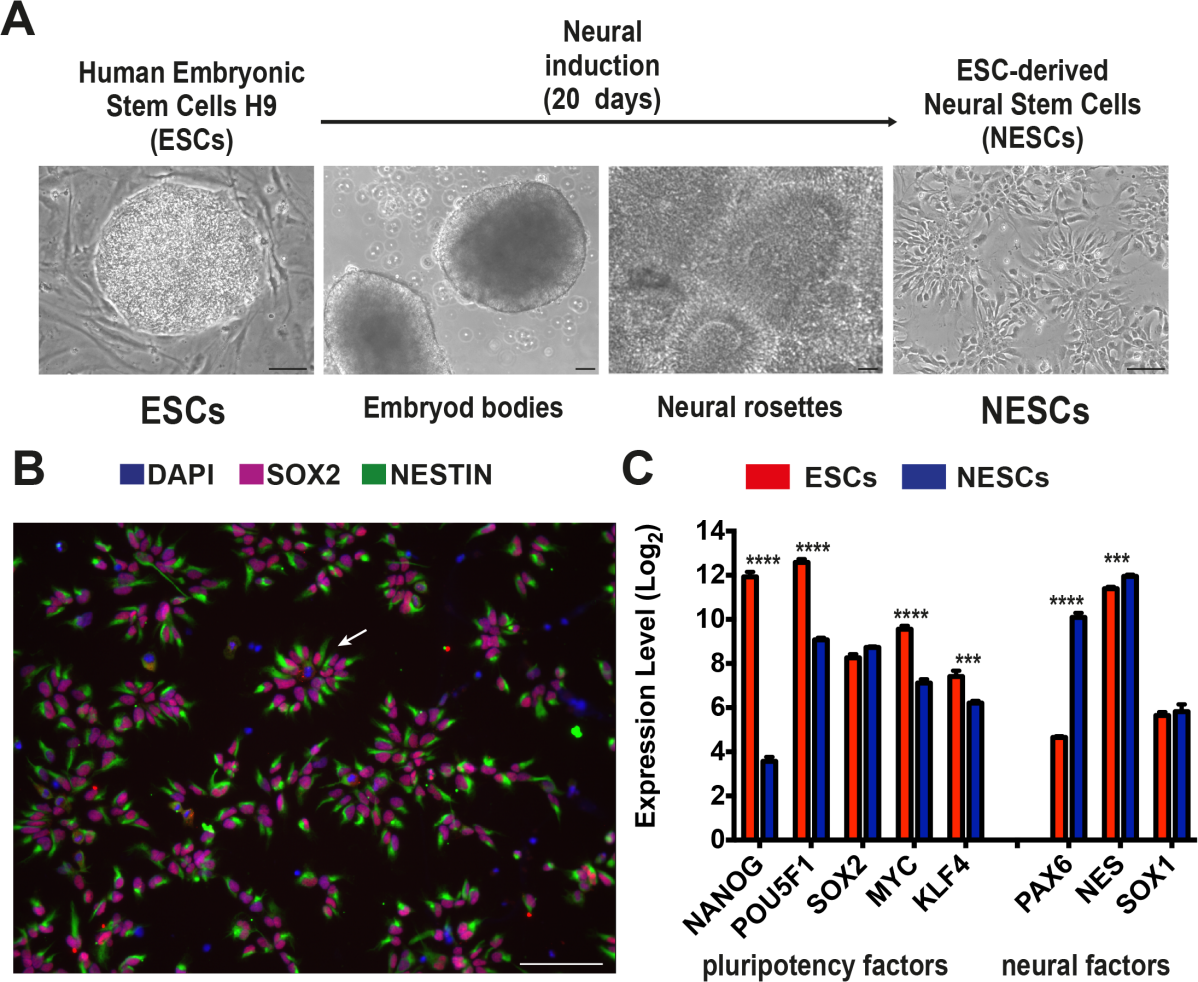
Neural induction of human ESCs to NESCs. A) Schematic representation of the protocol used to differentiate the ESC line H9 to NESCs, entailing the formation of embryoid bodies, followed by neural rosette formation and mechanical isolation. Bar scale 100 μm. B) NESCs cultured in EGF and FGF2 containing medium staining positive for SOX2 in the nucleus (red) and NESTIN in the cytoplasm (green), bar scale 100 μm. NESCs showed a tendency to distribute in rosettes-like structures (white arrow), with the nuclei located at the center and the cytoplasms at the periphery. C) Expression level of pluripotency and neural factors in the three independent cultures of ESCs and NESCs as obtained from the microarray analysis. Genes marked by asterisks were significantly differentially expressed (p ≤0.001***, p ≤0.0001 ****, unpaired t test with Bonferroni correction).

ESCs were differentiated into NESCs as previously described [1]. Briefly, 4-day-old embryoid bodies were generated from human ESC line H9. Neural tube-like structures developed in the embryoid body outgrowth within 10 days, followed by the appearance of small rosette-shaped cell clusters that were mechanically isolated and further propagated as neurospheres for one week. Spheres were disaggregated into single cells and plated to establish stable adherent NESC cultures (Fig 1A). NESCs stained positive for the neural stem cells markers NES, predominantly expressed in stem cells of the central nervous system [35], in combination with SOX2, a pluripotency transcription factor essential for neural stem cell proliferation and maintenance [36] (Fig 1B). After 20 days of culture in the absence of growth factors NESCs spontaneously differentiated into GAD65/67^+^ GABAergic neurons (S1 Fig).

### Neural induction of ESCs is associated to major changes in the gene expression program

**Fig 2:**
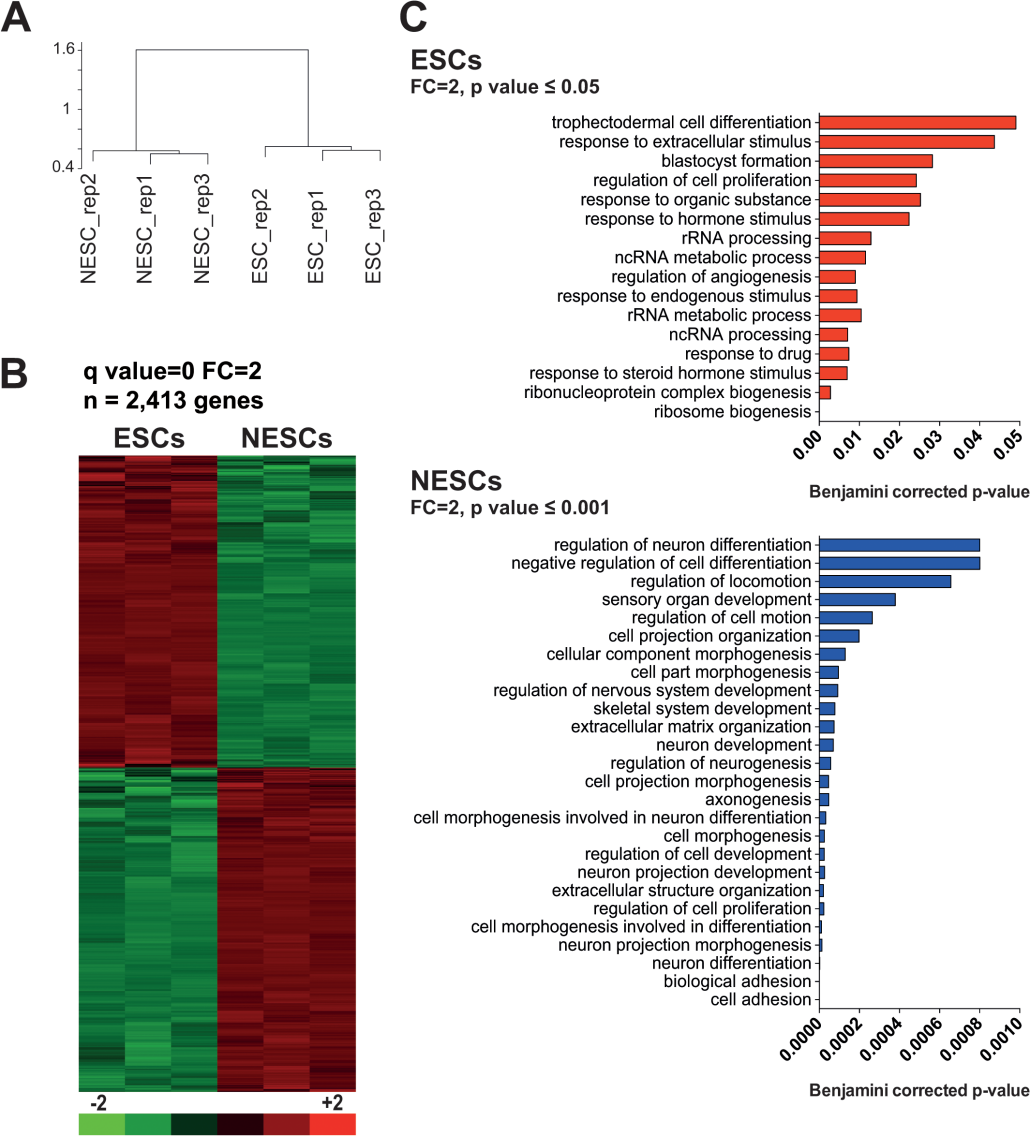
Change in gene expression profile during ESC neural induction. A) Global unsupervised clustering performed on the entire pool of 19,204 genes indicates that, at the transcriptional level, ESCs and NESCs are two distinct cell populations. Red boxes highlight reproducible clusters that are strongly supported by data (Bootstrap Probability value ≥95%). B) Heat map of the subset of 2,413 genes that change their expression levels in ESCs as compared to NESCs (at False Discovery Rate <0.01 and absolute FC level ≥2). Expression levels are presented as row-wise standardized values (log_2_ fold change). C) Functional enrichment of the 2,413 differentially expressed genes obtained using DAVID GO annotation. Upper plot reports the GO categories associated to genes up-regulated in ESCs, while in the lower plots are the functional enrichment of genes over-expressed during neural induction, i.e. in NESCs.

We analyzed gene expression changes during neural induction of ESCs by hybridizing total RNA extracted from triplicate ESC and NESC cultures to Affymetrix HG-U133 plus 2.0 Gene Chip arrays (GSE61267). Gene expression data highlighted that pluripotency master regulators and reprogramming factors, such as NANOG, POU5F1 (OCT4), MYC and KLF4 [37, 38], were significantly down-regulated (t test, Bonferroni corrected p ≤0.001) in NESCs (Fig 1C). SOX2, encoding a transcription factor essential for pluripotency of ESCs and maintenance of NESCs, presented similar expression levels in both cell types (Fig 1C). Conversely, the neural markers PAX6 and NES were significantly up-regulated in NESCs (t test with Bonferroni correction, p ≤0.001) (Fig 1C). Genome-wide unsupervised analysis, performed on the entire pool of 19,204 genes, indicated that ESCs and NESCs were two distinct cell populations at the transcriptional level (Fig 2A). This was further confirmed by supervised analysis with Significance Analysis of Microarray (SAM), which returned 2,413 genes differentially expressed in ESCs as compared to NESCs (at False Discovery Rate <0.01 and absolute fold change (FC) level ≥2; Fig 2B). Transcriptional differences among ESCs and NESCs remained remarkable also when raising the fold change threshold from 2 to 4 or 10. In fact, 375 and 371 genes were, respectively, up- and down-regulated during neural induction at FC ≥4 (data not shown), and 112 and 124 at FC ≥10 (S1 Table). Genes up-regulated in NESCs encoded transcription factors involved in neural system development, such as PAX6, POU3F2, MEIS1, RFX4 [37, 39, 40], cell proliferation and suppression of terminal differentiation, such as TFAP2B (AP2beta) [41, 42], and an adhesive junction-associated protein of the armadillo/beta-catenin superfamily involved in brain development and several neurological disorders, i.e., CTNND2 [43, 44]. On the other hand, other genes belonging to the ESC Core Regulatory Network, or coding for pluripotency key factors, were down-regulated in NESCs (e.g., NANOG, POU5F1 and LEFTY1) [37, 45].

Analyzing this list of 2,413 differentially expressed genes by Gene Ontology functional enrichment, we found that the subset of up-regulated genes in ESCs was enriched in several GO terms accounting for functional categories of early embryonic development, such as blastocyst formation, and RNA processing and ribosome biogenesis, comprising many MYC direct target genes [46] (Fig 2C). Conversely, regulation of morphogenesis, cell development and proliferation, and in particular neurogenesis, axonogenesis and neuron development and differentiation, were significantly up-regulated during transition from ESCs to NESCs (Fig 2C).

### Differential promoter usage in ESCs and NESCs

**Fig 3:**
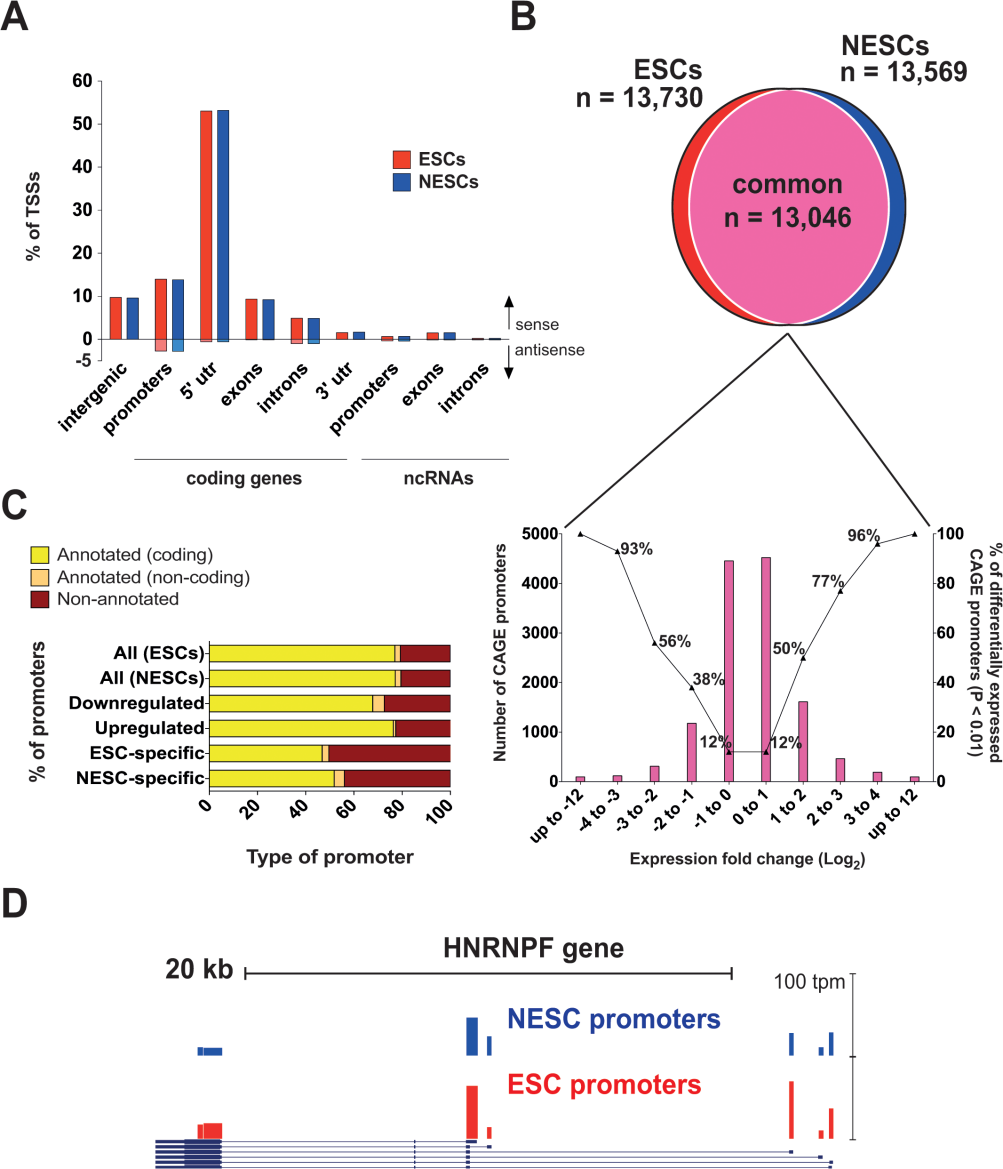
Differential Pol-II promoter usage during differentiation of ESCs to NESCs. A) CAGE mapping of transcription start sites (TSSs) to intergenic regions or to promoters (500 bp around the RefSeq TSS), 5’ UTRs, exons, introns and 3’ UTRs of protein-coding and non-coding RefSeq transcripts annotated on the hg19 assembly of the human genome. Bars indicate the percentage of each category of CAGE-defined TSSs in the sense (above the X axis) or anti-sense (below the X axis) transcriptional orientation with respect to the annotated transcript. B) Venn diagram showing the fraction of common and cell-specific CAGE promoters mapped in ESCs and NESCs. For the common promoters, a histogram indicates the number of promoters (left Y axis) distributed in different categories of differential expression during ESC neural induction, with FC from -12 to 0 (down-regulated promoters) and from 0 to +12 (up-regulated promoters). For each category, the fraction of promoters differentially expressed at a statistically significant level (χ^2^ test, p ≤0.01) is indicated (right Y axis). C) Proportion of all, cell-specific, upregulated and down-regulated CAGE promoters annotated to RefSeq protein-coding (yellow) or non-coding (light brown) genes. The fraction of unannotated promoters is shown in dark brown, and significantly increases in the regulated and cell-specific categories. D) Example of a gene (HNRNPF) associated to multiple alternative promoters in both cell types. All 5 known HNRNPF promoters were mapped in ESCs (red bars) and NESCs (blue bars) by CAGE-seq, which identified also two novel promoters in the last exon of the gene. The height of the bars reflects the promoter strength expressed in tpm (scale on the right).

To define changes in promoter usage in a qualitatively and quantitatively fashion during neural induction, we performed CAGE-seq on total RNA extracted from ESCs and NESCs. CAGE-seq yielded 14.5 and 16.8 x 10^6^ raw CAGE tags in ESCs and NESCs, respectively. Transcription Start Sites (TSSs) and CAGE promoter expression values were expressed as tags per million (tpm). TSSs were defined by summing the weighted number of CAGE tags at each genomic position, and clustered into CAGE promoters if closer than 20 bp on the same chromosomal strand and yielding at least 10 tpm (see Material and Methods and S2 Fig). Within each CAGE promoter, the TSS with the highest expression value was considered the most used TSS, and mapped to the human genome. On average, ~67% of the CAGE-mapped TSSs aligned within 500 bases from annotated TSSs of RefSeq protein-coding genes (14%) or in their 5’ UTR regions (53%), while ~16% mapped within introns, exons or 3’ UTRs (Fig 3A). About 2.5% of the TSSs mapped to annotated non-coding transcripts, while ~10% to intergenic regions, likely as the result of the transcription from yet uncharacterized promoters. Interestingly, as many as 5% of the TSSs were in antisense orientation with respect to annotated transcripts and 1.2% were bidirectional, providing additional evidence for divergent transcription in mammalian promoters [47, 48]. In addition, we noted that ~6% of the TSSs in both cell types mapped to repeated elements, a proportion similar to that observed in mouse brain tissues [49], with a predominance of microsatellites, low-complexity elements, SINEs, LINEs and LTR families of repeats (not shown).

Overall, we identified a total of 14,253 CAGE promoters mapped in at least one cell type (GSE61267), of which 13,730 were found expressed in ESCs (tpm range: 1–14,535) and 13,569 in NESCs (tpm range: 1–25,794). Among the 14,253 CAGE promoters, 684 were expressed exclusively in ESCs and 523 in NESCs (Fig 3B), which decreased to 252 and 189 respectively after statistical correction for false discovery (χ2 test, p ≤0.01), and were defined as ESC- and NESC-specific. Over 91% of the CAGE promoters were active in both cells types, the majority of which (62.5%) were expressed at virtually the same level. The remaining 37.5% of common CAGE promoters were differentially expressed in the two cell types with a fold change (expressed as log_2_) ranging between 1 and 12 (Fig 3B). As expected, the number of CAGE promoters expressed at significantly different levels (χ2 test, p ≤0.01) in either cell type increased with the fold change, from 38% and 50% at a fold change <1 up to 100% at a fold change >4 (Fig 3B).

We assigned each CAGE promoter to the closest RefSeq gene within an arbitrarily defined distance of ≤400 bp between the gene TSS and either end of the promoter on the same strand. Overall, 80% of all CAGE promoters were annotated to RefSeq genes in both cell types. Interestingly, the fraction of un-annotated promoters increased up to 27% and 23% in differentially expressed (i.e., up- and down-regulated CAGE promoters, fold-change ≥3, p ≤0.01) and 50% and 44% in ESC- and NESC-specific promoters respectively (Fig 3C). Notably, one of NESC-specific un-annotated CAGE promoters was located 694 bp upstream of the neural marker gene SOX1, while transcription by its RefSeq-annotated TSS was not detected in ESCs or in NESCs.

Overall, ~97% of annotated CAGE promoters were associated to protein-coding genes, while the remaining 3% to non-coding RNAs (S2 Table), i.e., antisense RNAs, lncRNAs, miRNAs and pseudogenes. Interestingly, up to 8% of the NESC-specific and 6% of the ESC-specific promoters were associated to non-coding transcripts. In some cases, we were able to discriminate the promoters engaged exclusively in miRNA transcription from those of host genes, since mapping on opposite strands (i.e., MIR198, located in the 3’ UTR of the FLST1 gene) or into intergenic regions (MIR302B, MIR302C and MIR527).

The number of protein-coding genes associated to CAGE promoters was 9,502 and 9,372 in ESCs and NESCs respectively, where ~11% of the genes used two or more alternative promoters. In particular, we found 4 genes (HNRNPF, SEPT9, YWHAZ, PLEC) transcribed from five alternative promoters in both cell types (Fig 3D). The correlation between the gene expression programs analyzed by CAGE-seq and Affymetrix arrays was investigated by quantitative Pearson correlation, and yielded a coefficient of 0.53 in ESCs and 0.50 in NESCs (S3A and S3B Fig). The correlation increased to 0.65 and 0.60 respectively when calculated on the set of genes associated to differentially expressed promoters (S3C and S3D Fig).

### Distinct classes of active promoters distinguish ESCs from NESCs

As expected, we found several coding and non-coding members of the Pluripotency Core Regulatory Network among genes transcribed from ESC-specific and down-regulated promoters (S4 and S5 Figs and S3 Table), such as the master regulators NANOG and OCT4 (POU5F1), and the miRNAs MIR302B and MIR302C, reported to play a key role in pluripotency and cell reprogramming [50–52]. Moreover, ESC-specific promoters were associated to several other genes with relevant regulatory roles in undifferentiated ESCs. Among them, LEFTY1, an inhibitor of Nodal signaling necessary for ESCs pluripotency maintenance [53], TDGF1 (CRIPTO1) a key regulator of embryonic development and marker of undifferentiated ESCs [54], the transcription factors SALL3 and OTX2, the recently proposed cell-reprogramming factor UTF1 [55], and the chromatin-remodeling factor PMDR14, involved in activating pluripotency-associated genes and suppressing differentiation-associated genes in human ESCs [56].

**Fig 4:**
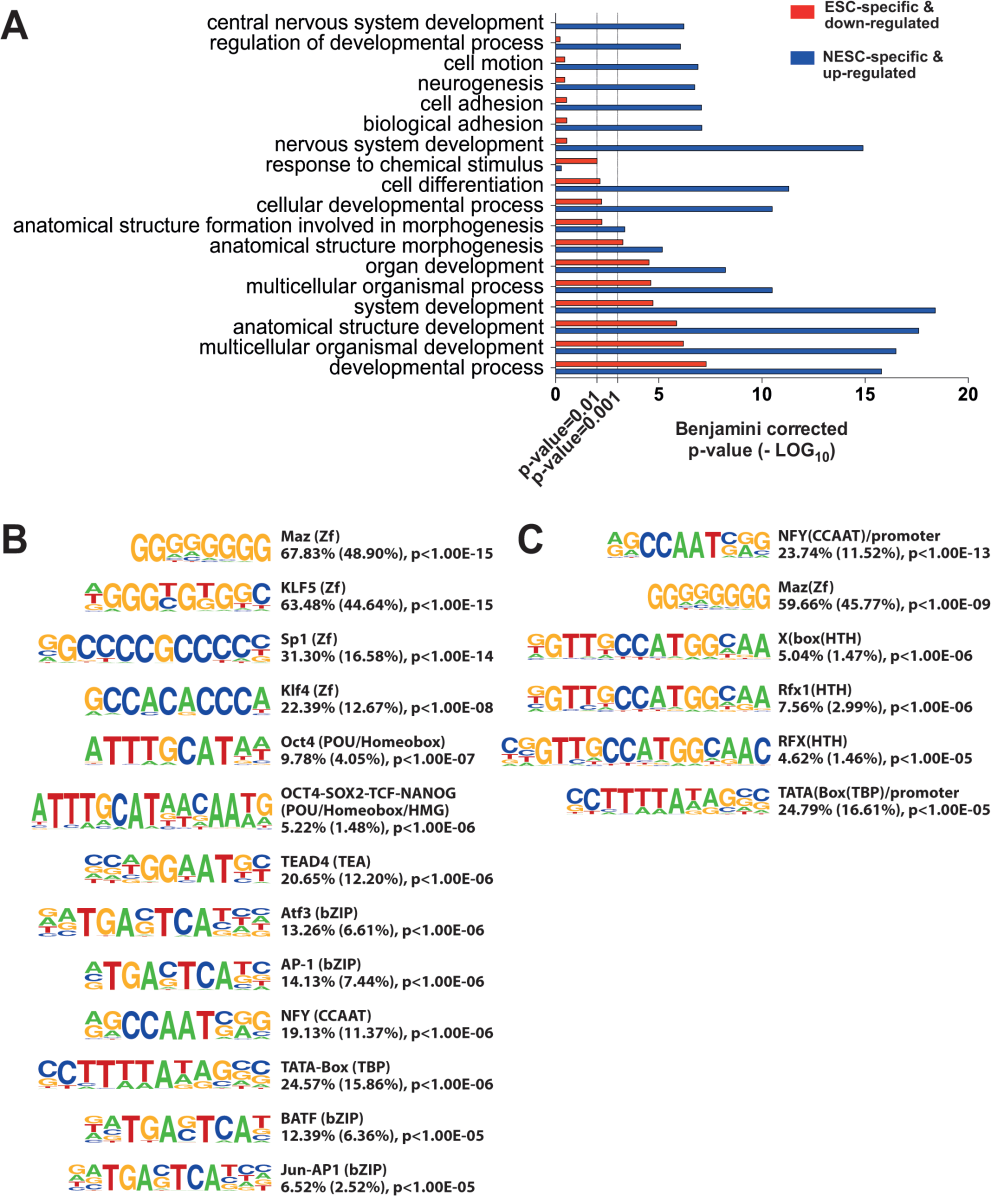
Cell-specific CAGE promoters. A) GO categories associated to genes transcribed by ESC-specific and down-regulated promoters (red bars), and by NESCs-specific and up-regulated promoters (blue bars). The X-axis indicates the level of statistical significance of the association to each category, expressed as-log_10_ of the p-value after Benjamini correction for false discovery rate. B and C) HOMER analysis of putative TFBS within ESC-specific and down-regulated promoters (B) and NESC-specific and up-regulated promoters (C). For each TFBS motif, we indicated the p value, and the percentage of promoters and background sequences (between brackets) containing the putative TFBS. ESC-specific and down-regulated promoters showed enrichment for binding motifs of the pluripotency TFs, while NESC-specific and up-regulated promoters were enriched for ubiquitous TFs (NFY, MAZ and TBP) and the RFX-family HTH factors.

**Fig 5:**
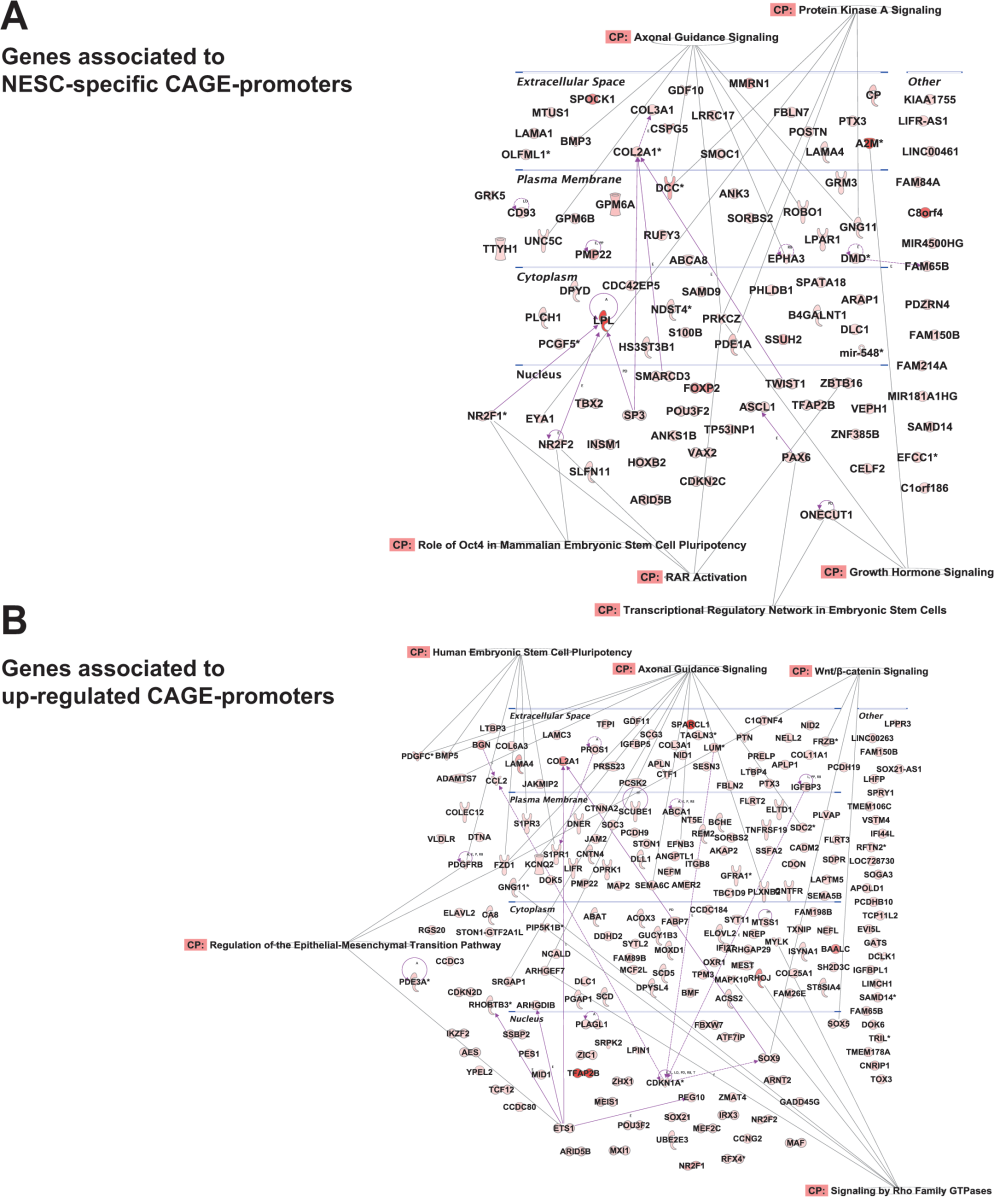
Networks of genes associated to NESC-specific and up-regulated CAGE promoters. The networks visually represent the connections between the genes associated to NESC-specific (up) and up-regulated CAGE promoters (down). Most of the genes are included in the regulatory pathways of axonal guidance signaling, ESC pluripotency and signal transduction. Purple arrows indicate the connections between genes based on the Ingenuity Knowledge Base dataset (dotted or solid lines for indirect and direct relationships respectively). Then, genes included in IPA canonical pathways (CP) are indicated by grey arrows. The shape of the gene symbol indicates the corresponding protein function, while the color (from white to red) represents the CAGE expression level of the promoter associated to the gene (for NESC-specific promoters) or its ratio between ESCs and NESCs (for upregulated CAGE promoters). For a complete IPA legend see http://ingenuity.force.com/ipa/articles/Feature_Description/Legend

Likewise, many genes transcribed by NESC-specific and up-regulated promoters were among the members of the Network, repressed in pluripotent cells. Interestingly, GO categories associated to genes transcribed by ESC-specific or down-regulated promoters were related to generic embryonic developmental processes (anatomical structure development and morphogenesis) (Fig 4A), while genes associated to NESC-specific and up-regulated promoters included the early neuroectodermal regulator PAX6 [57] and several transcription factors already described to be involved in stem cell biology and differentiation, such as ETS1, and a large fraction of poorly characterized genes, specifically associated to regulation of nervous system development, neurogenesis, cell motion, adhesion and differentiation (Figs 4A and 5, and S3 Table). These differences reflect global changes in gene expression programs accompanying loss of pluripotency and acquisition of a committed phenotype.

To understand the regulatory circuitry operating on differentially regulated promoters, we analyzed putative transcription factor binding sites (TFBSs) within a region extending from -300 to +100 bp from the most expressed TSS in cell-specific and up- and down-regulated CAGE promotes by the HOMER tool [25]. Significantly enriched (p <10^–5^) TFBS motifs are shown in Fig 4. ESC-specific and down-regulated promoters showed enrichment for binding motifs of the pluripotency TFs OCT4, SOX2, NANOG and KLF4 (Fig 4B), while NESC-specific and up-regulated promoters were essentially enriched for the ubiquitous NFY, MAZ and TBP factors and the RFX-family HTH factors (Fig 4C).

### Epigenetic Profiling of ESC and NESC CAGE promoters

**Fig 6:**
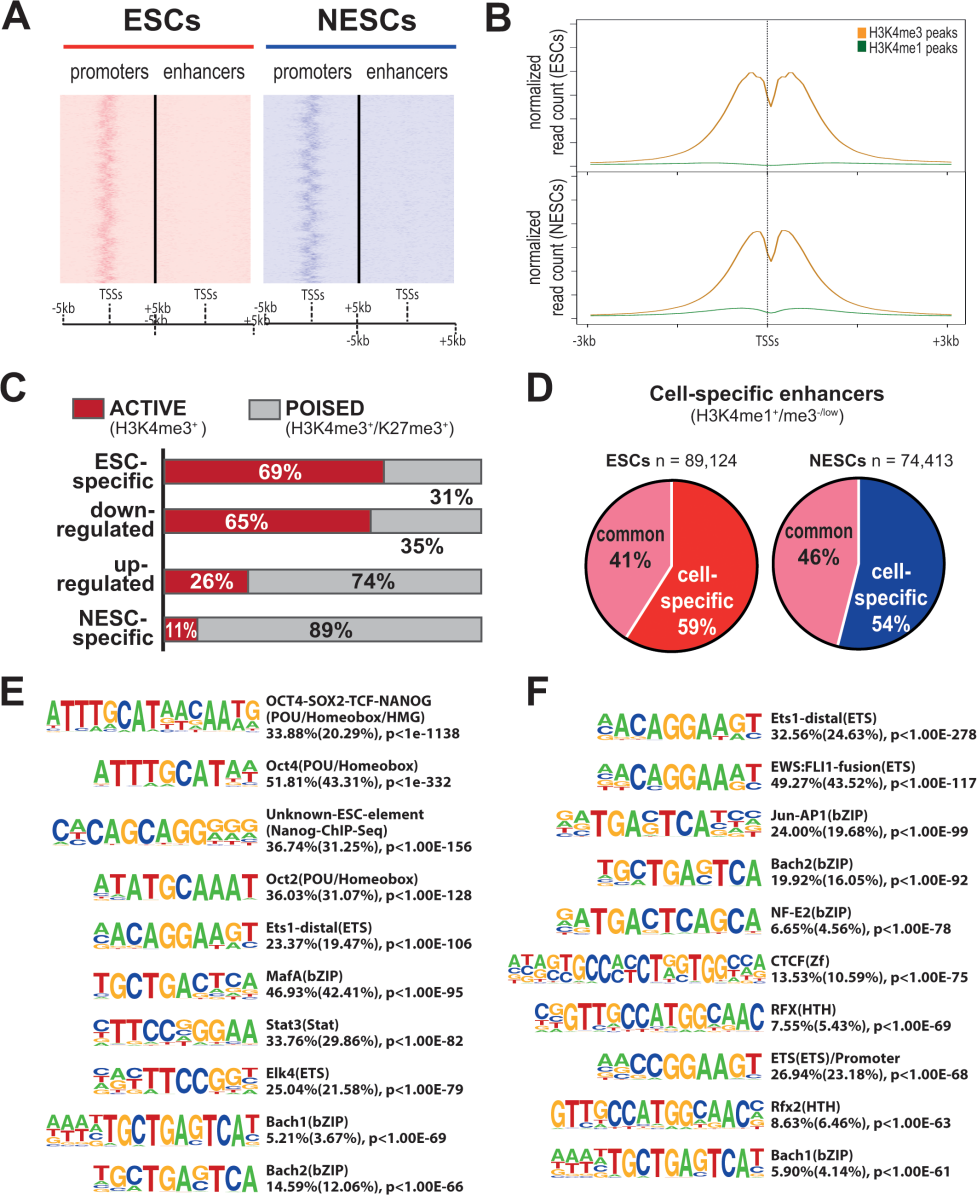
Genome-wide mapping of epigenetically-defined promoters and enhancers in ESCs and NESCs. A) Heat maps showing the distribution of promoter (left) and enhancer (right) regions in a window of ±5-kb from CAGE-mapped TSSs in ESCs and NESCs. Promoter islands are defined as H3K4me3^+/high^/me1^-/low^, enhancers as H3K4me1^+/high^/me3^-/low^. Promoter islands are clustered around CAGE-mapped TSSs, whereas enhancers are spread out. B) Average profile of single H3K4me3 (orange) and H3K4me1 (green) peaks around the TSS, in a ±3-kb. Normalized read count means histone modification read count per million mapped reads. C) Epigenetic state of CAGE promoters in ESCs. The histogram shows the fraction of ESC-specific, down-regulated, up-regulated and NESC-specific promoters with the epigenetic signature of active (H3K4me3^+^, red) or poised (H3K4me3^+^/H3K27me3^+^, grey) promoter. The most part of up-regulated and NESC-specific promoters are poised for transcription in ESCs. D) Pie-diagrams showing the fraction of common and cell-specific total enhancers mapped in ESCs and NESCs. E) Analysis of putative TFBS enrichment within cell-specific enhancers as determined by HOMER tool. ESC-specific enhancers showed enrichment of binding motifs for the pluripotency TFs, as observed for cell-specific promoters, and for ETS family factors. NESC-specific enhancers were enriched for ETS-family, RFX-family and Jun/AP1 factors.

We then analyzed the epigenetic profile associated with differentially regulated promoters by genome-wide mapping of H3K4 mono- and tri-methylations in NESCs by ChIP-seq (GSE61267). Genome-wide maps of H3K4me3, H3K4me1 and H3K27me3 for ESCs H9 cells, available from the NIH Roadmap Epigenomics Mapping Consortium, were downloaded and re-analyzed. We identified 13,640 and 13,098 genomic regions in ESCs and NESCs respectively, carrying the H3K4me3^+^/me1^-^ or H3K4me3^high^/me1^low^ epigenetic marks of promoters. As expected, these regions co-mapped with the majority of the CAGE promoters (Fig 6A). In particular, 61% and 60% of H3K4me3^+^/me1^-^ or H3K4me3^high^/me1^low^ islands in ESCs and NESCs respectively overlapped 79% and 76% of CAGE promoters in a window of ±2 kb. The H3K4me3 peaks showed the expected bimodal, bell-shaped distribution around the TSSs (Fig 6B), and the intensity of H3K4me3 significantly correlated with CAGE promoter expression level (S6A Fig).

Interestingly, most of the CAGE promoters associated to protein-coding genes (88% in ESCs and 83% in NESCs) showed a promoter epigenetic signature, compared to only about 43% of the un-annotated CAGE promoters (43% in ESCs and 44% in NESCs) (S4 Table). Most of the H3K4me3^+^/me1^-^ or H3K4me3^high^/me1^low^ islands (69%) overlapped in the two cell types, as observed for the CAGE promoters, and therefore did not change during neural commitment. In ESCs, 21% (2,206) of the CAGE promoters associated with the H3K4me3^+^/me1^-^ or H3K4me3^high^/me1^low^ modifications was marked also by H3K27me3, an indication of a poised transcriptional status. As expected, the average expression level of the “bivalent” promoters was significantly lower (p ≤0.0001 by t test) than the overall promoter population (S7A Fig). These promoters included 183 (38%) of the 476 NESC-specific or NESC up-regulated promoters (S5 Table), defining a group of promoters that transit from a poised to a transcriptionally active state during neural commitment of ESCs. Among the differentially expressed H3K4me3^+^/me1^-^ or H3K4me3^high^/me1^low^ CAGE promoters, most ESC-specific and down-regulated promoters had an active epigenetic signature, whereas most NESC-specific and up-regulated promoters showed a poised signature in ESCs (Fig 6C).

### Identification of cell-specific enhancers and eRNAs

We identified 89,124 and 74,413 genomic regions exhibiting the H3K4me1^+^/me3^-^ or H3K4me1^high^/me3l^ow^ signature of putative enhancers in ESCs and NESCs cells, respectively, 41% and 46% of which co-localized totally or partially, identifying genetic regions stably marked as enhancers during neural induction (Fig 6D). On the contrary, 52,263 and 40,046 enhancers were ESC- and NESC-specific, respectively (Fig 6D). Overall, the enhancers mapped in ESCs and NESCs covered up to 92% of the 7,405 early developmental enhancers previously described during ESCs differentiation to neurospheres [15] (S8 Fig).

Virtually all mapped enhancers (99% and 97% in ESCs and NESCs respectively) were located >2 kb away from CAGE promoters (Fig 6A). H3K4me1 intensity of total and cell-specific enhancers positively correlated with the expression levels of CAGE promoters located up to 50 kb from enhancer (S6B Fig). In ESCs, the average expression level of CAGE promoters located nearby poised enhancers was significantly lower (p ≤0.01 by t test) than the overall promoter population (S7B Fig). Interestingly, 81, 280 and 180 regions marked as enhancers in ESCs, NESCs and in both cell types respectively, were characterized by active transcription from 86, 339 and 93 non-annotated CAGE promoters or promoters annotated as ncRNAs (Table 1). These included 36% of ESC- and NESC-specific CAGE promoters, and 18% of up- and down-regulated CAGE promoters, which may represent eRNAs differently expressed during ESCs neural induction.

**Table 1:** Transcribed enhancers during ESC neural commitment.

CAGE promoters	in ESC-specific enhancers	in NESC-specific enhancers	in common enhancers
**ESCs**	17	68	22
**NESCs**	13	41	23
**Both**	56	230	48
**Total**	86	339	93

The table lists the non-annotated CAGE promoters or promoters annotated to ncRNAs active in ESCs, NESCs or both cell types (maintaining active transcription during ESC neural commitment), located inside ESC-specific, NESC-specific or common enhancer regions.

To identify low-level bi-directionally transcription, considered as a signature of enhancers transcribing eRNAs, we applied an algorithm recently used to map CAGE-enhancers in a wide range of human and mouse cell types [33, 58], by applying a cut-off of 2 CAGE tags in at least one sample to the entire set of 5,252,347 and 6,501,233 CAGE tags mapped in ESCs and NESCs respectively. Overall, we mapped 1,219 CAGE-enhancers, of which 851 expressed in ESCs and 956 in NESCs (S6 Table). A small percentage (13.4%) of these enhancers was significantly down-regulated (60 enhancers) or up-regulated (103 enhancers) during ESC neural induction (p≤ 0.05 as determined by EdgeR [34], and a FC ≥2). Most CAGE-enhancers (78% and 90% in ESCs and NESCs respectively) carried either a promoter (H3K4me3^+/high^/H3K4me1^-/low^) or an enhancer (H3K4me1^+/high^/H3K4me3^-/low^) signature (S9A Fig). Overall, CAGE-enhancers were expressed at significantly lower levels than all CAGE promoters or CAGE promoters mapped to epigenetically defined enhancers.

To identify the circuitry of TFs operating on cell-specific enhancers, we searched for putative TFBSs in the epigenetically-defined enhancer regions by the HOMER tool [25]. The top ten enriched motifs (p <10^–61^) are shown in Fig 6. As observed for promoters, ESC-specific enhancers showed enrichment for binding motifs of the pluripotency TFs OCT4, SOX2 and NANOG (Fig 6E), while NESC-specific enhancers were enriched for ETS-family, RFX-family and Jun/AP1 factors (Fig 6F). CAGE-enhancers were enriched in specific sets of TFBSs, partially different from those enriched in non-transcribed enhancers (S9B and S9C Fig).

## Discussion

High-throughput approaches are essential for the understanding of transcriptional and epigenetic dynamics orchestrating self-renewal, commitment and differentiation of stem cells. By massively parallel sequencing of Pol-II-transcribed RNAs, we mapped active promoters and quantitatively analyzed their transcriptional activity in a model of human neural commitment, represented by human ESCs induced to differentiate into a relatively well-defined neural stem cell (NESC). We correlated these genome-wide maps of active Pol-II transcription to specifically mapped or publicly available epigenetic annotations of promoters and enhancers, and compared them to discover shared or cell-specific regulatory elements associated to pluripotency and neural lineage restriction.

We used CAGE-seq, a high-throughput technique that allows sequencing of capped Pol-II-dependent transcripts and precise mapping of their TSSs, to obtain a detailed and comprehensive collection of active promoters in ESCs and NESCs. CAGE-seq also provides quantitative estimations of the number of transcripts generated by each promoter, allowing defining cell transcriptomes that we compared to classical Affymetrix profiling of cytoplasmic RNAs. As expected, ESCs and NESCs showed two very distinct transcript phenotypes, with ESCs expressing the pluripotency master regulators OCT4, NANOG and SOX2, and NESCs characterized by the absence of OCT4 and NANOG and the expression of SOX2 together with the marker of neural commitment SOX1, PAX6 and NESTIN. The co-expression of SOX2 and SOX1 confirms the multipotent neural stem cell identity of NESCs. Overall, over 2,400 genes were differentially expressed between the two cell lines with a fold-change of at least 2, and >300 showed strong up-or down-regulation (FC>10) during neural differentiation of ESCs. The correlation between Affymetrix and CAGE-Seq data was acceptable in both cell types (Pearson’s coefficients ~0.6), considering the very different nature of the two datasets: microarray measures mature transcripts in a non-quantitative fashion, while CAGE-seq measures promoter activity that does not necessarily result in the generation of mature mRNAs. Importantly, CAGE-seq identified sets of genes transcribed exclusively in ESCs or NESCs, including non-coding RNAs and novel transcripts not detected by microarrays, and provided a more accurate description of the cell-specific gene expression programs.

Among the genes associated to ESC-specific promoters and to promoters down-regulated during neural induction, we found the pluripotency master regulators, a set of genes recently described to play a role in pluripotency and cell reprogramming [53–56], a variety of genes mainly related to organ and system development, and a small fraction of genes of poorly characterized function. Conversely, NESC-specific and up-regulated promoters were associated to genes involved in the acquisition of neuroectodermal identity, such as PAX6 and MEIS1, known to be transcriptionally repressed in ESCs by the pluripotency master regulators, other genes related to nervous system development, neurogenesis and cell migration, and again a number of functionally uncharacterized genes. Overall, these data are consistent with a progressive restriction of ESC pluripotency towards acquisition of a neural fate.

By CAGE-seq, we mapped around 13,500 promoters in either ESCs or NESCs, a number consistent with previously published data on ESCs and other cell types [59]. The most striking evidence emerging from this analysis is that >90% of the mapped promoters were active in both ESCs and NESCs, and transcribed at roughly comparable levels. These “common” promoters were mostly annotated to protein-coding genes expressed at similar levels in both cells, and are therefore unlikely to play a role in determining their different identities. Consequently, less than 5% of the mapped promoters were cell-specific, i.e., identified only in one cell type, or up- or down-regulated at significant levels (p <0.01) during neural induction. Interestingly, about half of the cell-specific promoters could not be associated to RefSeq RNAs, and were apparently directing the transcription of putative ncRNAs from intergenic regions, or of alternative TSSs inside known genes generating cell-specific transcripts. As an example, we found a novel, NESC-specific promoter 694 bp upstream of the annotated TSS of the SOX1 gene. SOX1 is expressed in NESCs and the alternative TSS may identify a developmentally regulated promoter engaged in SOX1 activation during neural stem cell differentiation. Another example is the alternative promoter usage in the ETS1 gene: the upstream ETS1 promoter is virtually silent in ESCs and upregulated in NESCs, while the downstream promoter is active in ESCs and down-regulated in NESCs. The ETS1 transcription factor has no characterized role in human neural induction, although it is expressed in normal brain cells and in brain cancers [60], where it regulates cell migration and invasion [61]. In developing mouse embryos, ETS1 expression was detected in the hindbrain, neural tube and neural crest [62], while in chicken embryos, ETS1 acts in concert with SOX9 as pan-neural crest regulator for migratory cranial neural crest cells [63]. The promoter analysis suggests a cell-specific regulatory mechanisms acting on ETS1 transcription during neural commitment of ESCs, and encourages additional investigation of ETS1 function in pluripotent and neural stem cells. Overall, we found alternative promoters active in 10% of the transcribed genes in both ESCs and NESCs, indicating that alternative promoter usage is a common mechanism to generate gene expression diversity in these stem cell types, and that alternative transcripts may be important contributors to the definition of NESC identity. Widespread alternative transcriptional initiation was indeed described as a key mechanism used by mammalian cells to confer cell identity during tissue development [64] and differentiation [65, 66].

As mentioned above, a significant fraction of the cell-specific and regulated promoters identified by CAGE-Seq are associated to non-coding transcripts (antisense, miRNAs, lncRNAs, pseudogenes and eRNAs). More than 300 annotated ncRNAs were expressed by ESCs and NESCs, and among them, 20 miRNAs. A panel of miRNAs expressed in ESCs, NESCs and their neuronal progeny was recently described [2], where mature miRNAs were quantified by qRT-PCR and the putative corresponding pre-miRNAs by Northern blotting. Pri-miRNA precursors, and therefore TSSs, were not identified. Although CAGE-seq should in principle allow the identification of miRNA-specific TSSs, we were unable to map transcripts associated to the previously reported ESC- and NESC-specific miRNAs, with the exception of MIR302B and MIR302C, very abundant in human ESCs. Failure to map most of the miRNA promoters may be due to the relative low abundance of the pri-miRNA precursors, which are very rapidly processed to pre-miRNAs and miRNAs [67].

To confirm the identification of promoters and to map enhancer regions in both ESCs and NESCs, we correlated the CAGE-Seq data with genome-wide maps of H3K4me3 and H3K4me1 as distinctive epigenetic signatures of promoters and enhancers. Overall, we observed high co-localization of CAGE promoters and H3K4me3-enriched genomic islands, confirming that the vast majority of active promoters are in common between ESCs and NESCs independently from the technique used to identify them. On the contrary, more than 50% of the >80,000 and >70,000 enhancers mapped as H3K4me1^+/high^ /H3K4me3^-/low^ islands in ESCs and NESCs respectively were cell-specific. These data indicate that most of the relevant genomic changes occurring during neural commitment/differentiation of ESCs are at the level of enhancers rather than promoters. It is therefore a differential enhancer usage that orchestrates qualitative and quantitative changes in the expression of protein-coding and non-coding genes, which most likely determines a pluripotent *vs*. a neural-restricted stem cell identity. Interestingly, a small fraction of unannotated CAGE promoters, or promoters associated to annotated ncRNAs, co-localized with epigenetically-defined enhancers, and some of them were differentially-expressed. Evidence of Pol-II transcription at enhancers was already described in mammalian cells, generating regulatory eRNAs [13, 14, 68]. This analysis therefore defined a collection of novel and known putative eRNAs strongly regulated during neural induction of human ESCs, and possibly involved in its determination. To identify bidirectional capped-RNAs, which were proposed as a signature of active enhancers [11], we reanalyzed our entire set of CAGE tags with the algorithm recently used by the FANTOM consortium to map several thousands of transcribed enhancers (CAGE-enhancers) in a wide range of human and mouse cell types [33, 58] by applying a lower cut-off to detect bidirectional low-level transcripts. The analysis identified >1,200 CAGE-enhancers which carried either a promoter or an enhancer epigenetic signature, a small fraction of which was differentially expressed in ESCs and NESCs and may play some role during neural induction.

The analysis of the epigenetic state of the CAGE promoters in ESCs was particularly interesting. We used publicly available maps of H3K27me3 in ESCs to map bivalent, H3K4me3^+^/H3K27me3^+^ promoters, an epigenetic signature considered as characteristic of promoters poised for transcription. As expected, most (>60%) of the ESC-specific CAGE promoters, promoters down-regulated during NESC induction, and non-specific promoters were epigenetically marked as active (H3K4me3^+^/H3K27me3^-^) in ESCs. Strikingly, however, the vast majority of the NESC-specific promoters (89%) and of those up-regulated during induction (74%) has a bivalent signature in ESCs. This finding is consistent with previous evidence that developmentally-regulated promoters are often poised for transcription in pluripotent cells [10, 15], and suggest that ESCs, or at least the H9 clone, may be pre-committed, or particularly prone, to neuroectodermal differentiation.

Finally, to understand the regulatory circuitry operating on differentially regulated promoters and enhancers, we analyzed putative TFBSs within a region extending from -300 to +100 bp from the most expressed TSS in cell-specific and up- and down-regulated CAGE promotes and in the epigenetically defined enhancer regions. As expected, ESC-specific and down-regulated promoters, and ESC-specific enhancers, showed enrichment for binding motifs of the pluripotency TFs OCT4, SOX2, NANOG and KLF4. Conversely, NESC-specific and up-regulated promoters were essentially enriched for the ubiquitous NFY, MAZ and TBP factors and the RFX-family HTH factors. NESC-specific enhancers were enriched for ETS-family, RFX-family and Jun/AP1 factors. These data correlated well with the cell-specific transcriptomes and promoter usage, and provide evidence that neural commitment is accompanied by dramatic changes in the regulatory circuitry operating on a restricted set of promoters and a much larger set of enhancers.

## Supporting Information

S1 Fig Terminally differentiated neurons derived from NESCs. In the absence of specific growth factors, NESCs give rise to mature neurons positive for neuronal markers such as MAP2 (A), TUBB3 (B), and for GAD65-67 (C), a GABAergic-specific protein. Bar scale 100 μm.
(PDF)

S2 Fig CAGE-mapped TSS distribution and clustering into CAGE promoters. CAGE-TSSs mapped upstream to NANOG (A) and SOX2 (B) genes in ESCs (red) and NESCs (blue); C) At the bottom, CAGE-TSSs and the corresponding CAGE promoters of ETS1 gene in ESCs and NESCs are shown. The upstream ETS1 promoter was upregulated in NESCs (blue bars, blue box) whereas the downstream one was upregulated in ESCs (red bars). In the upper part of the figure, indicated by the arrow, zoom-in on the CAGE promoter of ETS1 in NESCs.
(PDF)

S3 Fig Correlation between CAGE-seq and microarray gene expression analysis. For each gene, the capped RNA amount detected by CAGE-seq (x-axis) was correlated to the mRNA amount evaluated by microarray fluorescent intensity (y-axis), in ESCs (A) and NESCs (B); the same correlation was made on the subset of genes associated to significantly differential promoters, in ESCs (C) and NESCs (D). A modest Person correlation was found between promoter activity and mRNAs quantity, slightly higher for genes whose promoter activity is significantly changing during ESCs-neural commitment.
(PDF)

S4 Fig Networks of genes associated to ESC-specific CAGE promoters. Most of the genes are included in the regulatory pathways mastered by OCT4 and NANOG, and ESC pluripotency in general. Purple arrows indicate the connections between genes based on the Ingenuity Knowledge Base dataset (dotted or solid lines for indirect and direct relationships respectively). Genes involved in IPA canonical pathways (CP) are indicated by grey arrows. The shape of the gene symbol indicates the corresponding protein function, while the color (from white to red) represents the CAGE-seq expression level of the promoter associated to the gene. For a complete IPA legend refer to http://ingenuity.force.com/ipa/articles/Feature_Description/Legend.
(PDF)

S5 Fig Networks of genes associated to down-regulated CAGE promoters. Most of the genes are included in the regulatory pathways of ESC pluripotency, signal transduction and epithelial-mesenchymal transition. Purple arrows indicate the connections between genes based on the Ingenuity Knowledge Base dataset (dotted or solid lines for indirect and direct relationships respectively). Genes involved in IPA canonical pathways (CP) are indicated by grey arrows. The shape of the gene symbol indicates the corresponding protein function, while the color (from white to red) represents the ratio of CAGE-seq expression level of the promoter associated to the gene in ESCs and NESCs. For a complete IPA legend refer to http://ingenuity.force.com/ipa/articles/Feature_Description/Legend
(PDF)

S6 Fig Correlation between histone modification intensity and CAGE promoter expression level. A) Distribution of H3K4me3 peaks around CAGE TSSs (top panels), and the corresponding box-whisker plots (bottom panels). A significant correlation between H3K4me3 intensity and CAGE promoter expression levels was observed. ESC-specific and down-regulated promoters were highly enriched in H3K4me3 in ESCs, compared to NESC-specific and up-regulated promoters. Similarly, NESC-specific and up-regulated promoters showed significantly higher levels of H3K4me3 in NESCs. B) H3K4me1 intensity of total (upper panels) and cell-specific (bottom panels) enhancers close to CAGE promoters (window of ±50 kb). In ESCs H3K4me1 signal of total and cell-specific enhancers is higher around CAGE promoters highly active in ESCs (ESC-specific- and down-regulated promoters) compared to the H3K4me1 intensity around CAGE promoters expressed at lower levels (NESC-specific- and up-regulated promoters) (left panels). Similar results were obtained in NESCs (right panels). Statistical significance was determined by Wilcoxon test with Bonferroni correction (p ≤ 0.05*, p ≤ 0.0001****).
(PDF)

S7 Fig Expression level of CAGE promoters around poised promoter regions and enhancers. A) The graph shows the expression level of CAGE promoters (tpm mean with SEM) carrying an epigenetic signature of active or poised promoter, in a window of 2kb. B) Expression level of CAGE promoters associated to active or poised enhancers in a window of 50 kb. CAGE promoters located around poised promoter regions and enhancers were significantly lower expressed than the overall population of CAGE promoters (p ≤ 0.01**, p ≤ 0.0001****, by unpaired t test).
(PDF)

S8 Fig Comparison between enhancers defined in human ESCs and neural derivatives in the present study, and in a previous study by Rada-Iglesias *et al*. (15). In the previous study a set of 5,118 active and 2,287 poised enhancers were defined in ESCs. Then, 195 poised enhancers in ESCs were defined as active enhancers in ESC-derived neurospheres. We compared the 89,124 ESCs enhancers we mapped with the 5,118 ESCs active (upper left Venn diagram) and 2,287 poised (upper right Venn diagram) enhancers by the previous study. The same comparison was made for the 74,413 NESCs enhancers we mapped and the 195 active enhancers in neurospheres (down).
(PDF)

S9 Fig CAGE-enhancers expressed in ESCs and NESCs. A) The graph shows the epigenetic signature of CAGE-enhancers in ESCs and NESCs. Most CAGE-enhancers carried either a promoter (H3K4me3^+/high^/H3K4me1^-/low^) or an enhancer (H3K4me1^+/high^/H3K4me3^-/low^) signature. B and C) Analysis of putative TFBS enrichment within down- (B) and up- (C) regulated CAGE-enhancers, as determined by HOMER tool.
(PDF)

S1 Materials and Methods (PDF)

S1 Table Down- and up-regulated genes during ESCs neural commitment. (PDF)

S2 Table Table of CAGE promoters associated to ncRNAs in ESCs (red) and NESCs (blue) (PDF)

S3 Table Table of ESC-specific (red), NESC-specific (blue), up- (clear blue) and down-regulated (clear red) CAGE promoters. (PDF)

S4 Table Table of CAGE promoters characterized by epigenetic profile of active promoter (in a window of ± 2 kb from CAGE promoter ends) (PDF)

S5 Table Table of CAGE promoters that transit from a poised to a transcriptionally active state during neural commitment of ESCs. (PDF)

S6 Table Table of CAGE-enhancers expressed in ESCs and NESCs. (PDF)
