# Supplementary material for: Genome-Wide Definition of Promoter and Enhancer Usage during Neural Induction of Human Embryonic Stem Cells
Source: PLoS One. 2015 May 15;10(5):e0126590. doi: 10.1371/journal.pone.0126590 (PMC4433211; doi:10.1371/journal.pone.0126590)
Supplement: S1 Fig — In the absence of specific growth factors, NESCs give rise to mature neurons positive for neuronal markers such as MAP2 (A), TUBB3 (B), and for GAD65-67 (C), a GABAergic-specific protein. Bar scale 100 μm. (PDF) [file pone.0126590.s001.pdf]

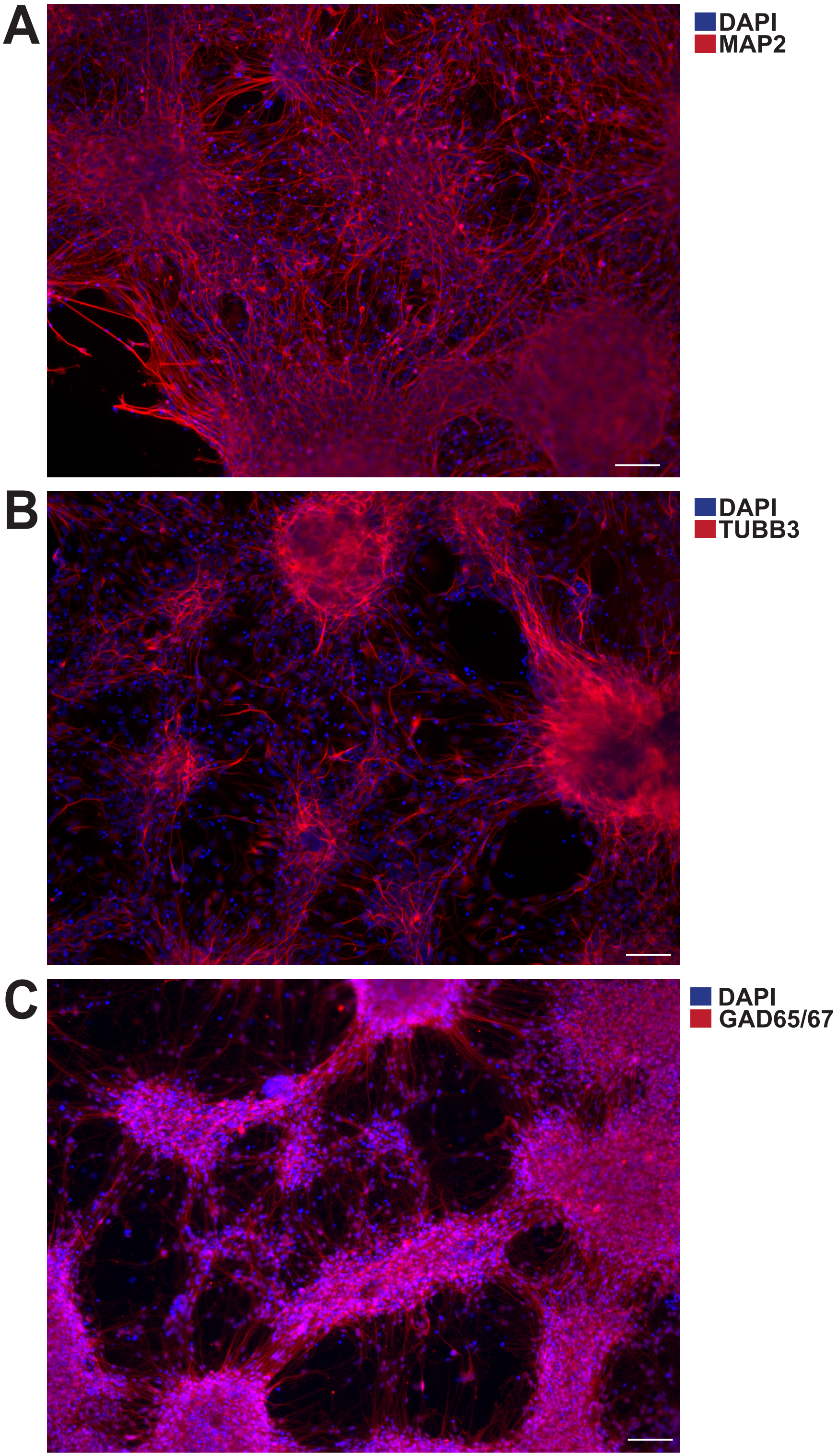

Figure S1. Terminally differentiated neurons derived from NESCs. In the absence of specific growth factors, NESCs give rise to mature neurons positive for neuronal markers such as MAP2 (A), TUBB3 (B), and for GAD65-67 (C), a GABAergic-specific protein. Bar scale 100 μm.
