# Supplementary material for: Genome-Wide Definition of Promoter and Enhancer Usage during Neural Induction of Human Embryonic Stem Cells
Source: PLoS One. 2015 May 15;10(5):e0126590. doi: 10.1371/journal.pone.0126590 (PMC4433211; doi:10.1371/journal.pone.0126590)
Supplement: S2 Fig — CAGE-TSSs mapped upstream to NANOG (A) and SOX2 (B) genes in ESCs (red) and NESCs (blue); C) At the bottom, CAGE-TSSs and the corresponding CAGE promoters of ETS1 gene in ESCs and NESCs are shown. The upstream ETS1 promoter was upregulated in NESCs (blue bars, blue box) whereas the downstream one was upregulated in ESCs (red bars). In the upper part of the figure, indicated by the arrow, zoom-in on the CAGE promoter of ETS1 in NESCs. (PDF) [file pone.0126590.s002.pdf]

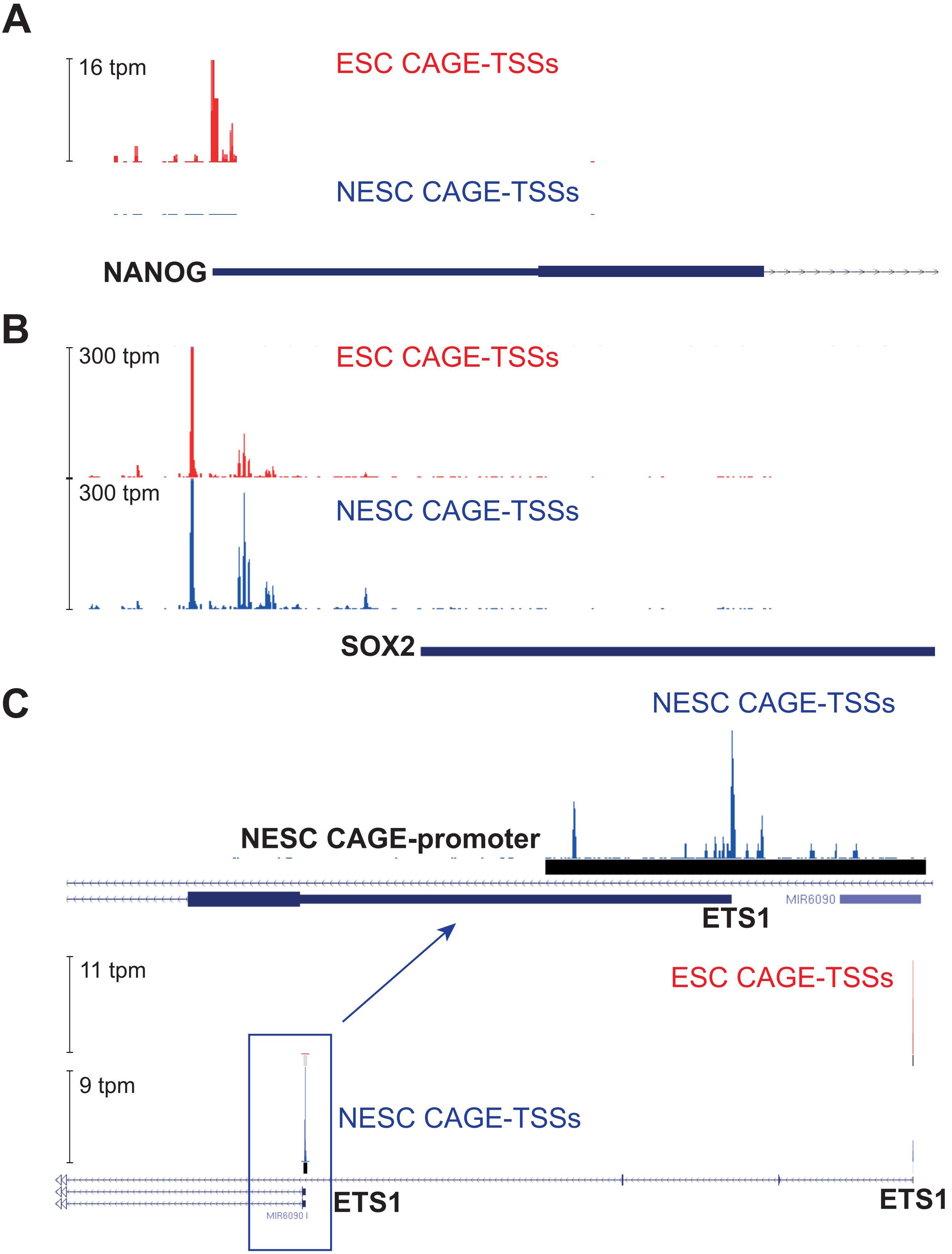

Figure S2. CAGE-mapped TSS distribution and clustering into CAGE promoters. CAGE-TSSs mapped upstream to NANOG (A) and SOX2 (B) genes in ESCs (red) and NESCs (blue); C) At the bottom, CAGE-TSSs and the corresponding CAGE promoters of ETS1 gene in ESCs and NESCs are shown. The upstream ETS1 promoter was upregulated in NESCs (blue bars, blue box) whereas the downstream one was upregulated in ESCs (red bars). In the upper part of the figure, indicated by the arrow, zoom-in on the CAGE promoter of ETS1 in NESCs.
