# Supplementary material for: Genome-Wide Definition of Promoter and Enhancer Usage during Neural Induction of Human Embryonic Stem Cells
Source: PLoS One. 2015 May 15;10(5):e0126590. doi: 10.1371/journal.pone.0126590 (PMC4433211; doi:10.1371/journal.pone.0126590)
Supplement: S3 Fig — For each gene, the capped RNA amount detected by CAGE-seq (x-axis) was correlated to the mRNA amount evaluated by microarray fluorescent intensity (y-axis), in ESCs (A) and NESCs (B); the same correlation was made on the subset of genes associated to significantly differential promoters, in ESCs (C) and NESCs (D). A modest Person correlation was found between promoter activity and mRNAs quantity, slightly higher for genes whose promoter activity is significantly changing during ESCs-neural commitment. (PDF) [file pone.0126590.s003.pdf]

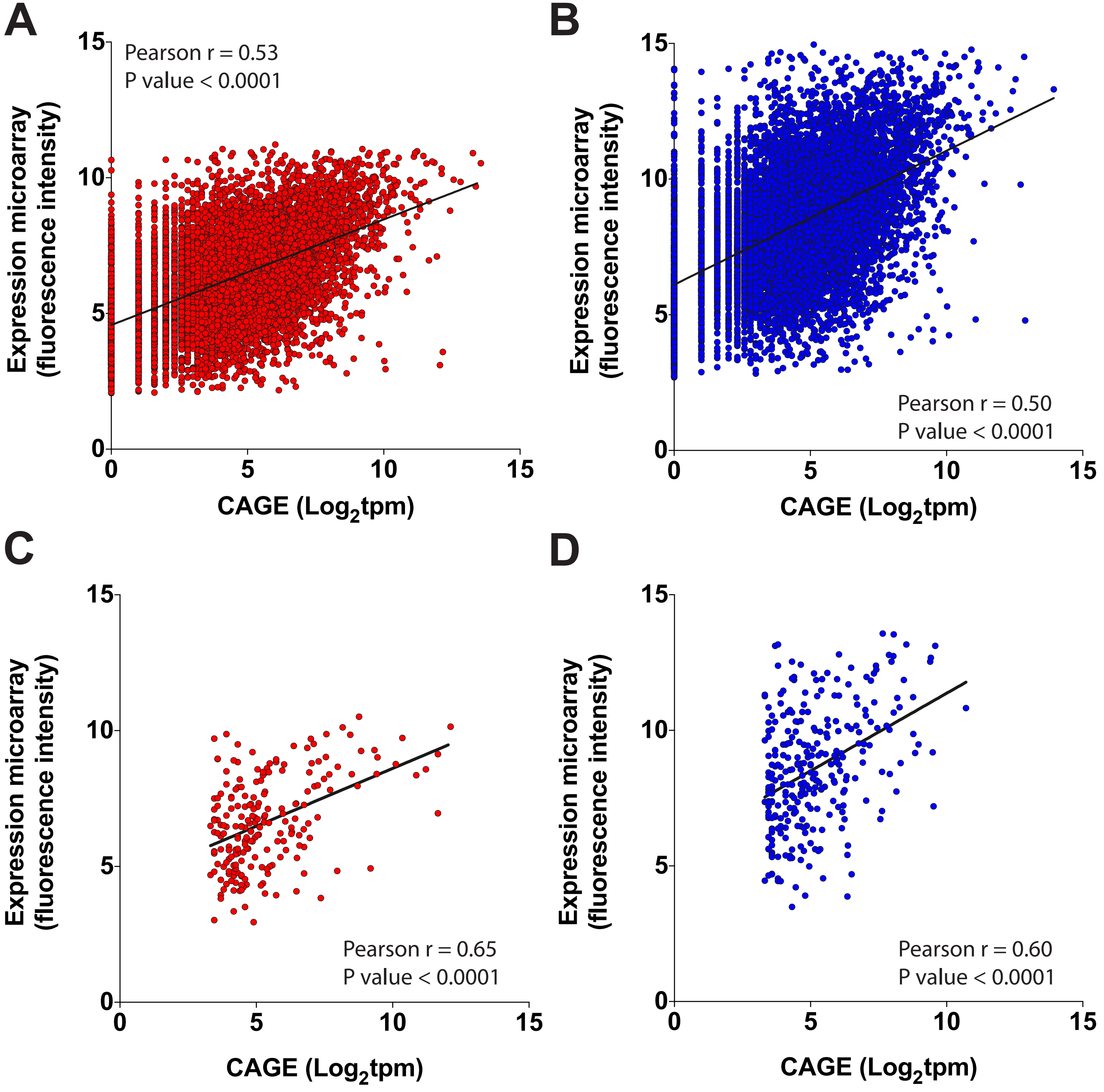

Figure S3. Correlation between CAGE-seq and microarray gene expression analysis. For each gene, the capped RNA amount detected by CAGE-seq (x-axis) was correlated to the mRNA amount evaluated by microarray fluorescent intensity (y-axis), in ESCs (A) and NESCs (B); the same correlation was made on the subset of genes associated to significantly differential promoters, in ESCs (C) and NESCs (D). A modest Person correlation was found between promoter activity and mRNAs quantity, slightly higher for genes whose promoter activity is significantly changing during ESCs-neural commitment.
