# Supplementary material for: Genome-Wide Definition of Promoter and Enhancer Usage during Neural Induction of Human Embryonic Stem Cells
Source: PLoS One. 2015 May 15;10(5):e0126590. doi: 10.1371/journal.pone.0126590 (PMC4433211; doi:10.1371/journal.pone.0126590)
Supplement: S4 Fig — Most of the genes are included in the regulatory pathways mastered by OCT4 and NANOG, and ESC pluripotency in general. Purple arrows indicate the connections between genes based on the Ingenuity Knowledge Base dataset (dotted or solid lines for indirect and direct relationships respectively). Genes involved in IPA canonical pathways (CP) are indicated by grey arrows. The shape of the gene symbol indicates the corresponding protein function, while the color (from white to red) represents the CAGE-seq expression level of the promoter associated to the gene. For a complete IPA legend refer to http://ingenuity.force.com/ipa/articles/Feature_Description/Legend. (PDF) [file pone.0126590.s004.pdf]

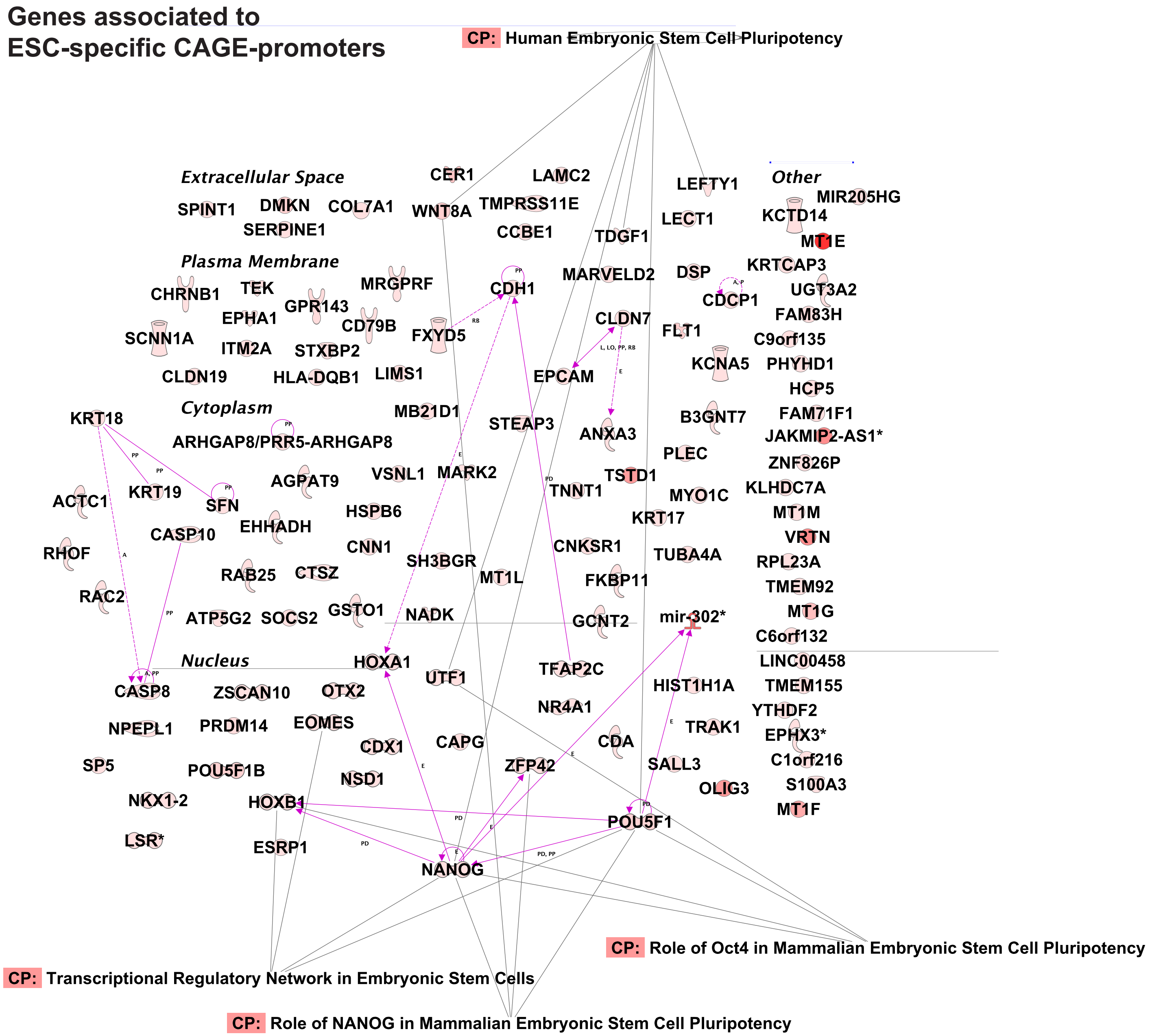

Figure S4. Networks of genes associated to ESC-specific CAGE promoters. Most of the genes are included in the regulatory pathways mastered by OCT4 and NANOG, and ESC pluripotency in general. Purple arrows indicate the connections between genes based on the Ingenuity ® Knowledge Base dataset (dotted or solid lines for indirect and direct relationships respectively). Genes involved in IPA canonical pathways (CP) are indicated by grey arrows. The shape of the gene symbol indicates the corresponding protein function, while the colour (from white to red) represents the CAGE-seq expression level of the promoter associated to the gene. For a complete IPA legend refer to http://ingenuity.force.com/ipa/articles/Feature_Description/Legend.
