# Supplementary material for: Genome-Wide Definition of Promoter and Enhancer Usage during Neural Induction of Human Embryonic Stem Cells
Source: PLoS One. 2015 May 15;10(5):e0126590. doi: 10.1371/journal.pone.0126590 (PMC4433211; doi:10.1371/journal.pone.0126590)
Supplement: S5 Fig — Most of the genes are included in the regulatory pathways of ESC pluripotency, signal transduction and epithelial-mesenchymal transition. Purple arrows indicate the connections between genes based on the Ingenuity Knowledge Base dataset (dotted or solid lines for indirect and direct relationships respectively). Genes involved in IPA canonical pathways (CP) are indicated by grey arrows. The shape of the gene symbol indicates the corresponding protein function, while the color (from white to red) represents the ratio of CAGE-seq expression level of the promoter associated to the gene in ESCs and NESCs. For a complete IPA legend refer to http://ingenuity.force.com/ipa/articles/Feature_Description/Legend (PDF) [file pone.0126590.s005.pdf]

Genes associated to down-regulated CAGE-promoters

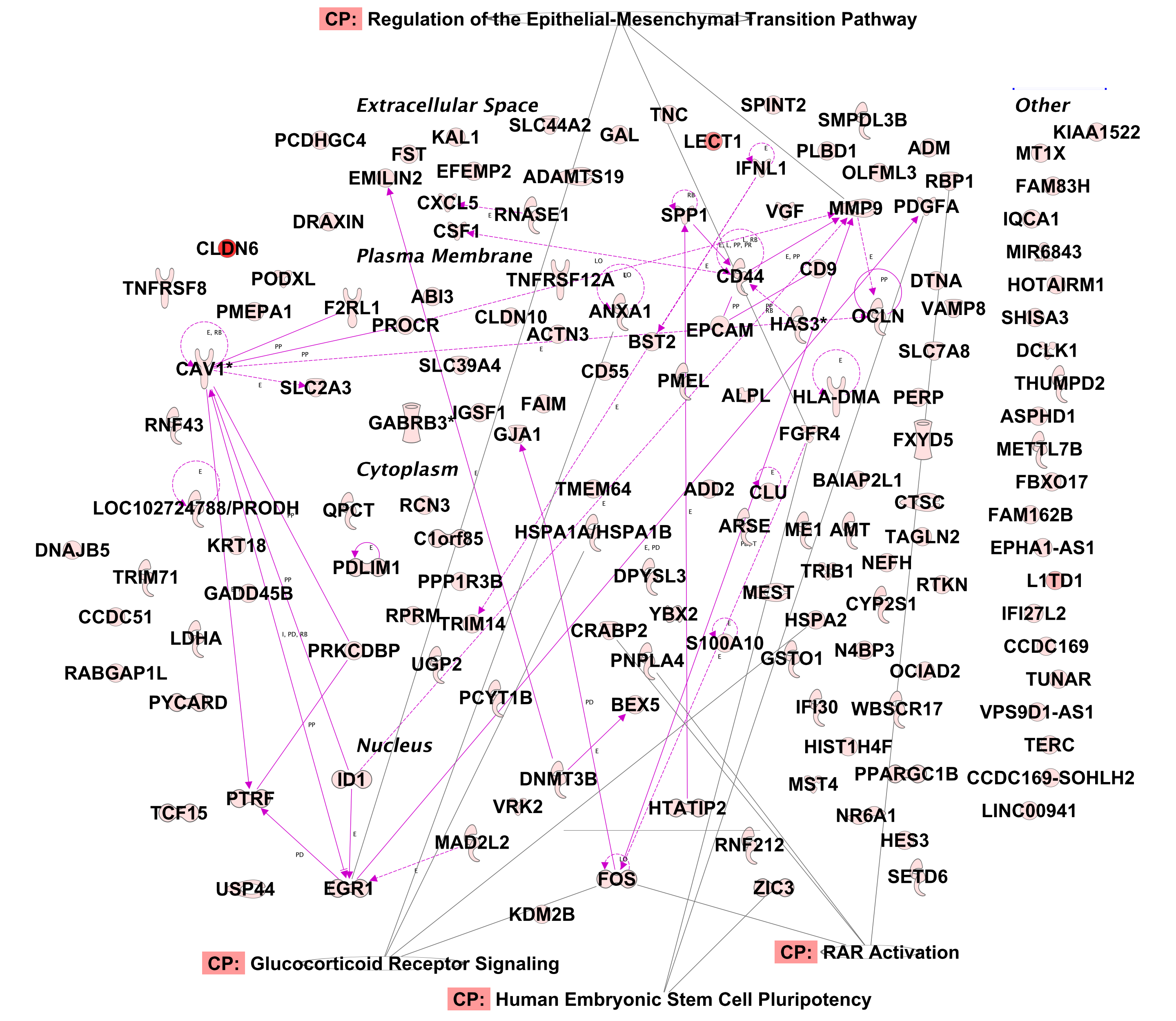

Figure S5. Networks of genes associated to down-regulated CAGE promoters. Most of the genes are included in the regulatory pathways of ESC pluripotency, signal transduction and epithelial-mesenchymal transition. Purple arrows indicate the connections between genes based on the Ingenuity ® Knowledge Base dataset (dotted or solid lines for indirect and direct relationships respectively). Genes involved in IPA canonical pathways (CP) are indicated by grey arrows. The shape of the gene symbol indicates the corresponding protein function, while the colour (from white to red) represents the ratio of CAGE-seq expression level of the promoter associated to the gene in ESCs and NESCs. For a complete IPA legend refer to http://ingenuity.force.com/ipa/articles/Feature_Description/Legend.
