# Supplementary material for: Genome-Wide Definition of Promoter and Enhancer Usage during Neural Induction of Human Embryonic Stem Cells
Source: PLoS One. 2015 May 15;10(5):e0126590. doi: 10.1371/journal.pone.0126590 (PMC4433211; doi:10.1371/journal.pone.0126590)
Supplement: S6 Fig — A) Distribution of H3K4me3 peaks around CAGE TSSs (top panels), and the corresponding box-whisker plots (bottom panels). A significant correlation between H3K4me3 intensity and CAGE promoter expression levels was observed. ESC-specific and down-regulated promoters were highly enriched in H3K4me3 in ESCs, compared to NESC-specific and up-regulated promoters. Similarly, NESC-specific and up-regulated promoters showed significantly higher levels of H3K4me3 in NESCs. B) H3K4me1 intensity of total (upper panels) and cell-specific (bottom panels) enhancers close to CAGE promoters (window of ±50 kb). In ESCs H3K4me1 signal of total and cell-specific enhancers is higher around CAGE promoters highly active in ESCs (ESC-specific- and down-regulated promoters) compared to the H3K4me1 intensity around CAGE promoters expressed at lower levels (NESC-specific- and up-regulated promoters) (left panels). Similar results were obtained in NESCs (right panels). Statistical significance was determined by Wilcoxon test with Bonferroni correction (p ≤ 0.05*, p ≤ 0.0001****). (PDF) [file pone.0126590.s006.pdf]

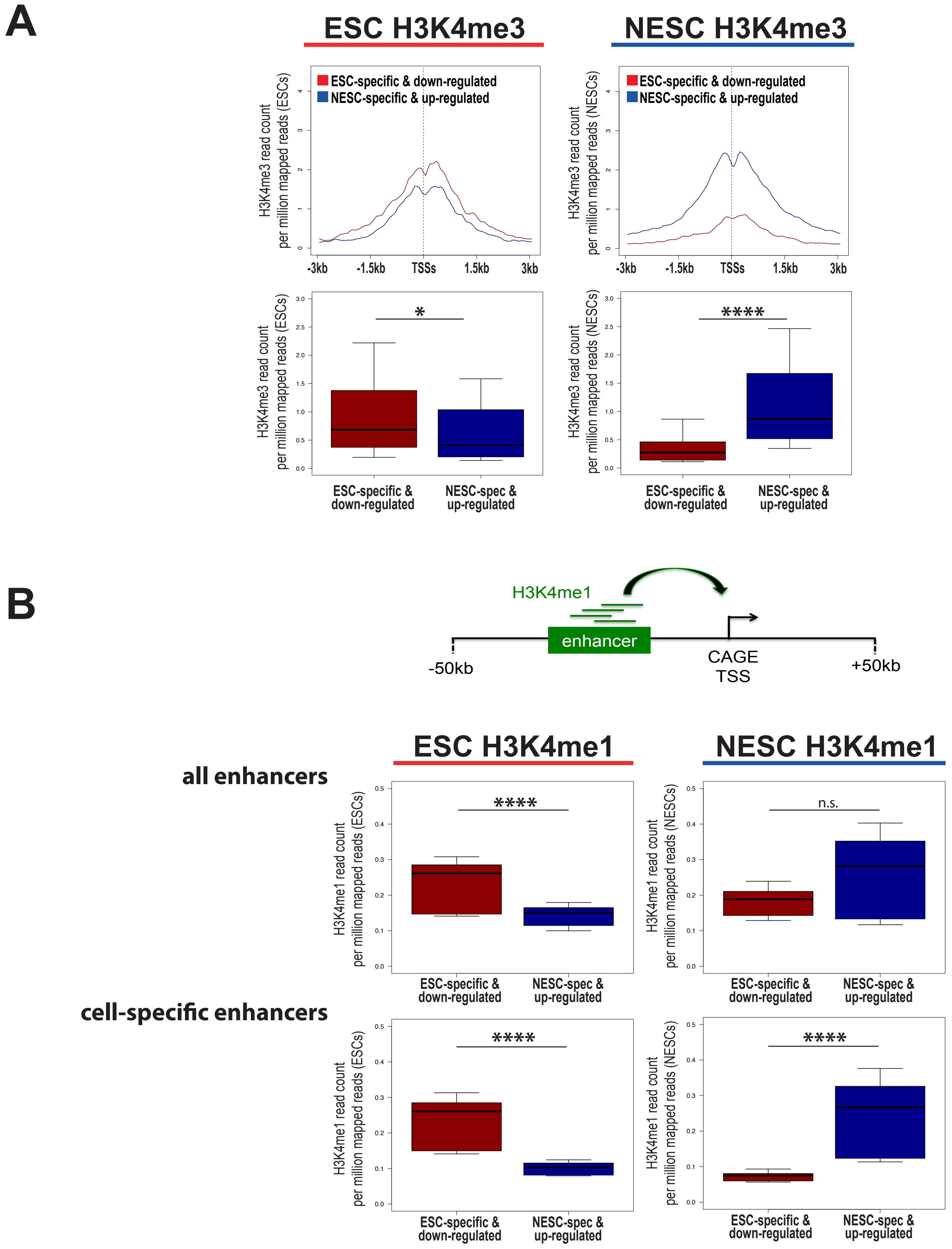

Figure S6. Correlation between histone modification intensity and CAGE promoter expression level. A) Distribution of H3K4me3 peaks around CAGE TSSs (top panels), and the corresponding box-whisker plots (bottom panels). A significant correlation between H3K4me3 intensity and CAGE promoter expression levels was observed. ESC-specific and down-regulated promoters were highly enriched in H3K4me3 in ESCs, compared to NESC-specific and up-regulated promoters. Similarly, NESC-specific and up-regulated promoters showed significantly higher levels of H3K4me3 in NESCs. B) H3K4me1 intensity of total (upper panels) and cell-specific (bottom panels) enhancers close to CAGE promoters (window of ±50kb). In ESCs H3K4me1 signal of total and cell-specific enhancers is higher around CAGE promoters highly active in ESCs (ESC-specific- and down-regulated promoters) compared to the H3K4me1 intensity around CAGE promoters expressed at lower levels (NESC-specific- and up-regulated promoters) (left panels). Similar results were obtained in NESCs (right panels). Statistical significance was determined by Wilcoxon test with Bonferroni correction of p value ($p \leq 0.05$*, $p \leq 0.0001$****).
