# Supplementary material for: Genome-Wide Definition of Promoter and Enhancer Usage during Neural Induction of Human Embryonic Stem Cells
Source: PLoS One. 2015 May 15;10(5):e0126590. doi: 10.1371/journal.pone.0126590 (PMC4433211; doi:10.1371/journal.pone.0126590)
Supplement: S7 Fig — A) The graph shows the expression level of CAGE promoters (tpm mean with SEM) carrying an epigenetic signature of active or poised promoter, in a window of 2kb. B) Expression level of CAGE promoters associated to active or poised enhancers in a window of 50 kb. CAGE promoters located around poised promoter regions and enhancers were significantly lower expressed than the overall population of CAGE promoters (p ≤ 0.01**, p ≤ 0.0001****, by unpaired t test). (PDF) [file pone.0126590.s007.pdf]

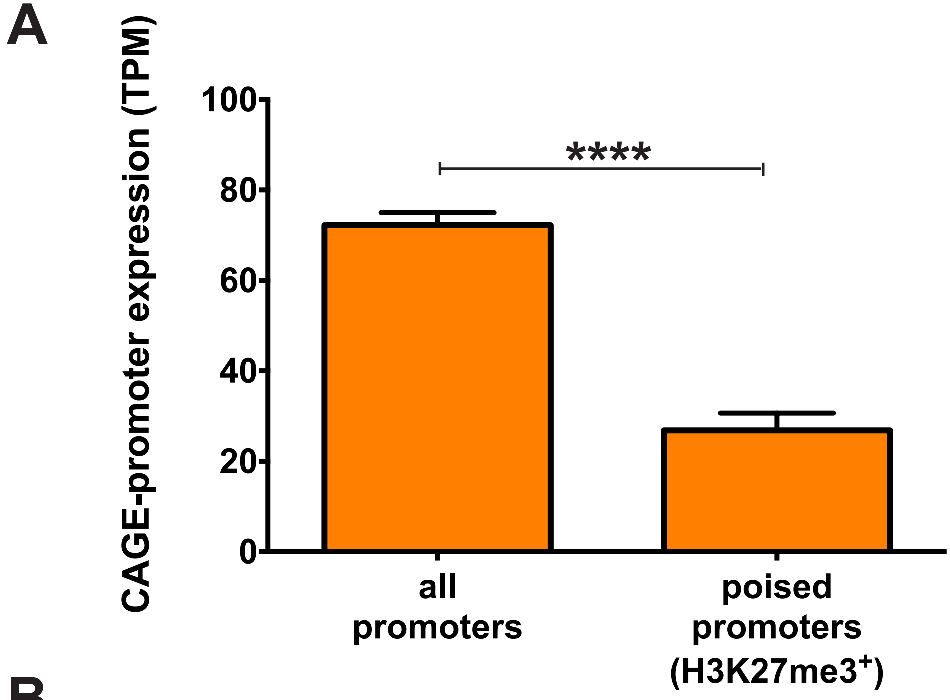

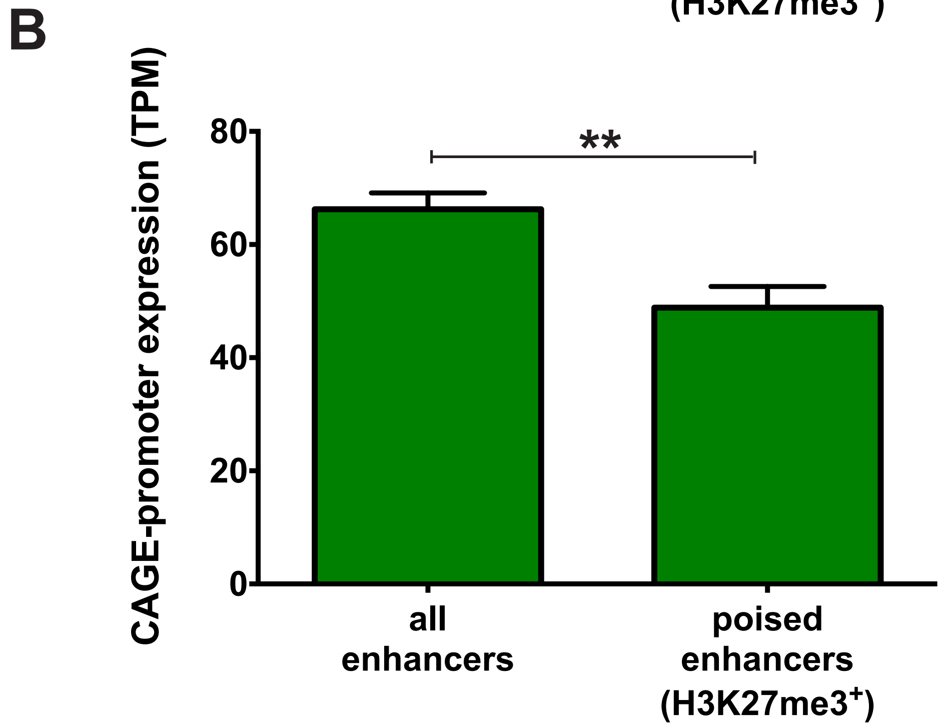

Figure S7. Expression level of CAGE promoters around poised promoter regions and enhancers. A) The graph shows the expression level of CAGE promoters (tpm mean with SEM) carrying an epigenetic signature of active or poised promoter, in a window of 2kb. B) Expression level of CAGE promoters associated to active or poised enhancers in a window of 50 kb. CAGE promoters located around poised promoter regions and enhancers were significantly lower expressed than the overall population of CAGE promoters ($p \leq 0.01$**, $p \leq 0.0001$****, by unpaired t test).
