# Supplementary material for: Genome-Wide Definition of Promoter and Enhancer Usage during Neural Induction of Human Embryonic Stem Cells
Source: PLoS One. 2015 May 15;10(5):e0126590. doi: 10.1371/journal.pone.0126590 (PMC4433211; doi:10.1371/journal.pone.0126590)
Supplement: S8 Fig — In the previous study a set of 5,118 active and 2,287 poised enhancers were defined in ESCs. Then, 195 poised enhancers in ESCs were defined as active enhancers in ESC-derived neurospheres. We compared the 89,124 ESCs enhancers we mapped with the 5,118 ESCs active (upper left Venn diagram) and 2,287 poised (upper right Venn diagram) enhancers by the previous study. The same comparison was made for the 74,413 NESCs enhancers we mapped and the 195 active enhancers in neurospheres (down). (PDF) [file pone.0126590.s008.pdf]

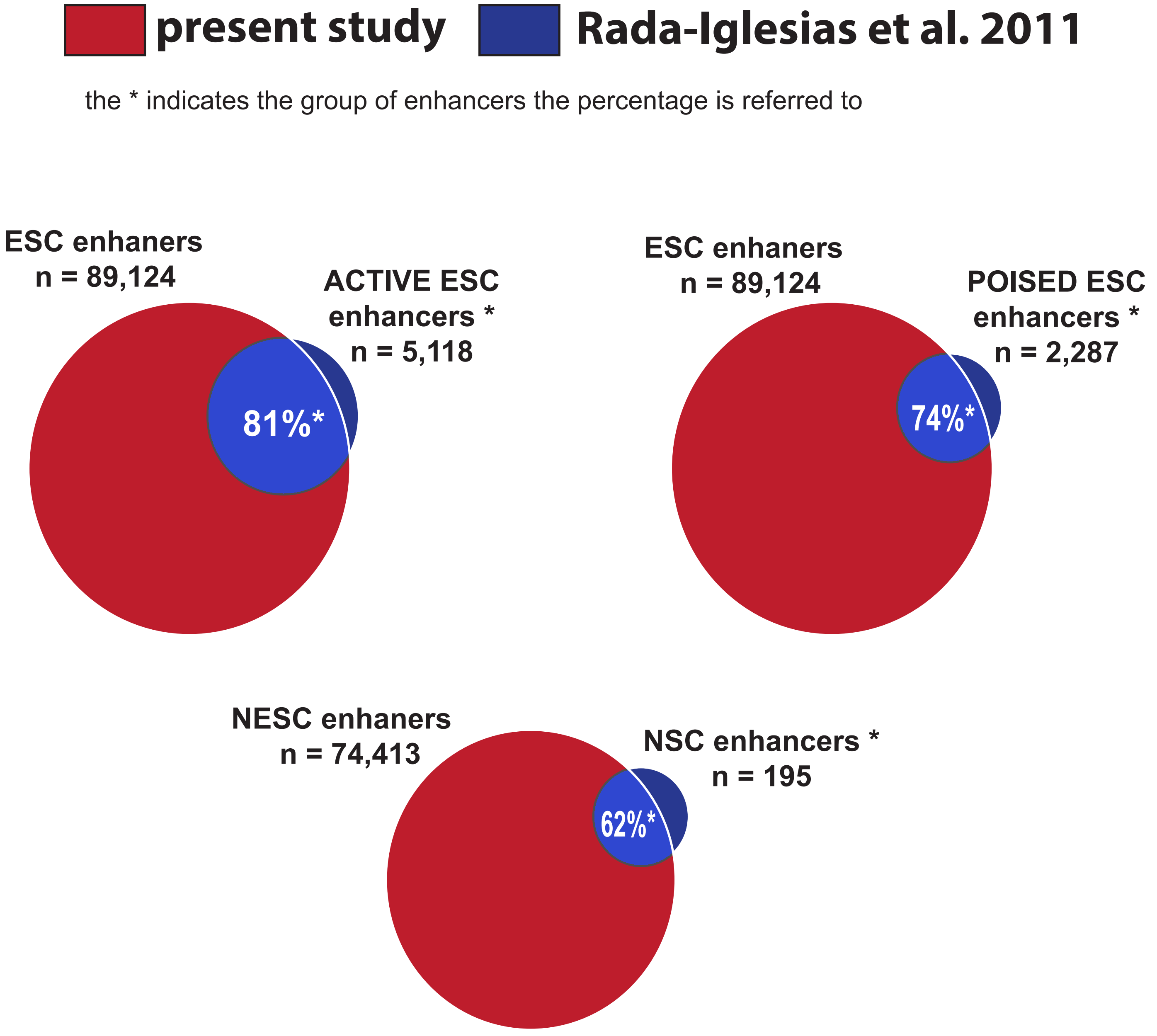

Figure S8. Comparison between enhancers defined in human ESCs and neural derivatives in the present study, and in a previous study by Rada-Iglesias [15]. In the previous study a set of 5,118 active and 2,287 poised enhancers were defined in ESCs. Then, 195 poised enhancers in ESCs were defined as active enhancers in ESC-derived neurospheres. We compared the 89,124 ESCs enhancers we mapped with the 5,118 ESCs active (upper left Venn diagram) and 2,287 poised (upper right Venn diagram) enhancers by the previous study. The same comparison was made for the 74,413 NESCs enhancers we mapped and the 195 active enhancers in neurospheres (down).
