# Supplementary material for: Genome-Wide Definition of Promoter and Enhancer Usage during Neural Induction of Human Embryonic Stem Cells
Source: PLoS One. 2015 May 15;10(5):e0126590. doi: 10.1371/journal.pone.0126590 (PMC4433211; doi:10.1371/journal.pone.0126590)
Supplement: S9 Fig — A) The graph shows the epigenetic signature of CAGE-enhancers in ESCs and NESCs. Most CAGE-enhancers carried either a promoter (H3K4me3+/high/H3K4me1-/low) or an enhancer (H3K4me1+/high/H3K4me3-/low) signature. B and C) Analysis of putative TFBS enrichment within down- (B) and up- (C) regulated CAGE-enhancers, as determined by HOMER tool. (PDF) [file pone.0126590.s009.pdf]

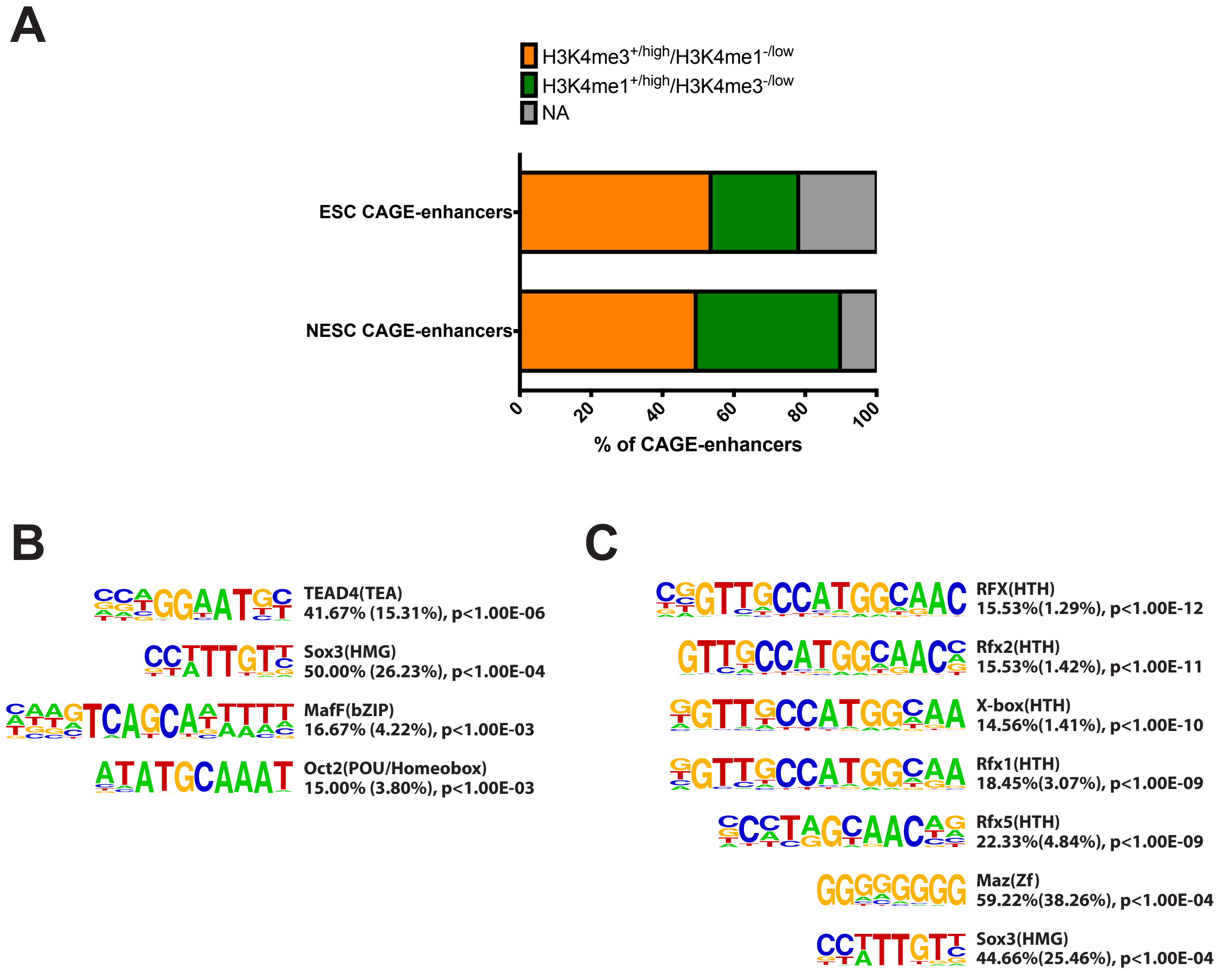

Figure S9. CAGE-enhancers expressed in ESCs and NESCs. A) The graph shows the epigenetic signature of CAGE-enhancers in ESCs and NESCs. Most CAGE-enhancers carried either a promoter ($H3K4me3^{+/high}/H3K4me1^{-/low}$) or an enhancer ($H3K4me1^{+/high}/H3K4me3^{-/low}$) signature. B and C) Analysis of putative TFBS enrichment within down- (B) and up- (C) regulated CAGE-enhancers, as determined by HOMER tool.
