# Supplementary material for: Genome-Wide Definition of Promoter and Enhancer Usage during Neural Induction of Human Embryonic Stem Cells
Source: PLoS One. 2015 May 15;10(5):e0126590. doi: 10.1371/journal.pone.0126590 (PMC4433211; doi:10.1371/journal.pone.0126590)
Supplement: S1 Materials and Methods — (PDF) [file pone.0126590.s010.pdf]

Supporting Materials and Methods

Cell cultures

ESCs (H9 NIH code WA09, ISL1 Ds-Red) were kindly provided by the group of K.R. Chien. They were maintained on irradiated mouse embryonic fibroblasts (MEFs) at 5% CO_2 in Knockout-DMEM (Invitrogen), containing 20% FCS, 1% non-essential amino acids, 1 mM glutamine, 0.1 mM ß-mercaptoethanol and 10 ng/mL FGF2 (Invitrogen). Cultures were passaged 1:6 every 5 days. Neural differentiation was induced by the method of embryoid bodies following the protocol published in [1]. Briefly, 4-days embryoid bodies were transferred to polyornithine-coated dishes and propagated in N_2-supplemented DMEM/F12 (Invitrogen). Within 10 days, neural differentiation appears as small rosettes, with a columnar shape, which were mechanically isolated. Isolated clusters where propagated for 1 to 7 days as free-floating neurospheres in DMEM/F12 containing 10 ng/mL FGF2 (R&D Systems), dissociated into single cells in trypsin/EDTA and plated on polyornithine/laminin pre-coated plastic dishes. Media was changed to neural stem cell medium containing DMEM/F12, 20 µg/mL insulin (Sigma–Aldrich), 1.6 g/L glucose, 10 ng/mL FGF2 (R&D Systems), 10 ng/mL EGF (R&D Systems), and 1 µl/ml B27 supplement (Invitrogen). Cells were further passaged at very high density one day after reaching confluence.

Microarray gene expression profiling

Total RNA was extracted from 1–2 10^6 cells from triplicate cultures of ESCs and NESCs, transcribed into biotinylated cRNA and hybridized onto GeneChip® HG-U133

Plus 2.0 Arrays (Affymetrix) according to the manufacturer protocol (Affymetrix, Santa Clara, CA). Chips were scanned using an Affymetrix GeneChip Scanner 3000. Signals were converted to expression values by robust multi-array average procedure (RMA algorithm, [2]) and HG-U133 Plus 2.0 custom Chip Definition Files (CDF) based on GeneAnnot [3] (CDF Version 2.2.0, GeneCards Version 3.04, GeneAnnot Version 2.0) in R (http://www.r-project.org/). Intensity levels for a total of 19,204 custom probe sets were background-adjusted and normalized using quantile normalization, and log2 expression values calculated using median polish summarization. Gene differentially expressed between ESCs and NESCs were identified using the two-class comparison procedure of the Significance Analysis of Microarray (SAM, [4], *samr* R package). In SAM, the percentage of false positive predictions was estimated with 100 permutations and significant genes defined setting the q-value threshold equal to 0 (or ≤ 0.01) and varying the Fold Change level (FC) between 2 and 10. Genes and samples were grouped using hierarchical clustering with Pearson correlation and centroid as distance metric and linkage, respectively, and clusters displayed as heatmaps using dChip software (www.hsph.harvard.edu/cli/complab/dchip/).

Gene functional annotation

Functional annotation of differentially expressed genes was performed by the Functional Annotation Tool of the Database for Annotation, Visualization and Integrated Discovery (DAVID) v6.7 (http://david.abcc.ncifcrf.gov/home.jsp). This tool suite provides Gene Ontology (GO) term enrichment analysis to highlight the most relevant GO terms associated with a given gene list. DAVID statistics is based on EASE Score, a modified one-tail Fisher Exact P-Value. GO-term enrichments associated were considered significant at p-value ≤ 0.05, after Benjamini correction for multiple testing.

CAGE-seq promoter profiling

CAGE-seq was performed by DNAFORM Inc. at RIKEN Omics Science Center. The samples were prepared according to the OP-SOLEXA-96CAGE-v.3.0 [5]. Briefly, the cDNA synthesis was performed starting from 5 μg of total RNA and RT random N15 primers. RNA/cDNA hybrids were purified with Agencourt RNAClean XP (BECKMAN) and eluted from the beads. Capped-RNA was biotinylated and treated by RNaseOne. cDNA hybrid with biotinylated capped-RNA was selected with cap-trapper method [5], and the cDNA was released from beads. A sample-specific linker containing a recognition site for the type III restriction-modification enzyme EcoP15I was ligated to the single-strand cDNA. After ligation, the cDNA was purified with Agencourt AMPure XP (BECKMAN) and the 2nd strand synthesis is performed. The resulting double-stranded cDNA was purified and cleaved by EcoP15I (NEB). After heat inactivation, the 2nd linker was ligated to the CAGE tags with T4 DNA ligase (NEB). The CAGE tags were separated from unmodified DNA with MPG Streptavidin beads. The DNA fragments were amplified in a PCR step by using linker-specific primers and the PCR products was purified and adjusted in concentration for Illumina Solexa sequencing. The four-color DNA Sequencing-By-Synthesis (SBS) data were generated by Illumina (R) Genome Analyzer. The sequencing run and the base call analysis were performed according to the manufacturer's protocol Illumina Sequencing Kit v4. After the sequencing, sequence raw data were generated with processing by Genome Analyzer IIx. For CAGE tags mapping to multiple genome locations, a weighting strategy based on the number of CAGE tags within a 200-bp interval around each candidate mapping location was applied [6]. Equal weights were used if no unique tags were found within the 200-bp region for all candidate mapping locations. Level-1

promoters (TSSs) were created summing the weighted number of CAGE tags at each genome position with at least one CAGE tag in at least one experimental condition; other mapping positions were discarded. Level-1 promoters were clustered into Level-2 promoters (CAGE-promoters) if closer than 20 bp on the same strand and if resulting an expression level of at least 10 tpm (tags per million) in at least one experimental condition; other promoters were dropped. Tpm were calculated for each level-1 and level-2 promoter dividing the number of CAGE tags of each promoter in each experimental condition by the total number of mapped CAGE tags in that condition, and multiplied by 10^6. For promoter annotation, RefSeq and Gencode (release 14) genes and transcripts coordinates were downloaded from the UCSC table browser for the Hg19 genome assembly. For each set of annotations, the TSS of the annotation with the smallest distance to the CAGE-promoter on the same chromosome strand was found and if this distance was less than 400 bp, the promoter was associated to the gene or transcript. The chi-squared test was used as statistical method to determine significance of differentially expressed CAGE-promoters. A p-value threshold of 0.01 was used to determine statistically significant differentially expressed promoters. For some analysis a FC>3 was also imposed to consider CAGE-promoters as differentially used (up- and down-regulated promoters).

Chip-seq preparation and sequencing

Chromatin was prepared from NESCs after cross-linking for 10’ at rt with 1% formaldehyde-containing medium, using truChIP™ High Cell Chromatin Shearing Kit with SDS Shearing Buffer (Covaris). Nuclear extracts were sonicated to obtain DNA fragments averaging 200 bp in length and the equivalent of 5 10^6 cells was immunoprecipitated overnight with 10 μg of rabbit antibodies against H3K4me1

(ab8895, Abcam) and H3K4me3 (ab8580, Abcam) as previously described [7]. DNA fragments derived from chromatin sonication of nuclear extracts were used for sequencing library preparation. Illumina libraries were prepared from 10 ng of immunoprecipitated DNA (IP) and control DNA (INPUT: nuclear extracts sonicated but non-immunoprecipitated) following the Illumina Truseq ChIP sample preparation kit. The libraries were checked by capillary electrophoresis by Agilent Bioanalyzer 2100 with the High sensitivity DNA assay and quantified with Quant-iT™ PicoGreen ® dsDNA Kits (Invitrogen) by Nanodrop Fluorimeter and by Real Time qPCR using the KAPA library quantification kit (KAPABiosystems). Each library was sequenced in one lane of a single strand 50 bp GAIIx Illumina Run.

References

1. Koch P, Opitz T, Steinbeck JA, Ladewig J and Brustle O (2009) A rosette-type, self-renewing human ES cell-derived neural stem cell with potential for in vitro instruction and synaptic integration. Proc Natl Acad Sci U S A 106: 3225-3230.
2. Irizarry RA, Hobbs B, Collin F, Beazer-Barclay YD, Antonellis KJ, et al. (2003) Exploration, normalization, and summaries of high density oligonucleotide array probe level data. Biostatistics 4: 249-264.
3. Ferrari F, Bortoluzzi S, Coppe A, Sirota A, Safran M, et al. (2007) Novel definition files for human GeneChips based on GeneAnnot. BMC Bioinformatics 8: 446.
4. Tusher VG, Tibshirani R and Chu G (2001) Significance analysis of microarrays applied to the ionizing radiation response. Proc Natl Acad Sci U S A 98: 5116-5121.
5. Carninci P, Westover A, Nishiyama Y, Ohsumi T, Itoh M, et al. (1997) High efficiency selection of full-length cDNA by improved biotinylated cap trapper. DNA research : an international journal for rapid publication of reports on genes and genomes 4: 61-66.
6. Faulkner GJ, Forrest AR, Chalk AM, Schroder K, Hayashizaki Y, et al. (2008) A rescue strategy for multimapping short sequence tags refines surveys of transcriptional activity by CAGE. Genomics 91: 281-288.
7. Cattoglio C, Pellin D, Rizzi E, Maruggi G, Corti G, et al. (2010) High-definition mapping of retroviral integration sites identifies active regulatory elements in human multipotent hematopoietic progenitors. Blood 116: 5507-5517.
