# Supplementary material for: Genome-Wide Definition of Promoter and Enhancer Usage during Neural Induction of Human Embryonic Stem Cells
Source: PLoS One. 2015 May 15;10(5):e0126590. doi: 10.1371/journal.pone.0126590 (PMC4433211; doi:10.1371/journal.pone.0126590)
Supplement: S1 Table — (PDF) [file pone.0126590.s011.pdf]

TABLE S1

Down- and up-regulated genes during ESCs neural commitment
SAM q value=0, Fold Change=10

RED downregulated genes
BLUE upregulated genes

Gene Card ID: Gene Card identification number of the Affymetrix probeset
Entrez ID: official gene identification number
Symbol: gene official symbol provided by HGNC (HUGO Gene Nomenclature Committee)
Description: gene description by NCBI (National Center for Biotechnology Information)

Gene Card ID	Entrez ID	Symbol	Description
GC12P007941_at	79923	NANOG	Nanog homeobox
GC01P062660_at	54596	L1TD1	LINE-1 type transposase domain containing 1
GC02P171571_at	389058	SP5	Sp5 transcription factor
GC01M161007_at	100131187	TSTD1	thiosulfate sulfurtransferase (rhodanese)-like domain containing 1
GC17M039679_at	3880	KRT19	keratin 19
GC11P068451_at	51083	GAL	galanin prepropeptide
GC07M019958_at	NA	ENSG00000243004	NA
GC13M053277_at	11061	LECT1	leukocyte cell derived chemotaxin 1
GC09P072435_at	138255	C9orf135	chromosome 9 open reading frame 135
GC07M027099_at	3198	HOXA1	homeobox A1
GC18P076766_at	27164	SALL3	sal-like 3 (Drosophila)
GC02P047572_at	4072	EPCAM	epithelial cell adhesion molecule
GC03P046594_at	6997	TDGF1	teratocarcinoma-derived growth factor 1
GC11M068771_at	116535	MRGPRF	MAS-related GPR, member F
GC02P037571_at	25797	QPCT	glutaminyl-peptide cyclotransferase
GC06P007541_at	1832	DSP	desmoplakin
GC05P052812_at	10468	FST	follistatin
GC20M057570_at	1522	CTSZ	cathepsin Z
GC12P086201_at	4922	NTS	neurotensin
GC01M153507_at	6277	S100A6	S100 calcium binding protein A6
GC19P038755_at	10653	SPINT2	serine peptidase inhibitor, Kunitz type, 2
GC19P056688_at	85569	GALP	galanin-like peptide
GC12P053343_at	3875	KRT18	keratin 18
GC04M048887_at	132299	OCIAD2	OCIA domain containing 2
GC19P003884_at	27231	ITGB1BP3	integrin beta 1 binding protein 3
GC16P056659_at	4493	MT1E	metallothionein 1E
GC13M054689_at	NA	ENSG00000234787	NA
GC04M042112_at	389206	BEND4	BEN domain containing 4
GC05P147238_at	117156	SCGB3A2	secretoglobin, family 3A, member 2
GC06P031796_at	3303	HSPA1A	heat shock 70kDa protein 1A
GC15P033010_at	26585	GREM1	gremlin 1
GC14P074816_at	55237	C14orf115	chromosome 14 open reading frame 115
GC0XP101380_at	140597	TCEAL2	transcription elongation factor A (SII)-like 2
GC05M095751_at	5122	PCSK1	proprotein convertase subtilisin/kexin type 1
GC19M035988_at	93099	DMKN	dermokine
GC10M043689_at	221002	RASGEF1A	RasGEF domain family, member 1A

GC02P171669_at	2571	GAD1	glutamate decarboxylase 1 (brain, 67kDa)
GC19M055644_at	7138	TNNT1	troponin T type 1 (skeletal, slow)
GC13U900722_at	440132	LOC440132	hCG1815504
GC04P188916_at	132625	ZFP42	zinc finger protein 42 homolog (mouse)
GC04P042399_at	152573	SHISA3	shisa homolog 3 (Xenopus laevis)
GC19P035739_at	51599	LSR	lipolysis stimulated lipoprotein receptor
GC01P045792_at	84842	HPDL	4-hydroxyphenylpyruvate dioxygenase-like
GC04P079403_at	306	ANXA3	annexin A3
GC16M003064_at	9074	CLDN6	claudin 6
GC05U900401_at	153469	LOC153469	hypothetical protein LOC153469
GC16P056711_at	4501	MT1X	metallothionein 1X
GC07M102453_at	222235	FBXL13	F-box and leucine-rich repeat protein 13
GC12M053290_at	3856	KRT8	keratin 8
GC16P056691_at	4494	MT1F	metallothionein 1F
GC06M138453_at	64065	PERP	PERP, TP53 apoptosis effector
GC02M085621_at	822	CAPG	capping protein (actin filament), gelsolin-like
GC02P017643_at	7447	VSNL1	visinin-like 1
GC04P088896_at	6696	SPP1	secreted phosphoprotein 1
GC12P111374_at	100131138	LOC100131138	hypothetical LOC100131138
GC08M022545_at	1960	EGR3	early growth response 3
GC04M147628_at	83894	TTC29	tetratricopeptide repeat domain 29
GC19P038794_at	64073	C19orf33	chromosome 19 open reading frame 33
GC03M012169_at	7079	TIMP4	TIMP metallopeptidase inhibitor 4
GC14P065002_at	3306	HSPA2	heat shock 70kDa protein 2
GC11M088026_at	1075	CTSC	cathepsin C
GC04M122680_at	132332	TMEM155	transmembrane protein 155
GC08M145669_at	8928	FOXH1	forkhead box H1
GC10M044865_at	6387	CXCL12	chemokine (C-X-C motif) ligand 12
GC13P096085_at	9071	CLDN10	claudin 10
GC15M065345_at	51285	RASL12	RAS-like, family 12
GC0XM101408_at	340542	BEX5	brain expressed, X-linked 5
GC01M151955_at	6281	S100A10	S100 calcium binding protein A10
GC12P006284_at	NA	NA	NA
GC11P035116_at	960	CD44	CD44 molecule (Indian blood group)
GC11M077726_at	65987	KCTD14	potassium channel tetramerisation domain containing 14
GC05P076150_at	2150	F2RL1	coagulation factor II (thrombin) receptor-like 1
GC07P128350_at	84691	FAM71F1	family with sequence similarity 71, member F1
GC0XM078615_at	9452	ITM2A	integral membrane protein 2A
GC03P062943_at	285401	LOC285401	hypothetical LOC285401
GC19M015337_at	79852	EPHX3	epoxide hydrolase 3
GC07P116164_at	857	CAV1	caveolin 1, caveolae protein, 22kDa
GC12M014657_at	79887	PLBD1	phospholipase B domain containing 1
GC19P035645_at	53827	FXYD5	FXYD domain containing ion transport regulator 5
GC10P106018_at	119391	GSTO2	glutathione S-transferase omega 2
GC16M087863_at	8140	SLC7A5	solute carrier family 7 (cationic amino acid transporter, y+ system), member 5
GC15M035080_at	70	ACTC1	actin, alpha, cardiac muscle 1
GC01P207494_at	1604	CD55	CD55 molecule, decay accelerating factor for complement (Cromer blood group)
GC05P128824_at	171019	ADAMTS19	ADAM metallopeptidase with thrombospondin type 1 motif, 19
GC08M009032_at	79660	PPP1R3B	protein phosphatase 1, regulatory (inhibitor) subunit 3B
GC19M055663_at	7137	TNNI3	troponin I type 3 (cardiac)
GC01P011752_at	374946	C1orf187	chromosome 1 open reading frame 187

GC01P114522_at	56944	OLFML3	olfactomedin-like 3
GC02P085788_at	8673	VAMP8	vesicle-associated membrane protein 8 (endobrevin)
GC01M159887_at	8407	TAGLN2	transgelin 2
GC06M031136_at	5460	POU5F1	POU class 5 homeobox 1
GC08P095653_at	54845	ESRP1	epithelial splicing regulatory protein 1
GC11M007642_at	51700	CYB5R2	cytochrome b5 reductase 2
GC01P159750_at	54935	DUSP23	dual specificity phosphatase 23
GC03P138327_at	55179	FAIM	Fas apoptotic inhibitory molecule
GC15M026788_at	2562	GABRB3	gamma-aminobutyric acid (GABA) A receptor, beta 3
GC19M038741_at	94274	PPP1R14A	protein phosphatase 1, regulatory (inhibitor) subunit 14A
GC15P032933_at	6447	SCG5	secretogranin V (7B2 protein)
GC14P059100_at	51339	DACT1	dapper, antagonist of beta-catenin, homolog 1 (Xenopus laevis)
GC16P069140_at	3038	HAS3	hyaluronan synthase 3
GC04P084457_at	84803	AGPAT9	1-acylglycerol-3-phosphate O-acyltransferase 9
GC01P027189_at	2810	SFN	stratifin
GC13P031456_at	NA	ENSG00000236094	NA
GC19P004231_at	10148	EBI3	Epstein-Barr virus induced 3
GC03M027757_at	8320	EOMES	eomesodermin
GC16M056752_at	4495	MT1G	metallothionein 1G
GC19M019366_at	404037	HAPLN4	hyaluronan and proteoglycan link protein 4
GC14P075745_at	2353	FOS	FBJ murine osteosarcoma viral oncogene homolog
GC05P113725_at	3781	KCNN2	potassium intermediate/small conductance calcium-activated channel, subfamily N, member 2
GC02P232260_at	93010	B3GNT7	UDP-GlcNAc:betaGal beta-1,3-N-acetylglucosaminyltransferase 7
GC04P187147_at	3818	KLKB1	kallikrein B, plasma (Fletcher factor) 1
GC07M131185_at	5420	PODXL	podocalyxin-like
GC14P042076_at	145581	LRFN5	leucine rich repeat and fibronectin type III domain containing 5
GC01M229567_at	58	ACTA1	actin, alpha 1, skeletal muscle
GC07M075398_at	10344	CCL26	chemokine (C-C motif) ligand 26
GC0XP049037_at	5355	PLP2	proteolipid protein 2 (colonic epithelium-enriched)
GC07M143087_at	2041	EPHA1	EPH receptor A1
GC11M087846_at	23682	RAB38	RAB38, member RAS oncogene family
GC06M169615_at	7058	THBS2	thrombospondin 2
GC18M040323_at	6014	RIT2	Ras-like without CAAX 2
GC15M053805_at	256764	WDR72	WD repeat domain 72
GC20P033758_at	10544	PROCR	protein C receptor, endothelial
GC19P046368_at	3171	FOXA3	forkhead box A3
GC01M226073_at	10637	LEFTY1	left-right determination factor 1
GC04M088394_at	8404	SPARCL1	SPARC-like 1 (hevin)
GC12M009220_at	2	A2M	alpha-2-macroglobulin
GC08P019841_at	4023	LPL	lipoprotein lipase
GC13P042031_at	28984	C13orf15	chromosome 13 open reading frame 15
GC08P040028_at	56892	C8orf4	chromosome 8 open reading frame 4
GC02M105425_at	NA	ENSG00000233639	NA
GC05M158059_at	1879	EBF1	early B-cell factor 1
GC21M048018_at	6285	S100B	S100 calcium binding protein B
GC18P049802_at	1630	DCC	deleted in colorectal carcinoma
GC12M091430_at	4060	LUM	lumican
GC12M048266_at	1280	COL2A1	collagen, type II, alpha 1
GC11M121959_at	399959	LOC399959	hypothetical LOC399959

GC04P090800_at	22915	MMRN1	multimerin 1
GC02P189803_at	1281	COL3A1	collagen, type III, alpha 1
GC17M066863_at	10351	ABCA8	ATP-binding cassette, sub-family A (ABC1), member 8
GC08P104152_at	79870	BAALC	brain and acute leukemia, cytoplasmic
GC05M136339_at	6695	SPOCK1	sparc/osteonectin, cwcv and kazal-like domains proteoglycan (testican) 1
GC11M031768_at	5080	PAX6	paired box 6
GC04M176554_at	2823	GPM6A	glycoprotein M6A
GC19M054976_at	148170	CDC42EP5	CDC42 effector protein (Rho GTPase binding) 5
GC03M165490_at	590	BCHE	butyrylcholinesterase
GC06P150920_at	57480	PLEKHG1	pleckstrin homology domain containing, family G (with RhoGef domain) member 1
GC06P123142_at	2173	FABP7	fatty acid binding protein 7, brain
GC05P092920_at	7025	NR2F1	nuclear receptor subfamily 2, group F, member 1
GC12M091472_at	1634	DCN	decorin
GC01M242251_at	200150	PLD5	phospholipase D family, member 5
GC01M062701_at	163782	KANK4	KN motif and ankyrin repeat domains 4
GC03M008637_at	51066	C3orf32	chromosome 3 open reading frame 32
GC04M157682_at	56034	PDGFC	platelet derived growth factor C
GC06P050833_at	7021	TFAP2B	transcription factor AP-2 beta (activating enhancer binding protein 2 beta)
GC18M000309_at	81035	COLEC12	collectin sub-family member 12
GC05M011024_at	1501	CTNND2	catenin (cadherin-associated protein), delta 2 (neural plakophilin-related arm-repeat protein)
GC14P063671_at	57381	RHOJ	ras homolog gene family, member J
GC07P086274_at	2913	GRM3	glutamate receptor, metabotropic 3
GC01P079085_at	10964	IFI44L	interferon-induced protein 44-like
GC08M133792_at	137835	TMEM71	transmembrane protein 71
GC02P039892_at	130733	TMEM178	transmembrane protein 178
GC12M015673_at	2059	EPS8	epidermal growth factor receptor pathway substrate 8
GC0XP152762_at	633	BGN	biglycan
GC05M146949_at	9832	JAKMIP2	janus kinase and microtubule interacting protein 2
GC05M087871_at	645323	LOC645323	hypothetical LOC645323
GC06U900288_at	285758	LOC285758	hypothetical LOC285758
GC08P054814_at	NA	NA	NA
GC03P157154_at	5806	PTX3	pentraxin 3, long
GC01M103342_at	1301	COL11A1	collagen, type XI, alpha 1
GC08P065285_at	100130155	LOC100130155	hypothetical LOC100130155
GC02M180306_at	151126	ZNF385B	zinc finger protein 385B
GC08P085095_at	138046	RALYL	RALY RNA binding protein-like
GC04P124317_at	10252	SPRY1	sprouty homolog 1, antagonist of FGF signaling (Drosophila)
GC20P020296_at	3642	INSM1	insulinoma-associated 1
GC07M016467_at	25928	SOSTDC1	sclerostin domain containing 1
GC10M048425_at	2662	GDF10	growth differentiation factor 10
GC13P058205_at	27253	PCDH17	protocadherin 17
GC06P099295_at	5454	POU3F2	POU class 3 homeobox 2
GC04M076954_at	6373	CXCL11	chemokine (C-X-C motif) ligand 11
GC07M028960_at	9865	TRIL	TLR4 interactor with leucine rich repeats
GC03M047603_at	10675	CSPG5	chondroitin sulfate proteoglycan 5 (neuroglycan C)
GC06M072119_at	79940	C6orf155	chromosome 6 open reading frame 155
GC18P047088_at	9388	LIPG	lipase, endothelial
GC08M102767_at	83988	NCALD	neurocalcin delta
GC09M038396_at	347252	IGFBPL1	insulin-like growth factor binding protein-like 1

GC02M182971_at	5136	PDE1A	phosphodiesterase 1A, calmodulin-dependent
GC04P147560_at	5458	POU4F2	POU class 4 homeobox 2
GC11P007463_at	283298	OLFML1	olfactomedin-like 1
GC01P170632_at	5396	PRRX1	paired related homeobox 1
GC05P126654_at	84466	MEGF10	multiple EGF-like-domains 10
GC08U901139_at	643763	LOC643763	hypothetical LOC643763
GC13M036048_at	4081	MAB21L1	mab-21-like 1 (C. elegans)
GC05M038515_at	3977	LIFR	leukemia inhibitory factor receptor alpha
GC18P043914_at	494470	RNF165	ring finger protein 165
GC05M092771_at	441094	FLJ42709	hypothetical LOC441094
GC07M157331_at	5799	PTPRN2	protein tyrosine phosphatase, receptor type, N polypeptide 2
GC15P096869_at	7026	NR2F2	nuclear receptor subfamily 2, group F, member 2
GC02M133429_at	344148	NCKAP5	NCK-associated protein 5
GC09M093373_at	54769	DIRAS2	DIRAS family, GTP-binding RAS-like 2
GC02M068511_at	25927	CNRIP1	cannabinoid receptor interacting protein 1
GC01M178818_at	9068	ANGPTL1	angiopoietin-like 1
GC05M100171_at	7903	ST8SIA4	ST8 alpha-N-acetyl-neuraminide alpha-2,8-sialyltransferase 4
GC12M022246_at	6489	ST8SIA1	ST8 alpha-N-acetyl-neuraminide alpha-2,8-sialyltransferase 1
GC07M015617_at	4223	MEOX2	mesenchyme homeobox 2
GC08P070428_at	23213	SULF1	sulfatase 1
GC02M192777_at	23671	TMEFF2	transmembrane protein with EGF-like and two follistatin-like domains 2
GC12P044229_at	84216	TMEM117	transmembrane protein 117
GC17M053796_at	55273	TMEM100	transmembrane protein 100
GC02P066660_at	4211	MEIS1	Meis homeobox 1
GC01M079355_at	64123	ELTD1	EGF, latrophilin and seven transmembrane domain containing 1
GC06P069404_at	577	BAI3	brain-specific angiogenesis inhibitor 3
GC16P008675_at	18	ABAT	4-aminobutyrate aminotransferase
GC01M193147_at	8707	B3GALT2	UDP-Gal:betaGlcNAc beta 1,3-galactosyltransferase, polypeptide 2
GC12P012831_at	81575	APOLD1	apolipoprotein L domain containing 1
GC02M000271_at	285016	FAM150B	family with sequence similarity 150, member B
GC0XP129305_at	9363	RAB33A	RAB33A, member RAS oncogene family
GC13M066876_at	5101	PCDH9	protocadherin 9
GC19P054926_at	57348	TTYH1	tweety homolog 1 (Drosophila)
GC07M124386_at	2861	GPR37	G protein-coupled receptor 37 (endothelin receptor type B-like)
GC07M045918_at	3486	IGFBP3	insulin-like growth factor binding protein 3
GC17P027573_at	1411	CRYBA1	crystallin, beta A1
GC04M174309_at	11341	SCRG1	stimulator of chondrogenesis 1
GC13P064321_at	647262	LOC647262	hypothetical LOC647262
GC16P055020_at	10265	IRX5	iroquois homeobox 5
GC05P140572_at	56126	PCDHB10	protocadherin beta 10
GC20M014251_at	23767	FLRT3	fibronectin leucine rich transmembrane protein 3
GC07M019121_at	7291	TWIST1	twist homolog 1 (Drosophila)
GC02M183662_at	2487	FRZB	frizzled-related protein
GC0XM110537_at	1641	DCX	doublecortin
GC21P017442_at	388815	C21orf34	chromosome 21 open reading frame 34
GC01M206238_at	440712	C1orf186	chromosome 1 open reading frame 186
GC0XM114238_at	3598	IL13RA2	interleukin 13 receptor, alpha 2
GC06M055667_at	653	BMP5	bone morphogenetic protein 5

GC14P094385_at	90050	FAM181A	family with sequence similarity 181, member A
GC12P106976_at	5992	RFX4	regulatory factor X, 4 (influences HLA class II expression)
GC12M015034_at	4256	MGP	matrix Gla protein
