# Supplementary material for: Genome-Wide Definition of Promoter and Enhancer Usage during Neural Induction of Human Embryonic Stem Cells
Source: PLoS One. 2015 May 15;10(5):e0126590. doi: 10.1371/journal.pone.0126590 (PMC4433211; doi:10.1371/journal.pone.0126590)
Supplement: S2 Table — (PDF) [file pone.0126590.s012.pdf]

TABLE S2

Table of CAGE promoters associated to ncRNAs in ESCs (red) and NESCs (blue)

CAGE_ID: univocal identification code of CAGE promoter
ESCs tpm: CAGE promoter expression value in ESCs
NESCs tpm: CAGE promoter expression value in NESCs
Gene Name (RefSeq): gene official symbol provided by HGNC (HUGO Gene Nomenclature Committee)
Mol. Status (RefSeq): type of transcript from RefSeq database, mRNA and RNA indicate respectively protein-coding and non-coding transcripts
Status (RefSeq): status of the gene provided by RefSeq database (validated, reviewed, inferred, predicted or provisional)
Gene Type (Encode): type of transcript from Gencode database

CAGE_ID	ESC tpm	NESC tpm	Gene Name (RefSeq)	Status (RefSeq)	Mol. Status (RefSeq)	Gene Type (Gencode)
L2_3244_hg19_inputLibs_chr19_-_3982310	495	408	SNORD37	Provisional	RNA	snoRNA
L2_3244_hg19_inputLibs_chr6_-_86388451	426	217	SNHG5	Validated	RNA	lincRNA
L2_3244_hg19_inputLibs_chr17_+_74553875	283	127	SNHG16	Validated	RNA	
L2_3244_hg19_inputLibs_chr4_-_113569855	280	0	MIR302B	Validated	RNA	miRNA
L2_3244_hg19_inputLibs_chr4_-_113569855	280	0	MIR302B	Validated	RNA	antisense
L2_3244_hg19_inputLibs_chr4_-_113569855	280	0	MIR302C	Validated	RNA	miRNA
L2_3244_hg19_inputLibs_chr4_-_113569855	280	0	MIR302C	Validated	RNA	antisense
L2_3244_hg19_inputLibs_chr19_-_3982833	217	153	SNORD37	Provisional	RNA	snoRNA
L2_3244_hg19_inputLibs_chr5_+_146939557	204	0	JAKMIP2-AS1	Validated	RNA	.
L2_3244_hg19_inputLibs_chr20_+_57464233	193	206	GNAS	Reviewed	RNA	.
L2_3244_hg19_inputLibs_chrX_-_130964613	165	7	LOC286467	Validated	RNA	Processed transcript
L2_3244_hg19_inputLibs_chrX_+_73164172	148	169	JPX	Validated	RNA	lincRNA
L2_3244_hg19_inputLibs_chr2_-_232325003	145	64	SNORD82	Provisional	RNA	snoRNA
L2_3244_hg19_inputLibs_chr12_+_100594592	144	154	ACTR6	Validated	RNA	
L2_3244_hg19_inputLibs_chr20_+_47895177	133	146	SNORD12C	Provisional	RNA	antisense
L2_3244_hg19_inputLibs_chr20_+_47895177	133	146	SNORD12C	Provisional	RNA	snoRNA
L2_3244_hg19_inputLibs_chr20_+_47895177	133	146	ZFAS1	Validated	RNA	antisense
L2_3244_hg19_inputLibs_chr20_+_47895177	133	146	ZFAS1	Validated	RNA	snoRNA
L2_3244_hg19_inputLibs_chr3_+_196669561	131	178	NCBP2-AS2	Validated	RNA	.

L2_3244_hg19_inputLibs_chr2_-_232325460	115	57	SNORD82	Provisional	RNA	snoRNA
L2_3244_hg19_inputLibs_chr18_+_47340378	103	142	SCARNA17	Provisional	RNA	antisense
L2_3244_hg19_inputLibs_chr18_+_47340378	103	142	SCARNA17	Provisional	RNA	snoRNA
L2_3244_hg19_inputLibs_chr15_+_41576204	93	69	OIP5-AS1	Predicted	RNA	lincRNA
L2_3244_hg19_inputLibs_chr7_+_5013717	92	65	RNF216P1	Validated	RNA	.
L2_3244_hg19_inputLibs_chr6_+_29855505	83	51	HLA-H	Reviewed	RNA	.
L2_3244_hg19_inputLibs_chr3_-_169482847	81	9	TERC	Reviewed	RNA	misc_RNA
L2_3244_hg19_inputLibs_chr12_-_58329799	80	162	LOC100506844	Validated	RNA	processed transcript
L2_3244_hg19_inputLibs_chr17_-_37009337	78	35	SNORA21	Provisional	RNA	snoRNA
L2_3244_hg19_inputLibs_chr17_-_37009337	78	35	SNORA21	Provisional	RNA	
L2_3244_hg19_inputLibs_chr5_+_111496560	78	64	EPB41L4A-AS1	Validated	RNA	lincRNA
L2_3244_hg19_inputLibs_chr9_+_33624313	77	30	ANXA2P2	Validated	RNA	.
L2_3244_hg19_inputLibs_chr12_-_123849385	73	105	MIR8072	Provisional	RNA	.
L2_3244_hg19_inputLibs_chr10_+_38645342	71	83	HSD17B7P2	Provisional	RNA	pseudogene
L2_3244_hg19_inputLibs_chr2_+_122288454	66	80	RNU4ATAC	Reviewed	RNA	snRNA
L2_3244_hg19_inputLibs_chr2_+_122288454	66	80	RNU4ATAC	Reviewed	RNA	antisense
L2_3244_hg19_inputLibs_chr14_+_96342781	65	4	LINC00617	Validated	RNA	lincRNA
L2_3244_hg19_inputLibs_chr1_-_173837129	64	10	GAS5	Provisional	RNA	processed transcript
L2_3244_hg19_inputLibs_chr1_-_173837129	64	10	GAS5	Provisional	RNA	snoRNA
L2_3244_hg19_inputLibs_chr1_-_173837129	64	10	SNORD74	Provisional	RNA	processed transcript
L2_3244_hg19_inputLibs_chr1_-_173837129	64	10	SNORD74	Provisional	RNA	snoRNA
L2_3244_hg19_inputLibs_chr15_-_66795073	64	35	SNORD16	Provisional	RNA	snoRNA
L2_3244_hg19_inputLibs_chr16_+_89778288	64	2	VPS9D1-AS1	Validated	RNA	antisense
L2_3244_hg19_inputLibs_chr7_+_27135787	64	7	HOTAIRM1	Reviewed	RNA	antisense
L2_3244_hg19_inputLibs_chr7_+_72300051	62	50	SBDSP1	Validated	RNA	.
L2_3244_hg19_inputLibs_chr19_+_23945804	58	21	RPSAP58	Validated	RNA	pseudogene
L2_3244_hg19_inputLibs_chr19_+_23945804	58	21	RPSAP58	Validated	RNA	
L2_3244_hg19_inputLibs_chr15_+_74753649	56	66	UBL7-AS1	Validated	RNA	antisense
L2_3244_hg19_inputLibs_chrX_+_56755704	52	69	LOC550643	Predicted	RNA	lincRNA
L2_3244_hg19_inputLibs_chr7_+_79083379	50	129	MAGI2-AS3	Validated	RNA	processed transcript

L2_3244_hg19_inputLibs_chr4_-_83821700	48	50	THAP9-AS1	Validated	RNA	processed transcript
L2_3244_hg19_inputLibs_chr4_-_83821700	48	50	THAP9-AS1	Validated	RNA	
L2_3244_hg19_inputLibs_chr8_-_27468022	48	5	MIR6843	Provisional	RNA	
L2_3244_hg19_inputLibs_chr19_-_36822601	46	8	LINC00665	Validated	RNA	lincRNA
L2_3244_hg19_inputLibs_chr10_-_89102097	45	60	NUTM2A-AS1	Validated	RNA	antisense
L2_3244_hg19_inputLibs_chr16_+_56651407	45	0	MT1L	Validated	RNA	pseudogene
L2_3244_hg19_inputLibs_chr1_-_8926370	44	16	MIR6728	Provisional	RNA	.
L2_3244_hg19_inputLibs_chr6_-_30294166	43	26	HCG17	Validated	RNA	lincRNA
L2_3244_hg19_inputLibs_chr19_-_23433196	42	20	ZNF724P	Provisional	RNA	
L2_3244_hg19_inputLibs_chr2_+_189157502	40	47	GULP1	Reviewed	RNA	
L2_3244_hg19_inputLibs_chr3_+_183165437	40	58	LINC00888	Validated	RNA	pseudogene
L2_3244_hg19_inputLibs_chr8_-_27469237	40	3	CLU	Reviewed	RNA	
L2_3244_hg19_inputLibs_chr1_+_109642814	39	73	SCARNA2	Provisional	RNA	snoRNA
L2_3244_hg19_inputLibs_chr1_-_28908384	38	14	SNHG12	Predicted	RNA	lincRNA
L2_3244_hg19_inputLibs_chr20_-_34638886	37	73	LINC00657	Validated	RNA	lincRNA
L2_3244_hg19_inputLibs_chr4_-_467951	37	24	ABCA11P	Validated	RNA	
L2_3244_hg19_inputLibs_chr4_-_467951	37	24	ABCA11P	Validated	RNA	pseudogene
L2_3244_hg19_inputLibs_chr8_-_146017217	37	22	MIR6850	Provisional	RNA	
L2_3244_hg19_inputLibs_chr16_-_28891207	36	26	LOC100289092	Validated	RNA	antisense
L2_3244_hg19_inputLibs_chr16_+_90039018	34	37	AFG3L1P	Validated	RNA	pseudogene
L2_3244_hg19_inputLibs_chr19_+_804554	33	25	MIR4745	Provisional	RNA	miRNA
L2_3244_hg19_inputLibs_chr8_-_67837776	33	30	SNHG6	Provisional	RNA	lincRNA
L2_3244_hg19_inputLibs_chr14_-_58764857	31	53	FLJ31306	Validated	RNA	processed transcript
L2_3244_hg19_inputLibs_chr10_-_28821672	29	34	WAC-AS1	Validated	RNA	antisense
L2_3244_hg19_inputLibs_chr10_-_89102347	29	24	NUTM2A-AS1	Validated	RNA	antisense
L2_3244_hg19_inputLibs_chr20_+_18548074	29	33	LINC00493	Predicted	RNA	
L2_3244_hg19_inputLibs_chrX_+_100740438	29	23	ARMCX4	Validated	RNA	.
L2_3244_hg19_inputLibs_chr15_+_99797970	27	12	HSP90B2P	Validated	RNA	miRNA
L2_3244_hg19_inputLibs_chr19_-_23457027	27	52	LOC100132815	Validated	RNA	.
L2_3244_hg19_inputLibs_chr2_-_232325239	27	15	SNORD82	Provisional	RNA	snoRNA

L2_3244_hg19_inputLibs_chr4_+_165798328	27	2	LOC100506013	Validated	RNA	lincRNA
L2_3244_hg19_inputLibs_chrX_-_62780763	27	22	LOC92249	Predicted	RNA	processed transcript
L2_3244_hg19_inputLibs_chr15_-_66794235	26	14	SNORD18B	Provisional	RNA	snoRNA
L2_3244_hg19_inputLibs_chr17_+_16342349	26	13	FAM211A-AS1	Predicted	RNA	processed transcript
L2_3244_hg19_inputLibs_chr17_+_16342349	26	13	FAM211A-AS1	Predicted	RNA	snoRNA
L2_3244_hg19_inputLibs_chr17_+_16342349	26	13	SNORD49B	Provisional	RNA	processed transcript
L2_3244_hg19_inputLibs_chr17_+_16342349	26	13	SNORD49B	Provisional	RNA	snoRNA
L2_3244_hg19_inputLibs_chr7_-_45808576	26	23	SEPT7P2	Provisional	RNA	pseudogene
L2_3244_hg19_inputLibs_chr7_+_99933735	26	29	STAG3L5P	Validated	RNA	
L2_3244_hg19_inputLibs_chr7_+_99933735	26	29	STAG3L5P	Validated	RNA	pseudogene
L2_3244_hg19_inputLibs_chr7_+_99933735	26	29	STAG3L5P-PVRIG2P-PILRB	Validated	RNA	pseudogene
L2_3244_hg19_inputLibs_chr8_-_27468735	26	5	CLU	Reviewed	RNA	
L2_3244_hg19_inputLibs_chr1_+_19934580	24	7	RPS14P3	Validated	RNA	.
L2_3244_hg19_inputLibs_chr13_-_54707016	24	0	LINC00458	Validated	RNA	lincRNA
L2_3244_hg19_inputLibs_chr11_+_65190267	23	41	NEAT1	Validated	RNA	lincRNA
L2_3244_hg19_inputLibs_chr18_+_5238094	23	32	LINC00667	Validated	RNA	lincRNA
L2_3244_hg19_inputLibs_chr19_-_20607762	23	0	ZNF826P	Validated	RNA	pseudogene
L2_3244_hg19_inputLibs_chr11_-_62623357	22	7	SNHG1	Provisional	RNA	snoRNA
L2_3244_hg19_inputLibs_chr11_-_62623357	22	7	SNHG1	Provisional	RNA	processed transcript
L2_3244_hg19_inputLibs_chr11_-_62623357	22	7	SNORD25	Provisional	RNA	snoRNA
L2_3244_hg19_inputLibs_chr11_-_62623357	22	7	SNORD25	Provisional	RNA	processed transcript
L2_3244_hg19_inputLibs_chr19_+_21541793	22	19	ZNF738	Validated	RNA	
L2_3244_hg19_inputLibs_chr22_+_31365253	22	37	TUG1	Validated	RNA	.
L2_3244_hg19_inputLibs_chr22_+_43011248	22	15	RNU12	Validated	RNA	snRNA
L2_3244_hg19_inputLibs_chr7_+_143104906	22	1	EPHA1-AS1	Validated	RNA	lincRNA
L2_3244_hg19_inputLibs_chr13_+_50656331	21	12	DLEU1	Provisional	RNA	
L2_3244_hg19_inputLibs_chr5_+_146939431	21	0	JAKMIP2-AS1	Validated	RNA	.
L2_3244_hg19_inputLibs_chr8_-_131028883	21	15	FAM49B	Validated	RNA	

L2_3244_hg19_inputLibs_chr10_+_102133598	20	113	LINC00263	Validated	RNA	lincRNA
L2_3244_hg19_inputLibs_chr11_+_82783116	20	41	RAB30-AS1	Validated	RNA	lincRNA
L2_3244_hg19_inputLibs_chr13_-_52768545	20	30	MRPS31P5	Validated	RNA	.
L2_3244_hg19_inputLibs_chr17_+_20771800	20	34	LOC440416	Validated	RNA	antisense
L2_3244_hg19_inputLibs_chr22_+_28315484	20	23	TTC28-AS1	Validated	RNA	.
L2_3244_hg19_inputLibs_chr6_-_28186888	20	29	TOB2P1	Validated	RNA	.
L2_3244_hg19_inputLibs_chr7_-_45026223	20	6	SNHG15	Provisional	RNA	lincRNA
L2_3244_hg19_inputLibs_chr9_+_33624238	20	16	ANXA2P2	Validated	RNA	.
L2_3244_hg19_inputLibs_chr17_-_42264074	18	29	ASB16-AS1	Validated	RNA	antisense
L2_3244_hg19_inputLibs_chr17_-_42264074	18	29	ASB16-AS1	Validated	RNA	
L2_3244_hg19_inputLibs_chr4_+_6675796	18	36	LOC93622	Predicted	RNA	.
L2_3244_hg19_inputLibs_chr18_+_32870278	17	18	ZNF271	Validated	RNA	pseudogene
L2_3244_hg19_inputLibs_chr5_-_1594592	17	13	SDHAP3	Validated	RNA	pseudogene
L2_3244_hg19_inputLibs_chr7_-_97601625	17	20	MGC72080	Provisional	RNA	.
L2_3244_hg19_inputLibs_chr8_-_145158678	17	24	SHARPIN	Validated	RNA	
L2_3244_hg19_inputLibs_chrX_-_119379075	17	13	NKAPP1	Validated	RNA	pseudogene
L2_3244_hg19_inputLibs_chr12_-_6690893	16	20	SCARNA11	Provisional	RNA	snoRNA
L2_3244_hg19_inputLibs_chr20_-_34638848	16	36	LINC00657	Validated	RNA	lincRNA
L2_3244_hg19_inputLibs_chr4_+_120376013	16	18	LOC645513	Validated	RNA	.
L2_3244_hg19_inputLibs_chr1_+_211556143	15	32	LINC00467	Validated	RNA	lincRNA
L2_3244_hg19_inputLibs_chr17_+_1420198	15	12	PITPNA-AS1	Validated	RNA	antisense
L2_3244_hg19_inputLibs_chr17_+_27717357	15	19	MIR4523	Provisional	RNA	
L2_3244_hg19_inputLibs_chr17_+_27717357	15	19	MIR4523	Provisional	RNA	miRNA
L2_3244_hg19_inputLibs_chr5_-_1634072	15	5	LOC728613	Validated	RNA	pseudogene
L2_3244_hg19_inputLibs_chr5_+_87564753	15	23	TMEM161B-AS1	Validated	RNA	lincRNA
L2_3244_hg19_inputLibs_chr8_-_146228281	15	9	ZNF252P	Validated	RNA	pseudogene
L2_3244_hg19_inputLibs_chr11_+_62649169	14	8	SLC3A2	Reviewed	RNA	
L2_3244_hg19_inputLibs_chr17_+_41277595	14	20	NBR2	Reviewed	RNA	pseudogene
L2_3244_hg19_inputLibs_chr20_-_33865906	14	22	MMP24-AS1	Validated	RNA	antisense

L2_3244_hg19_inputLibs_chr20_-_33865906	14	22	MMP24-AS1	Validated	RNA	
L2_3244_hg19_inputLibs_chr20_-_37063971	14	5	SNHG17	Predicted	RNA	.
L2_3244_hg19_inputLibs_chr22_+_22652519	14	27	BMS1P20	Reviewed	RNA	.
L2_3244_hg19_inputLibs_chr7_-_99933584	14	11	PMS2P1	Provisional	RNA	.
L2_3244_hg19_inputLibs_chr10_+_28821695	13	14	WAC	Reviewed	RNA	
L2_3244_hg19_inputLibs_chr12_-_11324208	13	32	PRH1-PRR4	Validated	RNA	sense_intronic
L2_3244_hg19_inputLibs_chr12_-_11324208	13	32	PRH1-PRR4	Validated	RNA	
L2_3244_hg19_inputLibs_chr12_-_57038993	13	5	SNORD59A	Provisional	RNA	snoRNA
L2_3244_hg19_inputLibs_chr14_+_65879527	13	10	FUT8	Reviewed	RNA	
L2_3244_hg19_inputLibs_chr2_+_3606246	13	12	RNASEH1-AS1	Validated	RNA	lincRNA
L2_3244_hg19_inputLibs_chr3_+_167967451	13	60	EGFEM1P	Validated	RNA	.
L2_3244_hg19_inputLibs_chr3_+_181328306	13	35	SOX2-OT	Validated	RNA	.
L2_3244_hg19_inputLibs_chr4_+_53578669	13	5	DANCR	Validated	RNA	lincRNA
L2_3244_hg19_inputLibs_chr4_+_53578669	13	5	DANCR	Validated	RNA	miRNA
L2_3244_hg19_inputLibs_chr4_+_53578669	13	5	MIR4449	Provisional	RNA	lincRNA
L2_3244_hg19_inputLibs_chr4_+_53578669	13	5	MIR4449	Provisional	RNA	miRNA
L2_3244_hg19_inputLibs_chr6_+_31430960	13	0	HCP5	Validated	RNA	.
L2_3244_hg19_inputLibs_chr7_+_66767644	13	16	STAG3L4	Validated	RNA	pseudogene
L2_3244_hg19_inputLibs_chr8_-_27468112	13	2	MIR6843	Provisional	RNA	
L2_3244_hg19_inputLibs_chr10_+_102133385	12	162	LINC00263	Validated	RNA	lincRNA
L2_3244_hg19_inputLibs_chr12_+_120639111	12	6	PXN-AS1	Validated	RNA	antisense
L2_3244_hg19_inputLibs_chr15_-_34875855	12	8	GOLGA8B	Validated	RNA	
L2_3244_hg19_inputLibs_chr3_+_52725020	12	5	SNORD19B	Validated	RNA	snoRNA
L2_3244_hg19_inputLibs_chr9_-_35658016	12	13	RMRP	Reviewed	RNA	misc_RNA
L2_3244_hg19_inputLibs_chr1_-_3663939	11	19	TP73-AS1	Validated	RNA	antisense
L2_3244_hg19_inputLibs_chr12_-_57037356	11	8	SNORD59B	Provisional	RNA	
L2_3244_hg19_inputLibs_chr12_-_93965544	11	5	SOCS2-AS1	Validated	RNA	lincRNA
L2_3244_hg19_inputLibs_chr12_-_9600790	11	14	DDX12P	Validated	RNA	.
L2_3244_hg19_inputLibs_chr12_-_98910269	11	33	TMPO-AS1	Validated	RNA	lincRNA

L2_3244_hg19_inputLibs_chr12_+_30948810	11	1	LINC00941	Validated	RNA	processed transcript
L2_3244_hg19_inputLibs_chr14_+_65879738	11	7	FUT8	Reviewed	RNA	
L2_3244_hg19_inputLibs_chr16_-_74402041	11	11	LOC283922	Validated	RNA	pseudogene
L2_3244_hg19_inputLibs_chr17_-_45569842	11	10	MRPL45P2	Validated	RNA	pseudogene
L2_3244_hg19_inputLibs_chr19_+_13051229	11	6	MIR6515	Provisional	RNA	.
L2_3244_hg19_inputLibs_chr19_+_53868975	11	3	ZNF525	Validated	RNA	
L2_3244_hg19_inputLibs_chr20_+_30135206	11	12	PSIMCT-1	Validated	RNA	.
L2_3244_hg19_inputLibs_chr5_+_180688254	11	27	TRIM52-AS1	Validated	RNA	antisense
L2_3244_hg19_inputLibs_chr7_+_143079462	11	3	MIR6892	Provisional	RNA	
L2_3244_hg19_inputLibs_chr7_+_45039364	11	6	CCM2	Reviewed	RNA	
L2_3244_hg19_inputLibs_chrX_-_73513385	11	6	FTX	Validated	RNA	.
L2_3244_hg19_inputLibs_chr1_-_155197231	10	46	GBAP1	Validated	RNA	.
L2_3244_hg19_inputLibs_chr12_+_132568828	10	26	EP400NL	Validated	RNA	
L2_3244_hg19_inputLibs_chr13_-_41495876	10	29	SUGT1P3	Validated	RNA	
L2_3244_hg19_inputLibs_chr13_-_41495876	10	29	TPTE2P5	Validated	RNA	
L2_3244_hg19_inputLibs_chr13_+_91999939	10	8	MIR17HG	Reviewed	RNA	processed transcript
L2_3244_hg19_inputLibs_chr15_-_34729979	10	10	GOLGA8A	Reviewed	RNA	.
L2_3244_hg19_inputLibs_chr15_-_66795025	10	3	SNORD16	Provisional	RNA	snoRNA
L2_3244_hg19_inputLibs_chr22_+_25844070	10	11	CRYBB2P1	Validated	RNA	.
L2_3244_hg19_inputLibs_chr5_-_80597337	10	13	CKMT2-AS1	Validated	RNA	processed transcript
L2_3244_hg19_inputLibs_chr6_-_26923961	10	10	GUSBP2	Validated	RNA	.
L2_3244_hg19_inputLibs_chr1_-_24104780	9	10	LOC100506963	Validated	RNA	antisense
L2_3244_hg19_inputLibs_chr1_+_1334926	9	14	LOC148413	Validated	RNA	
L2_3244_hg19_inputLibs_chr11_-_2019072	9	1	H19	Reviewed	RNA	lincRNA
L2_3244_hg19_inputLibs_chr11_-_71639505	9	3	LOC100133315	Validated	RNA	
L2_3244_hg19_inputLibs_chr12_-_6560668	9	5	CD27-AS1	Predicted	RNA	lincRNA
L2_3244_hg19_inputLibs_chr15_+_95398690	9	9	LOC440311	Validated	RNA	.
L2_3244_hg19_inputLibs_chr17_+_66097705	9	40	LINC00674	Validated	RNA	.
L2_3244_hg19_inputLibs_chr19_+_49993496	9	6	SNORD32A	Provisional	RNA	snoRNA

L2_3244_hg19_inputLibs_chr19_+_49993496	9	6	SNORD33	Provisional	RNA	snoRNA
L2_3244_hg19_inputLibs_chr2_-_91847996	9	15	LOC654342	Provisional	RNA	.
L2_3244_hg19_inputLibs_chr22_-_24059543	9	15	GUSBP11	Validated	RNA	.
L2_3244_hg19_inputLibs_chr4_+_119199911	9	5	SNHG8	Validated	RNA	.
L2_3244_hg19_inputLibs_chr7_+_65216100	9	10	CCT6P1	Provisional	RNA	pseudogene
L2_3244_hg19_inputLibs_chr7_+_7680364	9	11	RPA3-AS1	Validated	RNA	
L2_3244_hg19_inputLibs_chr1_+_22351993	8	7	LINC00339	Validated	RNA	lincRNA
L2_3244_hg19_inputLibs_chr10_-_75490248	8	6	BMS1P4	Provisional	RNA	processed transcript
L2_3244_hg19_inputLibs_chr10_-_75490248	8	6	BMS1P4	Provisional	RNA	pseudogene
L2_3244_hg19_inputLibs_chr11_-_10823319	8	14	SNORD97	Provisional	RNA	snoRNA
L2_3244_hg19_inputLibs_chr12_-_123755720	8	14	CDK2AP1	Reviewed	RNA	
L2_3244_hg19_inputLibs_chr12_-_123849762	8	10	MIR8072	Provisional	RNA	.
L2_3244_hg19_inputLibs_chr12_-_46121387	8	19	LINC00938	Validated	RNA	.
L2_3244_hg19_inputLibs_chr16_-_54963084	8	27	CRNDE	Validated	RNA	antisense
L2_3244_hg19_inputLibs_chr2_-_61372053	8	7	LOC339803	Validated	RNA	lincRNA
L2_3244_hg19_inputLibs_chr20_+_2636744	8	3	SNORD86	Validated	RNA	
L2_3244_hg19_inputLibs_chr20_+_2636744	8	3	SNORD86	Validated	RNA	snoRNA
L2_3244_hg19_inputLibs_chr21_+_46707995	8	8	LOC642852	Predicted	RNA	lincRNA
L2_3244_hg19_inputLibs_chr4_+_68567081	8	6	UBA6-AS1	Validated	RNA	lincRNA
L2_3244_hg19_inputLibs_chr6_+_21666684	8	13	CASC15	Predicted	RNA	.
L2_3244_hg19_inputLibs_chr7_-_65215921	8	14	LOC441242	Validated	RNA	.
L2_3244_hg19_inputLibs_chr11_+_65265223	7	7	MALAT1	Provisional	RNA	lincRNA
L2_3244_hg19_inputLibs_chr15_-_74220303	7	4	LOXL1-AS1	Validated	RNA	antisense
L2_3244_hg19_inputLibs_chr15_+_93426057	7	15	LOC100507217	Validated	RNA	.
L2_3244_hg19_inputLibs_chr16_-_70099844	7	5	PDXDC2P	Provisional	RNA	pseudogene
L2_3244_hg19_inputLibs_chr16_-_70099844	7	5	PDXDC2P	Provisional	RNA	processed transcript
L2_3244_hg19_inputLibs_chr16_-_70099844	7	5	PDXDC2P	Provisional	RNA	
L2_3244_hg19_inputLibs_chr19_+_13051426	7	3	MIR6515	Provisional	RNA	.
L2_3244_hg19_inputLibs_chr19_+_59086827	7	16	CENPBD1P1	Validated	RNA	pseudogene
L2_3244_hg19_inputLibs_chr2_-_114384663	7	11	RPL23AP7	Validated	RNA	pseudogene

L2_3244_hg19_inputLibs_chr2_-_232379052	7	6	LINC00471	Predicted	RNA	lincRNA
L2_3244_hg19_inputLibs_chr2_-_70314093	7	11	PCBP1-AS1	Validated	RNA	.
L2_3244_hg19_inputLibs_chr22_+_51222242	7	13	RPL23AP82	Validated	RNA	pseudogene
L2_3244_hg19_inputLibs_chr3_-_14989786	7	17	FGD5-AS1	Validated	RNA	antisense
L2_3244_hg19_inputLibs_chr3_-_197354723	7	14	LOC220729	Provisional	RNA	pseudogene
L2_3244_hg19_inputLibs_chr5_-_473207	7	9	PP7080	Validated	RNA	
L2_3244_hg19_inputLibs_chr5_+_27472409	7	23	LINC01021	Validated	RNA	lincRNA
L2_3244_hg19_inputLibs_chr5_+_43067126	7	14	LOC100506639	Validated	RNA	.
L2_3244_hg19_inputLibs_chr9_-_139622632	7	3	SNHG7	Validated	RNA	antisense
L2_3244_hg19_inputLibs_chr9_+_100000727	7	9	LOC100499484	Validated	RNA	
L2_3244_hg19_inputLibs_chr9_+_100000727	7	9	LOC100499484	Validated	RNA	processed transcript
L2_3244_hg19_inputLibs_chr9_+_100000727	7	9	LOC100499484-C9ORF174	Validated	RNA	
L2_3244_hg19_inputLibs_chr9_+_100000727	7	9	LOC100499484-C9ORF174	Validated	RNA	processed transcript
L2_3244_hg19_inputLibs_chr10_-_43048256	6	20	ZNF37BP	Validated	RNA	pseudogene
L2_3244_hg19_inputLibs_chr16_+_22448347	6	4	SMG1P1	Provisional	RNA	.
L2_3244_hg19_inputLibs_chr2_-_178257427	6	4	LOC100130691	Validated	RNA	processed transcript
L2_3244_hg19_inputLibs_chr2_-_178257427	6	4	LOC100130691	Validated	RNA	
L2_3244_hg19_inputLibs_chr2_+_179387938	6	8	TTN-AS1	Validated	RNA	antisense
L2_3244_hg19_inputLibs_chr4_-_141294557	6	4	LOC100129858	Validated	RNA	lincRNA
L2_3244_hg19_inputLibs_chr6_+_26421748	6	22	BTN2A3P	Validated	RNA	pseudogene
L2_3244_hg19_inputLibs_chr7_-_44058744	6	9	POLR2J4	Provisional	RNA	pseudogene
L2_3244_hg19_inputLibs_chr9_+_37079901	6	14	LOC100506710	Predicted	RNA	lincRNA
L2_3244_hg19_inputLibs_chrX_+_7065751	6	11	MIR4767	Provisional	RNA	miRNA
L2_3244_hg19_inputLibs_chr10_+_66585271	5	3	ANXA2P3	Provisional	RNA	.
L2_3244_hg19_inputLibs_chr11_+_75111821	5	4	SNORD15A	Validated	RNA	snoRNA
L2_3244_hg19_inputLibs_chr13_+_21872283	5	8	MIPEPP3	Validated	RNA	processed transcript
L2_3244_hg19_inputLibs_chr14_-_24423387	5	17	DHRS4-AS1	Validated	RNA	processed transcript
L2_3244_hg19_inputLibs_chr15_-_44829070	5	11	EIF3J-AS1	Validated	RNA	lincRNA
L2_3244_hg19_inputLibs_chr16_-_31581031	5	2	YBX3P1	Provisional	RNA	.

L2_3244_hg19_inputLibs_chr16_-_54962739	5	33	CRNDE	Validated	RNA	antisense
L2_3244_hg19_inputLibs_chr2_-_232320221	5	4	SNORA75	Provisional	RNA	snoRNA
L2_3244_hg19_inputLibs_chr2_+_37423637	5	6	CEBPZ-AS1	Validated	RNA	
L2_3244_hg19_inputLibs_chr2_+_74375265	5	3	BOLA3-AS1	Validated	RNA	processed transcript
L2_3244_hg19_inputLibs_chr22_-_42978044	5	5	RRP7B	Provisional	RNA	pseudogene
L2_3244_hg19_inputLibs_chr4_+_11370224	5	4	MIR572	Provisional	RNA	miRNA
L2_3244_hg19_inputLibs_chr5_-_180669273	5	4	SNORD96A	Provisional	RNA	snoRNA
L2_3244_hg19_inputLibs_chr6_+_52529227	5	14	LOC730101	Validated	RNA	.
L2_3244_hg19_inputLibs_chr7_-_112758627	5	6	LINC00998	Validated	RNA	
L2_3244_hg19_inputLibs_chr7_+_39773038	5	15	LINC00265	Validated	RNA	lincRNA
L2_3244_hg19_inputLibs_chr9_+_32552361	5	6	TOPORS-AS1	Validated	RNA	.
L2_3244_hg19_inputLibs_chr1_-_173446188	4	3	LOC100506023	Validated	RNA	.
L2_3244_hg19_inputLibs_chr1_-_714014	4	7	LOC100288069	Validated	RNA	lincRNA
L2_3244_hg19_inputLibs_chr1_-_93811379	4	7	LOC100131564	Validated	RNA	processed transcript
L2_3244_hg19_inputLibs_chr1_+_148928305	4	19	LOC645166	Validated	RNA	lincRNA
L2_3244_hg19_inputLibs_chr1_+_45242380	4	1	SNORD46	Provisional	RNA	snoRNA
L2_3244_hg19_inputLibs_chr11_-_9482202	4	8	LOC644656	Validated	RNA	.
L2_3244_hg19_inputLibs_chr11_+_75115103	4	2	SNORD15B	Provisional	RNA	snoRNA
L2_3244_hg19_inputLibs_chr12_-_6690489	4	5	SCARNA11	Provisional	RNA	snoRNA
L2_3244_hg19_inputLibs_chr16_+_31711913	4	3	CLUHP3	Predicted	RNA	.
L2_3244_hg19_inputLibs_chr17_-_36413281	4	8	LOC440434	Validated	RNA	
L2_3244_hg19_inputLibs_chr17_-_79008541	4	23	BAIAP2-AS1	Validated	RNA	lincRNA
L2_3244_hg19_inputLibs_chr2_-_87303545	4	5	LOC285074	Validated	RNA	.
L2_3244_hg19_inputLibs_chr2_+_243030833	4	4	LOC728323	Validated	RNA	pseudogene
L2_3244_hg19_inputLibs_chr20_+_2637442	4	1	SNORD56	Validated	RNA	snoRNA
L2_3244_hg19_inputLibs_chr20_+_2637442	4	1	SNORD56	Validated	RNA	processed transcript
L2_3244_hg19_inputLibs_chr20_+_2637442	4	1	SNORD57	Validated	RNA	snoRNA
L2_3244_hg19_inputLibs_chr20_+_2637442	4	1	SNORD57	Validated	RNA	processed transcript
L2_3244_hg19_inputLibs_chr3_-_120114683	4	14	MIR198	Provisional	RNA	.
L2_3244_hg19_inputLibs_chr3_-_14988965	4	7	FGD5-AS1	Validated	RNA	antisense

L2_3244_hg19_inputLibs_chr3_+_194304749	4	11	TMEM44-AS1	Validated	RNA	antisense
L2_3244_hg19_inputLibs_chr5_-_127418764	4	9	FLJ33630	Predicted	RNA	lincRNA
L2_3244_hg19_inputLibs_chr5_-_180258600	4	2	HEIH	Validated	RNA	.
L2_3244_hg19_inputLibs_chr5_-_43603211	4	12	NNT-AS1	Validated	RNA	lincRNA
L2_3244_hg19_inputLibs_chr7_-_66767405	4	6	PMS2P4	Validated	RNA	pseudogene
L2_3244_hg19_inputLibs_chr8_-_131028678	4	3	FAM49B	Validated	RNA	
L2_3244_hg19_inputLibs_chr9_-_99775878	4	8	HIATL2	Provisional	RNA	
L2_3244_hg19_inputLibs_chr1_-_167905233	3	5	MPC2	Validated	RNA	
L2_3244_hg19_inputLibs_chr1_+_113499009	3	3	SLC16A1-AS1	Validated	RNA	lincRNA
L2_3244_hg19_inputLibs_chr10_-_31607992	3	23	ZEB1-AS1	Predicted	RNA	antisense
L2_3244_hg19_inputLibs_chr11_+_77850853	3	11	KCTD21-AS1	Validated	RNA	lincRNA
L2_3244_hg19_inputLibs_chr12_-_45609805	3	2	PLEKHA8P1	Validated	RNA	pseudogene
L2_3244_hg19_inputLibs_chr13_+_45915682	3	13	TPT1-AS1	Validated	RNA	lincRNA
L2_3244_hg19_inputLibs_chr15_-_44828875	3	9	EIF3J-AS1	Validated	RNA	lincRNA
L2_3244_hg19_inputLibs_chr15_-_55790562	3	2	DYX1C1-CCPG1	Validated	RNA	
L2_3244_hg19_inputLibs_chr15_-_55790562	3	2	DYX1C1-CCPG1	Validated	RNA	processed transcript
L2_3244_hg19_inputLibs_chr16_-_29624760	3	0	SLC7A5P1	Validated	RNA	.
L2_3244_hg19_inputLibs_chr16_-_29625020	3	1	SLC7A5P1	Validated	RNA	.
L2_3244_hg19_inputLibs_chr16_-_30934590	3	8	FBXL19-AS1	Validated	RNA	antisense
L2_3244_hg19_inputLibs_chr17_-_62971686	3	12	AMZ2P1	Validated	RNA	pseudogene
L2_3244_hg19_inputLibs_chr18_+_3594445	3	4	DLGAP1-AS1	Validated	RNA	antisense
L2_3244_hg19_inputLibs_chr19_-_53445850	3	2	ZNF321P	Validated	RNA	
L2_3244_hg19_inputLibs_chr19_-_53445850	3	2	ZNF321P	Validated	RNA	pseudogene
L2_3244_hg19_inputLibs_chr2_-_74208568	3	0	DGUOK-AS1	Validated	RNA	antisense
L2_3244_hg19_inputLibs_chr20_+_42839740	3	6	OSER1-AS1	Validated	RNA	lincRNA
L2_3244_hg19_inputLibs_chr3_-_129118262	3	10	RPL32P3	Validated	RNA	pseudogene
L2_3244_hg19_inputLibs_chr3_-_14989629	3	12	FGD5-AS1	Validated	RNA	antisense
L2_3244_hg19_inputLibs_chr3_+_75721411	3	7	LINC00960	Validated	RNA	.
L2_3244_hg19_inputLibs_chr5_+_21459622	3	5	GUSBP1	Validated	RNA	pseudogene
L2_3244_hg19_inputLibs_chr5_+_9546395	3	14	SNHG18	Validated	RNA	lincRNA

L2_3244_hg19_inputLibs_chr6_-_29716815	3	2	HLA-F-AS1	Validated	RNA	processed transcript
L2_3244_hg19_inputLibs_chr7_+_1609723	3	6	PSMG3-AS1	Validated	RNA	lincRNA
L2_3244_hg19_inputLibs_chr7_+_72569084	3	11	GTF2IP1	Validated	RNA	.
L2_3244_hg19_inputLibs_chr7_+_72569084	3	11	LOC100093631	Validated	RNA	.
L2_3244_hg19_inputLibs_chr1_-_32707249	2	12	MTMR9LP	Validated	RNA	pseudogene
L2_3244_hg19_inputLibs_chr1_-_762882	2	5	LINC00115	Validated	RNA	lincRNA
L2_3244_hg19_inputLibs_chr1_+_120905991	2	2	HIST2H2BA	Provisional	RNA	processed transcript
L2_3244_hg19_inputLibs_chr11_-_126225457	2	14	ST3GAL4-AS1	Validated	RNA	.
L2_3244_hg19_inputLibs_chr12_+_9436259	2	3	LOC642846	Validated	RNA	.
L2_3244_hg19_inputLibs_chr13_+_95364967	2	37	SOX21-AS1	Validated	RNA	lincRNA
L2_3244_hg19_inputLibs_chr14_-_96000919	2	6	SNHG10	Reviewed	RNA	antisense
L2_3244_hg19_inputLibs_chr15_-_85113989	2	4	UBE2Q2P1	Provisional	RNA	pseudogene
L2_3244_hg19_inputLibs_chr15_+_85174695	2	5	SCAND2P	Validated	RNA	pseudogene
L2_3244_hg19_inputLibs_chr16_-_21513580	2	1	LOC100271836	Provisional	RNA	.
L2_3244_hg19_inputLibs_chr16_-_21531500	2	1	SLC7A5P2	Provisional	RNA	.
L2_3244_hg19_inputLibs_chr16_-_29625046	2	1	SLC7A5P1	Validated	RNA	.
L2_3244_hg19_inputLibs_chr17_-_36413352	2	6	LOC440434	Validated	RNA	
L2_3244_hg19_inputLibs_chr17_+_37081838	2	15	LINC00672	Validated	RNA	lincRNA
L2_3244_hg19_inputLibs_chr19_+_13051584	2	2	MIR6515	Provisional	RNA	.
L2_3244_hg19_inputLibs_chr19_+_14247956	2	6	LOC100507373	Validated	RNA	antisense
L2_3244_hg19_inputLibs_chr19_+_56905040	2	4	ZNF582-AS1	Validated	RNA	processed transcript
L2_3244_hg19_inputLibs_chr19_+_58514260	2	6	LOC100128398	Validated	RNA	.
L2_3244_hg19_inputLibs_chr2_+_39664581	2	25	LOC728730	Validated	RNA	processed transcript
L2_3244_hg19_inputLibs_chr22_+_21356507	2	6	THAP7-AS1	Validated	RNA	antisense
L2_3244_hg19_inputLibs_chr3_-_10068033	2	2	CIDECP	Validated	RNA	pseudogene
L2_3244_hg19_inputLibs_chr3_+_123304401	2	4	MYLK-AS1	Validated	RNA	lincRNA
L2_3244_hg19_inputLibs_chr3_+_129612701	2	3	TMCC1-AS1	Validated	RNA	.
L2_3244_hg19_inputLibs_chr4_-_775605	2	5	LOC100129917	Validated	RNA	processed transcript
L2_3244_hg19_inputLibs_chr4_+_25162324	2	8	SEPSECS-AS1	Validated	RNA	
L2_3244_hg19_inputLibs_chr5_-_180668622	2	2	SNORD96A	Provisional	RNA	snoRNA

L2_3244_hg19_inputLibs_chr5_-_77656223	2	6	LOC728769	Validated	RNA	lincRNA
L2_3244_hg19_inputLibs_chr7_-_74306657	2	2	STAG3L2	Validated	RNA	pseudogene
L2_3244_hg19_inputLibs_chr8_-_6264069	2	5	LOC100287015	Validated	RNA	lincRNA
L2_3244_hg19_inputLibs_chrX_+_102942193	2	6	MORF4L2-AS1	Validated	RNA	lincRNA
L2_3244_hg19_inputLibs_chr10_-_72977821	1	0	UNC5B-AS1	Validated	RNA	antisense
L2_3244_hg19_inputLibs_chr11_+_75115190	1	0	SNORD15B	Provisional	RNA	snoRNA
L2_3244_hg19_inputLibs_chr12_+_56223575	1	2	TMEM198B	Validated	RNA	pseudogene
L2_3244_hg19_inputLibs_chr16_-_2723414	1	4	ERVK13-1	Validated	RNA	processed transcript
L2_3244_hg19_inputLibs_chr17_-_13972776	1	4	COX10-AS1	Validated	RNA	lincRNA
L2_3244_hg19_inputLibs_chr17_-_1619504	1	1	MIR22HG	Validated	RNA	lincRNA
L2_3244_hg19_inputLibs_chr17_-_6915650	1	4	LOC100506713	Validated	RNA	
L2_3244_hg19_inputLibs_chr17_+_37824197	1	0	PNMT	Reviewed	RNA	
L2_3244_hg19_inputLibs_chr17_+_62223333	1	1	SNORA76	Provisional	RNA	lincRNA
L2_3244_hg19_inputLibs_chr17_+_62223333	1	1	SNORA76	Provisional	RNA	snoRNA
L2_3244_hg19_inputLibs_chr17_+_62223333	1	1	SNORD104	Provisional	RNA	lincRNA
L2_3244_hg19_inputLibs_chr17_+_62223333	1	1	SNORD104	Provisional	RNA	snoRNA
L2_3244_hg19_inputLibs_chr20_+_25990441	1	6	LOC100134868	Predicted	RNA	.
L2_3244_hg19_inputLibs_chr22_-_39712752	1	1	RNU86	Provisional	RNA	.
L2_3244_hg19_inputLibs_chr3_-_160233023	1	4	SCARNA7	Provisional	RNA	snoRNA
L2_3244_hg19_inputLibs_chr3_-_40494685	1	1	ENTPD3-AS1	Validated	RNA	lincRNA
L2_3244_hg19_inputLibs_chr6_+_2246044	1	2	GMDS-AS1	Validated	RNA	.
L2_3244_hg19_inputLibs_chr6_+_75994764	1	2	LOC100506804	Validated	RNA	lincRNA
L2_3244_hg19_inputLibs_chr7_-_75157451	1	2	PMS2P3	Validated	RNA	pseudogene
L2_3244_hg19_inputLibs_chr7_-_86974820	1	4	TP53TG1	Predicted	RNA	lincRNA
L2_3244_hg19_inputLibs_chr7_+_99578383	1	0	AZGP1P1	Validated	RNA	pseudogene
L2_3244_hg19_inputLibs_chr1_-_198906543	0	9	MIR181A1HG	Validated	RNA	.
L2_3244_hg19_inputLibs_chr10_+_29698484	0	1	PTCHD3P1	Provisional	RNA	antisense
L2_3244_hg19_inputLibs_chr13_-_50699677	0	3	DLEU2	Provisional	RNA	.
L2_3244_hg19_inputLibs_chr13_-_88323423	0	26	MIR4500HG	Validated	RNA	lincRNA
L2_3244_hg19_inputLibs_chr17_-_70588943	0	1	LINC00673	Validated	RNA	.

L2_3244_hg19_inputLibs_chr17_+_43325328	0	1	MAP3K14-AS1	Validated	RNA	antisense
L2_3244_hg19_inputLibs_chr2_-_105467911	0	149	LOC100506421	Validated	RNA	antisense
L2_3244_hg19_inputLibs_chr2_+_89065266	0	1	ANKRD36BP2	Predicted	RNA	pseudogene
L2_3244_hg19_inputLibs_chr22_+_17082373	0	1	TPTEP1	Provisional	RNA	.
L2_3244_hg19_inputLibs_chr5_-_87969136	0	25	LINC00461	Validated	RNA	lincRNA
L2_3244_hg19_inputLibs_chr5_+_38556988	0	11	LIFR-AS1	Validated	RNA	lincRNA
L2_3244_hg19_inputLibs_chr5_+_43041884	0	4	LOC153684	Predicted	RNA	lincRNA
L2_3244_hg19_inputLibs_chr5_+_92922809	0	16	MIR548AO	Provisional	RNA	.
L2_3244_hg19_inputLibs_chr5_+_92923087	0	12	MIR548AO	Provisional	RNA	.
L2_3244_hg19_inputLibs_chr6_-_114194512	0	42	LOC285758	Validated	RNA	.
L2_3244_hg19_inputLibs_chr6_+_32811892	0	1	TAPSAR1	Validated	RNA	processed transcript
L2_3244_hg19_inputLibs_chr6_+_32811892	0	1	TAPSAR1	Validated	RNA	
L2_3244_hg19_inputLibs_chr9_-_21559697	0	1	MIR31HG	Validated	RNA	lincRNA
