# Supplementary material for: Genome-Wide Definition of Promoter and Enhancer Usage during Neural Induction of Human Embryonic Stem Cells
Source: PLoS One. 2015 May 15;10(5):e0126590. doi: 10.1371/journal.pone.0126590 (PMC4433211; doi:10.1371/journal.pone.0126590)
Supplement: S3 Table — (PDF) [file pone.0126590.s013.pdf]

TABLE S3

Table of ESC-specific (red), NESC-specific (blue), up- (clear blue) and down-regulated (clear red) CAGE promoters

CAGE_ID: univocal identification code of CAGE promoter
Prom. type: classification of CAGE promoter on the basis of its activity level during neural commitment
RefSeq Gene Name: gene official symbol provided by HGNC (HUGO Gene Nomenclature Committee)
ESCs tpm: CAGE promoter expression value in ESCs
NESCs tpm: CAGE promoter expression value in NESCs
Mol. status: type of transcript from RefSeq, mRNA and RNA indicate respectively protein-coding and non-coding transcripts

CAGE_ID	Prom. type	RefSeq Gene Name	ESCs tpm	NESC tpm	Mol. status
L2_3244_hg19_inputLibs_chr1_-_102312541	downregulated	.	66	1	.
L2_3244_hg19_inputLibs_chr1_-_11741030	downregulated	MAD2L2	33	4	mRNA
L2_3244_hg19_inputLibs_chr1_-_151966311	downregulated	S100A10	128	9	mRNA
L2_3244_hg19_inputLibs_chr1_-_156265470	downregulated	C1orf85	107	9	mRNA
L2_3244_hg19_inputLibs_chr1_-_156675375	downregulated	CRABP2	2384	112	mRNA
L2_3244_hg19_inputLibs_chr1_-_159895297	downregulated	TAGLN2	19	2	mRNA
L2_3244_hg19_inputLibs_chr1_-_6260924	downregulated	.	120	1	.
L2_3244_hg19_inputLibs_chr1_+_110453470	downregulated	CSF1	40	2	mRNA
L2_3244_hg19_inputLibs_chr1_+_114522094	downregulated	OLFML3	117	6	mRNA
L2_3244_hg19_inputLibs_chr1_+_11751765	downregulated	DRAXIN	88	5	mRNA
L2_3244_hg19_inputLibs_chr1_+_12123356	downregulated	TNFRSF8	17	1	mRNA
L2_3244_hg19_inputLibs_chr1_+_174843559	downregulated	RABGAP1L	25	1	mRNA
L2_3244_hg19_inputLibs_chr1_+_207495020	downregulated	CD55	26	1	mRNA
L2_3244_hg19_inputLibs_chr1_+_21835917	downregulated	ALPL	38	1	mRNA
L2_3244_hg19_inputLibs_chr1_+_28261538	downregulated	SMPDL3B	24	1	mRNA
L2_3244_hg19_inputLibs_chr1_+_33207422	downregulated	KIAA1522	11	1	mRNA
L2_3244_hg19_inputLibs_chr1_+_62660532	downregulated	L1TD1	1109	1	mRNA
L2_3244_hg19_inputLibs_chr1_+_6304237	downregulated	HES3	622	35	mRNA
L2_3244_hg19_inputLibs_chr10_-_38109931	downregulated	.	15	1	.
L2_3244_hg19_inputLibs_chr10_-_96740975	downregulated	.	20	2	.
L2_3244_hg19_inputLibs_chr10_-_97050756	downregulated	PDLIM1	15	1	mRNA
L2_3244_hg19_inputLibs_chr10_+_106014176	downregulated	GSTO1	20	1	mRNA
L2_3244_hg19_inputLibs_chr11_-_6341731	downregulated	PRKCDBP	14	1	mRNA
L2_3244_hg19_inputLibs_chr11_-_65640338	downregulated	EFEMP2	16	2	mRNA
L2_3244_hg19_inputLibs_chr11_-_88070904	downregulated	CTSC	420	16	mRNA
L2_3244_hg19_inputLibs_chr11_+_10326618	downregulated	ADM	29	3	mRNA
L2_3244_hg19_inputLibs_chr11_+_1372939	downregulated	.	257	15	.
L2_3244_hg19_inputLibs_chr11_+_18416107	downregulated	LDHA	288	4	mRNA
L2_3244_hg19_inputLibs_chr11_+_20385281	downregulated	HTATIP2	40	3	mRNA
L2_3244_hg19_inputLibs_chr11_+_35160716	downregulated	CD44	120	7	mRNA
L2_3244_hg19_inputLibs_chr11_+_35211518	downregulated	.	13	1	.
L2_3244_hg19_inputLibs_chr11_+_66314372	downregulated	ACTN3	10	1	mRNA
L2_3244_hg19_inputLibs_chr11_+_68452010	downregulated	GAL	131	1	mRNA
L2_3244_hg19_inputLibs_chr12_-_114211456	downregulated	.	25	1	.
L2_3244_hg19_inputLibs_chr12_-_122014911	downregulated	.	13	1	.

L2_3244_hg19_inputLibs_chr12_-_122018131	downregulated	KDM2B	22	2	mRNA
L2_3244_hg19_inputLibs_chr12_-_14720742	downregulated	PLBD1	27	2	mRNA
L2_3244_hg19_inputLibs_chr12_-_56359826	downregulated	PMEL	55	2	mRNA
L2_3244_hg19_inputLibs_chr12_-_76477715	downregulated	.	33	1	.
L2_3244_hg19_inputLibs_chr12_-_8088782	downregulated	SLC2A3	3229	215	mRNA
L2_3244_hg19_inputLibs_chr12_-_95945257	downregulated	USP44	63	2	mRNA
L2_3244_hg19_inputLibs_chr12_+_30948810	downregulated	LINC00941	11	1	RNA
L2_3244_hg19_inputLibs_chr12_+_53342900	downregulated	KRT18	190	6	mRNA
L2_3244_hg19_inputLibs_chr12_+_56075518	downregulated	METTL7B	20	2	mRNA
L2_3244_hg19_inputLibs_chr12_+_6309564	downregulated	CD9	342	15	mRNA
L2_3244_hg19_inputLibs_chr13_-_36429804	downregulated	DCLK1	164	1	mRNA
L2_3244_hg19_inputLibs_chr13_-_36871979	downregulated	CCDC169	43	1	mRNA
L2_3244_hg19_inputLibs_chr13_-_36871979	downregulated	CCDC169-SOHLH2	43	1	mRNA
L2_3244_hg19_inputLibs_chr13_-_53313892	downregulated	LECT1	1866	1	mRNA
L2_3244_hg19_inputLibs_chr13_-_99293561	downregulated	.	20	1	.
L2_3244_hg19_inputLibs_chr13_+_96204972	downregulated	CLDN10	31	1	mRNA
L2_3244_hg19_inputLibs_chr14_-_21271006	downregulated	RNASE1	14	1	mRNA
L2_3244_hg19_inputLibs_chr14_-_23623602	downregulated	SLC7A8	26	2	mRNA
L2_3244_hg19_inputLibs_chr14_-_94595721	downregulated	IFI27L2	15	1	mRNA
L2_3244_hg19_inputLibs_chr14_+_42075940	downregulated	.	10	1	.
L2_3244_hg19_inputLibs_chr14_+_65007457	downregulated	HSPA2	35	1	mRNA
L2_3244_hg19_inputLibs_chr14_+_75745529	downregulated	FOS	42	5	mRNA
L2_3244_hg19_inputLibs_chr14_+_96342781	downregulated	LINC00617	65	4	RNA
L2_3244_hg19_inputLibs_chr15_-_27018184	downregulated	GABRB3	39	2	mRNA
L2_3244_hg19_inputLibs_chr15_-_27018890	downregulated	GABRB3	152	11	mRNA
L2_3244_hg19_inputLibs_chr15_+_74495334	downregulated	.	14	1	.
L2_3244_hg19_inputLibs_chr15_+_93785540	downregulated	.	30	2	.
L2_3244_hg19_inputLibs_chr16_-_3068184	downregulated	CLDN6	3238	1	mRNA
L2_3244_hg19_inputLibs_chr16_-_31214045	downregulated	PYCARD	11	1	mRNA
L2_3244_hg19_inputLibs_chr16_+_29911764	downregulated	ASPHD1	11	1	mRNA
L2_3244_hg19_inputLibs_chr16_+_3070397	downregulated	TNFRSF12A	15	1	mRNA
L2_3244_hg19_inputLibs_chr16_+_56716383	downregulated	MT1X	156	4	mRNA
L2_3244_hg19_inputLibs_chr16_+_58549435	downregulated	SETD6	59	4	mRNA
L2_3244_hg19_inputLibs_chr16_+_69139569	downregulated	HAS3	41	1	mRNA
L2_3244_hg19_inputLibs_chr16_+_69140123	downregulated	HAS3	11	1	mRNA
L2_3244_hg19_inputLibs_chr16_+_89778288	downregulated	VPS9D1-AS1	64	2	RNA
L2_3244_hg19_inputLibs_chr17_-_40170544	downregulated	.	42	1	.
L2_3244_hg19_inputLibs_chr17_-_40180358	downregulated	.	37	1	.
L2_3244_hg19_inputLibs_chr17_-_40575274	downregulated	PTRF	25	1	mRNA
L2_3244_hg19_inputLibs_chr17_-_42164119	downregulated	.	10	1	.
L2_3244_hg19_inputLibs_chr17_-_56494892	downregulated	RNF43	55	6	mRNA
L2_3244_hg19_inputLibs_chr17_-_56757051	downregulated	.	20	2	.
L2_3244_hg19_inputLibs_chr17_-_7197955	downregulated	YBX2	24	3	mRNA
L2_3244_hg19_inputLibs_chr17_+_47287761	downregulated	ABI3	23	1	mRNA
L2_3244_hg19_inputLibs_chr18_-_46978732	downregulated	.	26	1	.
L2_3244_hg19_inputLibs_chr18_+_2847010	downregulated	EMILIN2	59	2	mRNA
L2_3244_hg19_inputLibs_chr18_+_32073263	downregulated	DTNA	23	2	mRNA
L2_3244_hg19_inputLibs_chr18_+_3603968	downregulated	.	13	1	.
L2_3244_hg19_inputLibs_chr19_-_17346850	downregulated	.	66	8	.

L2_3244_hg19_inputLibs_chr19_-_17516427	downregulated	BST2	108	10	mRNA
L2_3244_hg19_inputLibs_chr19_-_39466376	downregulated	FBXO17	22	1	mRNA
L2_3244_hg19_inputLibs_chr19_-_45927639	downregulated	.	11	1	.
L2_3244_hg19_inputLibs_chr19_-_55667809	downregulated	.	51	1	.
L2_3244_hg19_inputLibs_chr19_-_55668155	downregulated	.	19	1	.
L2_3244_hg19_inputLibs_chr19_+_10736227	downregulated	SLC44A2	42	2	mRNA
L2_3244_hg19_inputLibs_chr19_+_18284621	downregulated	IFI30	78	1	mRNA
L2_3244_hg19_inputLibs_chr19_+_2476123	downregulated	GADD45B	95	8	mRNA
L2_3244_hg19_inputLibs_chr19_+_35645631	downregulated	FXYD5	43	1	mRNA
L2_3244_hg19_inputLibs_chr19_+_38755214	downregulated	SPINT2	205	7	mRNA
L2_3244_hg19_inputLibs_chr19_+_39787045	downregulated	IFNL1	28	2	mRNA
L2_3244_hg19_inputLibs_chr19_+_41699144	downregulated	CYP2S1	584	1	mRNA
L2_3244_hg19_inputLibs_chr19_+_46002952	downregulated	.	20	1	.
L2_3244_hg19_inputLibs_chr19_+_50031258	downregulated	RCN3	19	1	mRNA
L2_3244_hg19_inputLibs_chr2_-_154335303	downregulated	RPRM	21	2	mRNA
L2_3244_hg19_inputLibs_chr2_-_188378380	downregulated	.	1487	77	.
L2_3244_hg19_inputLibs_chr2_-_201967701	downregulated	.	551	68	.
L2_3244_hg19_inputLibs_chr2_-_237416101	downregulated	IQCA1	30	3	mRNA
L2_3244_hg19_inputLibs_chr2_-_40006401	downregulated	THUMPD2	10	1	mRNA
L2_3244_hg19_inputLibs_chr2_-_70995357	downregulated	ADD2	31	3	mRNA
L2_3244_hg19_inputLibs_chr2_-_74669025	downregulated	RTKN	25	2	mRNA
L2_3244_hg19_inputLibs_chr2_+_118535754	downregulated	.	19	2	.
L2_3244_hg19_inputLibs_chr2_+_37571857	downregulated	QPCT	13	1	mRNA
L2_3244_hg19_inputLibs_chr2_+_47596448	downregulated	EPCAM	53	1	mRNA
L2_3244_hg19_inputLibs_chr2_+_58273922	downregulated	VRK2	29	3	mRNA
L2_3244_hg19_inputLibs_chr2_+_64069028	downregulated	UGP2	254	16	mRNA
L2_3244_hg19_inputLibs_chr2_+_85804672	downregulated	VAMP8	36	1	mRNA
L2_3244_hg19_inputLibs_chr20_-_45947797	downregulated	.	17	2	.
L2_3244_hg19_inputLibs_chr20_-_56285071	downregulated	PMEPA1	20	2	mRNA
L2_3244_hg19_inputLibs_chr20_-_590953	downregulated	TCF15	36	1	mRNA
L2_3244_hg19_inputLibs_chr20_+_30193090	downregulated	ID1	460	48	mRNA
L2_3244_hg19_inputLibs_chr20_+_31350189	downregulated	DNMT3B	138	7	mRNA
L2_3244_hg19_inputLibs_chr20_+_33759862	downregulated	PROCR	20	1	mRNA
L2_3244_hg19_inputLibs_chr20_+_44637545	downregulated	MMP9	79	2	mRNA
L2_3244_hg19_inputLibs_chr21_-_18463299	downregulated	.	20	2	.
L2_3244_hg19_inputLibs_chr21_-_33975511	downregulated	.	14	1	.
L2_3244_hg19_inputLibs_chr22_-_18923806	downregulated	PRODH	96	3	mRNA
L2_3244_hg19_inputLibs_chr22_+_29876205	downregulated	NEFH	107	11	mRNA
L2_3244_hg19_inputLibs_chr3_-_12200375	downregulated	.	44	1	.
L2_3244_hg19_inputLibs_chr3_-_139258398	downregulated	RBP1	65	5	mRNA
L2_3244_hg19_inputLibs_chr3_-_169482847	downregulated	TERC	81	9	RNA
L2_3244_hg19_inputLibs_chr3_-_191618594	downregulated	.	44	1	.
L2_3244_hg19_inputLibs_chr3_-_48481474	downregulated	CCDC51	17	2	mRNA
L2_3244_hg19_inputLibs_chr3_-_49459891	downregulated	AMT	34	3	mRNA
L2_3244_hg19_inputLibs_chr3_+_138327611	downregulated	FAIM	28	1	mRNA
L2_3244_hg19_inputLibs_chr3_+_32859599	downregulated	TRIM71	24	1	mRNA
L2_3244_hg19_inputLibs_chr3_+_50306601	downregulated	.	19	1	.
L2_3244_hg19_inputLibs_chr4_-_1107356	downregulated	RNF212	11	1	mRNA
L2_3244_hg19_inputLibs_chr4_-_139144474	downregulated	.	108	8	.

L2_3244_hg19_inputLibs_chr4_-_186696536	downregulated	.	13	1	.
L2_3244_hg19_inputLibs_chr4_-_48908791	downregulated	OCIAD2	188	3	mRNA
L2_3244_hg19_inputLibs_chr4_-_74864394	downregulated	CXCL5	250	5	mRNA
L2_3244_hg19_inputLibs_chr4_+_113571609	downregulated	.	25	1	.
L2_3244_hg19_inputLibs_chr4_+_165798328	downregulated	LOC100506013	27	2	RNA
L2_3244_hg19_inputLibs_chr4_+_42399503	downregulated	SHISA3	24	1	mRNA
L2_3244_hg19_inputLibs_chr4_+_57458619	downregulated	.	82	9	.
L2_3244_hg19_inputLibs_chr4_+_88896864	downregulated	SPP1	349	15	mRNA
L2_3244_hg19_inputLibs_chr5_-_146833255	downregulated	DPYSL3	45	4	mRNA
L2_3244_hg19_inputLibs_chr5_-_16738340	downregulated	.	82	4	.
L2_3244_hg19_inputLibs_chr5_+_128795959	downregulated	ADAMTS19	64	1	mRNA
L2_3244_hg19_inputLibs_chr5_+_133861358	downregulated	.	19	1	.
L2_3244_hg19_inputLibs_chr5_+_137801167	downregulated	EGR1	681	72	mRNA
L2_3244_hg19_inputLibs_chr5_+_140864614	downregulated	PCDHGC4	12	1	mRNA
L2_3244_hg19_inputLibs_chr5_+_149109899	downregulated	PPARGC1B	17	1	mRNA
L2_3244_hg19_inputLibs_chr5_+_150158324	downregulated	.	10	1	.
L2_3244_hg19_inputLibs_chr5_+_176513881	downregulated	FGFR4	19	2	mRNA
L2_3244_hg19_inputLibs_chr5_+_177540542	downregulated	N4BP3	19	2	mRNA
L2_3244_hg19_inputLibs_chr5_+_52776277	downregulated	FST	1312	14	mRNA
L2_3244_hg19_inputLibs_chr5_+_52781988	downregulated	.	65	1	.
L2_3244_hg19_inputLibs_chr5_+_68788599	downregulated	OCLN	12	1	mRNA
L2_3244_hg19_inputLibs_chr5_+_76114854	downregulated	F2RL1	140	6	mRNA
L2_3244_hg19_inputLibs_chr6_-_117086886	downregulated	FAM162B	27	1	mRNA
L2_3244_hg19_inputLibs_chr6_-_138428587	downregulated	PERP	123	11	mRNA
L2_3244_hg19_inputLibs_chr6_-_32920872	downregulated	HLA-DMA	17	1	mRNA
L2_3244_hg19_inputLibs_chr6_-_74229845	downregulated	.	66	6	.
L2_3244_hg19_inputLibs_chr6_-_84140764	downregulated	ME1	39	3	mRNA
L2_3244_hg19_inputLibs_chr6_+_121756790	downregulated	GJA1	436	51	mRNA
L2_3244_hg19_inputLibs_chr6_+_121767998	downregulated	.	19	1	.
L2_3244_hg19_inputLibs_chr6_+_167641768	downregulated	.	200	2	.
L2_3244_hg19_inputLibs_chr6_+_26240619	downregulated	HIST1H4F	153	2	mRNA
L2_3244_hg19_inputLibs_chr6_+_28303527	downregulated	.	25	3	.
L2_3244_hg19_inputLibs_chr6_+_31783318	downregulated	HSPA1A	12	1	mRNA
L2_3244_hg19_inputLibs_chr7_-_100808850	downregulated	VGF	11	1	mRNA
L2_3244_hg19_inputLibs_chr7_-_131188958	downregulated	.	15	1	.
L2_3244_hg19_inputLibs_chr7_-_131196052	downregulated	.	27	1	.
L2_3244_hg19_inputLibs_chr7_-_131241387	downregulated	PODXL	4461	188	mRNA
L2_3244_hg19_inputLibs_chr7_-_558811	downregulated	.	46	4	.
L2_3244_hg19_inputLibs_chr7_-_559486	downregulated	PDGFA	22	1	mRNA
L2_3244_hg19_inputLibs_chr7_-_98030402	downregulated	BAIAP2L1	20	2	mRNA
L2_3244_hg19_inputLibs_chr7_+_100609336	downregulated	.	24	1	.
L2_3244_hg19_inputLibs_chr7_+_116165088	downregulated	CAV1	62	2	mRNA
L2_3244_hg19_inputLibs_chr7_+_116166414	downregulated	CAV1	46	5	mRNA
L2_3244_hg19_inputLibs_chr7_+_130126181	downregulated	MEST	646	3	mRNA
L2_3244_hg19_inputLibs_chr7_+_143104906	downregulated	EPHA1-AS1	22	1	RNA
L2_3244_hg19_inputLibs_chr7_+_148036587	downregulated	.	68	6	.
L2_3244_hg19_inputLibs_chr7_+_27135787	downregulated	HOTAIRM1	64	7	RNA
L2_3244_hg19_inputLibs_chr7_+_70596691	downregulated	.	19	1	.
L2_3244_hg19_inputLibs_chr7_+_70597128	downregulated	WBSCR17	22	1	mRNA

L2_3244_hg19_inputLibs_chr8_-_144816265	downregulated	FAM83H	28	1	mRNA
L2_3244_hg19_inputLibs_chr8_-_145638973	downregulated	SLC39A4	51	4	mRNA
L2_3244_hg19_inputLibs_chr8_-_27468022	downregulated	MIR6843	48	5	RNA
L2_3244_hg19_inputLibs_chr8_-_27469237	downregulated	CLU	40	3	RNA
L2_3244_hg19_inputLibs_chr8_-_9009085	downregulated	PPP1R3B	175	5	mRNA
L2_3244_hg19_inputLibs_chr8_-_91657925	downregulated	TMEM64	33	4	mRNA
L2_3244_hg19_inputLibs_chr8_+_126442610	downregulated	TRIB1	125	8	mRNA
L2_3244_hg19_inputLibs_chr8_+_87515642	downregulated	.	159	13	.
L2_3244_hg19_inputLibs_chr9_-_100881494	downregulated	TRIM14	24	2	mRNA
L2_3244_hg19_inputLibs_chr9_-_117880878	downregulated	TNC	44	4	mRNA
L2_3244_hg19_inputLibs_chr9_-_127533568	downregulated	NR6A1	37	4	mRNA
L2_3244_hg19_inputLibs_chr9_-_79307113	downregulated	.	13	1	.
L2_3244_hg19_inputLibs_chr9_-_86594496	downregulated	.	33	4	.
L2_3244_hg19_inputLibs_chr9_+_34990288	downregulated	DNAJB5	48	1	mRNA
L2_3244_hg19_inputLibs_chr9_+_75766779	downregulated	ANXA1	697	85	mRNA
L2_3244_hg19_inputLibs_chrX_-_101410954	downregulated	BEX5	17	2	mRNA
L2_3244_hg19_inputLibs_chrX_-_130423274	downregulated	IGSF1	108	1	mRNA
L2_3244_hg19_inputLibs_chrX_-_130964613	downregulated	LOC286467	165	7	RNA
L2_3244_hg19_inputLibs_chrX_-_24665447	downregulated	PCYT1B	78	6	mRNA
L2_3244_hg19_inputLibs_chrX_-_2882364	downregulated	ARSE	86	7	mRNA
L2_3244_hg19_inputLibs_chrX_-_7895767	downregulated	PNPLA4	13	1	mRNA
L2_3244_hg19_inputLibs_chrX_-_8700200	downregulated	KAL1	32	4	mRNA
L2_3244_hg19_inputLibs_chrX_+_131157300	downregulated	MST4	12	1	mRNA
L2_3244_hg19_inputLibs_chrX_+_136648569	downregulated	ZIC3	18	2	mRNA
L2_3244_hg19_inputLibs_chrX_+_150161938	downregulated	.	15	1	.
L2_3244_hg19_inputLibs_chrX_+_43515518	downregulated	.	13	1	.
L2_3244_hg19_inputLibs_chrX_+_96565605	downregulated	.	341	15	.
L2_3244_hg19_inputLibs_chr1_-_153521734	ESC-spec	S100A3	18	0	mRNA
L2_3244_hg19_inputLibs_chr1_-_161008766	ESC-spec	TSTD1	179	0	mRNA
L2_3244_hg19_inputLibs_chr1_-_1711482	ESC-spec	NADK	17	0	mRNA
L2_3244_hg19_inputLibs_chr1_-_180890263	ESC-spec	.	23	0	.
L2_3244_hg19_inputLibs_chr1_-_223202454	ESC-spec	.	35	0	.
L2_3244_hg19_inputLibs_chr1_-_226076846	ESC-spec	LEFTY1	13	0	mRNA
L2_3244_hg19_inputLibs_chr1_-_36184696	ESC-spec	C1orf216	8	0	mRNA
L2_3244_hg19_inputLibs_chr1_-_43205904	ESC-spec	CLDN19	10	0	mRNA
L2_3244_hg19_inputLibs_chr1_-_47079454	ESC-spec	.	10	0	.
L2_3244_hg19_inputLibs_chr1_-_6260658	ESC-spec	.	15	0	.
L2_3244_hg19_inputLibs_chr1_-_79959495	ESC-spec	.	24	0	.
L2_3244_hg19_inputLibs_chr1_+_145562313	ESC-spec	.	15	0	.
L2_3244_hg19_inputLibs_chr1_+_156031000	ESC-spec	RAB25	11	0	mRNA
L2_3244_hg19_inputLibs_chr1_+_183155397	ESC-spec	LAMC2	8	0	mRNA
L2_3244_hg19_inputLibs_chr1_+_18807491	ESC-spec	KLHDC7A	14	0	mRNA
L2_3244_hg19_inputLibs_chr1_+_200008082	ESC-spec	.	14	0	.
L2_3244_hg19_inputLibs_chr1_+_20915561	ESC-spec	CDA	18	0	mRNA
L2_3244_hg19_inputLibs_chr1_+_209602166	ESC-spec	MIR205HG	20	0	RNA
L2_3244_hg19_inputLibs_chr1_+_243651064	ESC-spec	.	24	0	.
L2_3244_hg19_inputLibs_chr1_+_26503996	ESC-spec	CNKSR1	9	0	mRNA
L2_3244_hg19_inputLibs_chr1_+_27189638	ESC-spec	SFN	24	0	mRNA
L2_3244_hg19_inputLibs_chr1_+_29063131	ESC-spec	YTHDF2	12	0	mRNA

L2_3244_hg19_inputLibs_chr1_+_39624534	ESC-spec	.	20	0	.
L2_3244_hg19_inputLibs_chr1_+_62661957	ESC-spec	.	27	0	.
L2_3244_hg19_inputLibs_chr1_+_62662114	ESC-spec	.	24	0	.
L2_3244_hg19_inputLibs_chr1_+_67773558	ESC-spec	.	8	0	.
L2_3244_hg19_inputLibs_chr1_+_70694169	ESC-spec	.	10	0	.
L2_3244_hg19_inputLibs_chr1_+_81711348	ESC-spec	.	10	0	.
L2_3244_hg19_inputLibs_chr1_+_95125812	ESC-spec	.	86	0	.
L2_3244_hg19_inputLibs_chr10_-_126138607	ESC-spec	NKX1-2	17	0	mRNA
L2_3244_hg19_inputLibs_chr10_-_3040372	ESC-spec	.	294	0	.
L2_3244_hg19_inputLibs_chr10_-_58982437	ESC-spec	.	140	0	.
L2_3244_hg19_inputLibs_chr10_+_106013929	ESC-spec	GSTO1	24	0	mRNA
L2_3244_hg19_inputLibs_chr10_+_135043718	ESC-spec	UTF1	19	0	mRNA
L2_3244_hg19_inputLibs_chr10_+_24544308	ESC-spec	.	11	0	.
L2_3244_hg19_inputLibs_chr10_+_82654646	ESC-spec	.	15	0	.
L2_3244_hg19_inputLibs_chr11_-_68780714	ESC-spec	MRGPRF	12	0	mRNA
L2_3244_hg19_inputLibs_chr11_-_69903591	ESC-spec	.	35	0	.
L2_3244_hg19_inputLibs_chr11_-_7695480	ESC-spec	.	9	0	.
L2_3244_hg19_inputLibs_chr11_-_77734259	ESC-spec	KCTD14	31	0	mRNA
L2_3244_hg19_inputLibs_chr11_+_125074231	ESC-spec	.	27	0	.
L2_3244_hg19_inputLibs_chr11_+_131123301	ESC-spec	.	22	0	.
L2_3244_hg19_inputLibs_chr11_+_14462294	ESC-spec	.	29	0	.
L2_3244_hg19_inputLibs_chr11_+_19799359	ESC-spec	.	11	0	.
L2_3244_hg19_inputLibs_chr11_+_35198199	ESC-spec	.	7	0	.
L2_3244_hg19_inputLibs_chr11_+_63656053	ESC-spec	MARK2	7	0	mRNA
L2_3244_hg19_inputLibs_chr11_+_94383516	ESC-spec	.	33	0	.
L2_3244_hg19_inputLibs_chr12_-_122231559	ESC-spec	RHOF	8	0	mRNA
L2_3244_hg19_inputLibs_chr12_-_49319303	ESC-spec	FKBP11	11	0	mRNA
L2_3244_hg19_inputLibs_chr12_-_54070577	ESC-spec	ATP5G2	11	0	mRNA
L2_3244_hg19_inputLibs_chr12_-_6484616	ESC-spec	SCNN1A	16	0	mRNA
L2_3244_hg19_inputLibs_chr12_+_1739191	ESC-spec	.	15	0	.
L2_3244_hg19_inputLibs_chr12_+_5153043	ESC-spec	KCNA5	11	0	mRNA
L2_3244_hg19_inputLibs_chr12_+_52445224	ESC-spec	NR4A1	16	0	mRNA
L2_3244_hg19_inputLibs_chr12_+_53343140	ESC-spec	KRT18	15	0	mRNA
L2_3244_hg19_inputLibs_chr12_+_75760716	ESC-spec	.	37	0	.
L2_3244_hg19_inputLibs_chr12_+_78334034	ESC-spec	.	22	0	.
L2_3244_hg19_inputLibs_chr12_+_7941993	ESC-spec	NANOG	53	0	mRNA
L2_3244_hg19_inputLibs_chr12_+_79933972	ESC-spec	.	36	0	.
L2_3244_hg19_inputLibs_chr12_+_88226909	ESC-spec	.	17	0	.
L2_3244_hg19_inputLibs_chr12_+_93966465	ESC-spec	SOCS2	14	0	mRNA
L2_3244_hg19_inputLibs_chr13_-_29069265	ESC-spec	FLT1	90	0	mRNA
L2_3244_hg19_inputLibs_chr13_-_53313288	ESC-spec	.	56	0	.
L2_3244_hg19_inputLibs_chr13_-_53313721	ESC-spec	LECT1	38	0	mRNA
L2_3244_hg19_inputLibs_chr13_-_54707016	ESC-spec	LINC00458	24	0	RNA
L2_3244_hg19_inputLibs_chr14_-_105436820	ESC-spec	.	46	0	.
L2_3244_hg19_inputLibs_chr14_-_21175099	ESC-spec	.	21	0	.
L2_3244_hg19_inputLibs_chr14_-_57272385	ESC-spec	OTX2	45	0	mRNA
L2_3244_hg19_inputLibs_chr14_-_99453972	ESC-spec	.	66	0	.
L2_3244_hg19_inputLibs_chr14_+_64653066	ESC-spec	.	14	0	.
L2_3244_hg19_inputLibs_chr14_+_74815210	ESC-spec	VRTN	209	0	mRNA

L2_3244_hg19_inputLibs_chr14_+_75746744	ESC-spec	.	7	0	.
L2_3244_hg19_inputLibs_chr15_-_35087949	ESC-spec	ACTC1	10	0	mRNA
L2_3244_hg19_inputLibs_chr15_+_41136244	ESC-spec	SPINT1	8	0	mRNA
L2_3244_hg19_inputLibs_chr16_-_29888774	ESC-spec	.	20	0	.
L2_3244_hg19_inputLibs_chr16_-_3149272	ESC-spec	ZSCAN10	26	0	mRNA
L2_3244_hg19_inputLibs_chr16_-_56701977	ESC-spec	MT1G	118	0	mRNA
L2_3244_hg19_inputLibs_chr16_+_56651407	ESC-spec	MT1L	45	0	RNA
L2_3244_hg19_inputLibs_chr16_+_56659694	ESC-spec	MT1E	363	0	mRNA
L2_3244_hg19_inputLibs_chr16_+_56666572	ESC-spec	MT1M	18	0	mRNA
L2_3244_hg19_inputLibs_chr16_+_56691901	ESC-spec	MT1F	153	0	mRNA
L2_3244_hg19_inputLibs_chr16_+_68679238	ESC-spec	.	15	0	.
L2_3244_hg19_inputLibs_chr16_+_68771193	ESC-spec	CDH1	53	0	mRNA
L2_3244_hg19_inputLibs_chr16_+_9768161	ESC-spec	.	17	0	.
L2_3244_hg19_inputLibs_chr17_-_1395992	ESC-spec	MYO1C	22	0	mRNA
L2_3244_hg19_inputLibs_chr17_-_27531563	ESC-spec	.	18	0	.
L2_3244_hg19_inputLibs_chr17_-_39684560	ESC-spec	KRT19	20	0	mRNA
L2_3244_hg19_inputLibs_chr17_-_39780827	ESC-spec	KRT17	14	0	mRNA
L2_3244_hg19_inputLibs_chr17_-_46608363	ESC-spec	HOXB1	22	0	mRNA
L2_3244_hg19_inputLibs_chr17_-_62009691	ESC-spec	CD79B	9	0	mRNA
L2_3244_hg19_inputLibs_chr17_-_6946309	ESC-spec	.	12	0	.
L2_3244_hg19_inputLibs_chr17_-_6980506	ESC-spec	.	24	0	.
L2_3244_hg19_inputLibs_chr17_-_7166532	ESC-spec	CLDN7	7	0	mRNA
L2_3244_hg19_inputLibs_chr17_+_27047288	ESC-spec	RPL23A	11	0	mRNA
L2_3244_hg19_inputLibs_chr17_+_43080710	ESC-spec	.	17	0	.
L2_3244_hg19_inputLibs_chr17_+_48351853	ESC-spec	TMEM92	42	0	mRNA
L2_3244_hg19_inputLibs_chr17_+_7348411	ESC-spec	CHRNB1	16	0	mRNA
L2_3244_hg19_inputLibs_chr18_-_57364654	ESC-spec	CCBE1	13	0	mRNA
L2_3244_hg19_inputLibs_chr18_+_76740143	ESC-spec	SALL3	12	0	mRNA
L2_3244_hg19_inputLibs_chr19_-_15343231	ESC-spec	EPHX3	24	0	mRNA
L2_3244_hg19_inputLibs_chr19_-_15343763	ESC-spec	EPHX3	28	0	mRNA
L2_3244_hg19_inputLibs_chr19_-_19372514	ESC-spec	.	23	0	.
L2_3244_hg19_inputLibs_chr19_-_20607762	ESC-spec	ZNF826P	23	0	RNA
L2_3244_hg19_inputLibs_chr19_-_36001345	ESC-spec	DMKN	87	0	mRNA
L2_3244_hg19_inputLibs_chr19_-_36247927	ESC-spec	HSPB6	9	0	mRNA
L2_3244_hg19_inputLibs_chr19_-_42573677	ESC-spec	.	10	0	.
L2_3244_hg19_inputLibs_chr19_-_55658322	ESC-spec	.	13	0	.
L2_3244_hg19_inputLibs_chr19_-_55658657	ESC-spec	.	19	0	.
L2_3244_hg19_inputLibs_chr19_-_55660574	ESC-spec	TNNT1	27	0	mRNA
L2_3244_hg19_inputLibs_chr19_+_11649664	ESC-spec	CNN1	20	0	mRNA
L2_3244_hg19_inputLibs_chr19_+_17889267	ESC-spec	.	10	0	.
L2_3244_hg19_inputLibs_chr19_+_35646115	ESC-spec	FXYD5	30	0	mRNA
L2_3244_hg19_inputLibs_chr19_+_35739659	ESC-spec	LSR	17	0	mRNA
L2_3244_hg19_inputLibs_chr19_+_35739909	ESC-spec	LSR	61	0	mRNA
L2_3244_hg19_inputLibs_chr19_+_3933584	ESC-spec	.	14	0	.
L2_3244_hg19_inputLibs_chr19_+_48902349	ESC-spec	.	14	0	.
L2_3244_hg19_inputLibs_chr19_+_49714002	ESC-spec	.	11	0	.
L2_3244_hg19_inputLibs_chr19_+_50305801	ESC-spec	.	20	0	.
L2_3244_hg19_inputLibs_chr19_+_7701997	ESC-spec	STXBP2	18	0	mRNA
L2_3244_hg19_inputLibs_chr2_-_220118640	ESC-spec	TUBA4A	15	0	mRNA

L2_3244_hg19_inputLibs_chr2_-_31360963	ESC-spec	.	7	0	.
L2_3244_hg19_inputLibs_chr2_-_85641139	ESC-spec	CAPG	11	0	mRNA
L2_3244_hg19_inputLibs_chr2_+_109237705	ESC-spec	LIMS1	29	0	mRNA
L2_3244_hg19_inputLibs_chr2_+_119981414	ESC-spec	STEAP3	8	0	mRNA
L2_3244_hg19_inputLibs_chr2_+_171571813	ESC-spec	SP5	39	0	mRNA
L2_3244_hg19_inputLibs_chr2_+_171572419	ESC-spec	.	44	0	.
L2_3244_hg19_inputLibs_chr2_+_171572706	ESC-spec	.	29	0	.
L2_3244_hg19_inputLibs_chr2_+_171627523	ESC-spec	.	12	0	.
L2_3244_hg19_inputLibs_chr2_+_17721971	ESC-spec	VSNL1	7	0	mRNA
L2_3244_hg19_inputLibs_chr2_+_200113942	ESC-spec	.	11	0	.
L2_3244_hg19_inputLibs_chr2_+_202047862	ESC-spec	CASP10	9	0	mRNA
L2_3244_hg19_inputLibs_chr2_+_202098184	ESC-spec	CASP8	21	0	mRNA
L2_3244_hg19_inputLibs_chr2_+_232260419	ESC-spec	B3GNT7	29	0	mRNA
L2_3244_hg19_inputLibs_chr2_+_27665246	ESC-spec	KRTCAP3	7	0	mRNA
L2_3244_hg19_inputLibs_chr2_+_47596626	ESC-spec	EPCAM	20	0	mRNA
L2_3244_hg19_inputLibs_chr2_+_47601018	ESC-spec	.	10	0	.
L2_3244_hg19_inputLibs_chr20_-_18393889	ESC-spec	.	12	0	.
L2_3244_hg19_inputLibs_chr20_-_45997873	ESC-spec	.	97	0	.
L2_3244_hg19_inputLibs_chr20_-_57582361	ESC-spec	CTSZ	33	0	mRNA
L2_3244_hg19_inputLibs_chr20_+_1710328	ESC-spec	.	12	0	.
L2_3244_hg19_inputLibs_chr20_+_44395070	ESC-spec	.	10	0	.
L2_3244_hg19_inputLibs_chr20_+_55204360	ESC-spec	TFAP2C	22	0	mRNA
L2_3244_hg19_inputLibs_chr20_+_57267952	ESC-spec	NPEPL1	10	0	mRNA
L2_3244_hg19_inputLibs_chr21_+_40823821	ESC-spec	SH3BGR	19	0	mRNA
L2_3244_hg19_inputLibs_chr22_-_19868716	ESC-spec	.	13	0	.
L2_3244_hg19_inputLibs_chr22_-_25091986	ESC-spec	.	11	0	.
L2_3244_hg19_inputLibs_chr22_-_37640299	ESC-spec	RAC2	12	0	mRNA
L2_3244_hg19_inputLibs_chr22_+_45148436	ESC-spec	ARHGAP8	25	0	mRNA
L2_3244_hg19_inputLibs_chr3_-_147309354	ESC-spec	.	88	0	.
L2_3244_hg19_inputLibs_chr3_-_184971837	ESC-spec	EHHADH	7	0	mRNA
L2_3244_hg19_inputLibs_chr3_-_21235329	ESC-spec	.	11	0	.
L2_3244_hg19_inputLibs_chr3_-_27764198	ESC-spec	EOMES	33	0	mRNA
L2_3244_hg19_inputLibs_chr3_-_45187870	ESC-spec	CDCP1	22	0	mRNA
L2_3244_hg19_inputLibs_chr3_-_48632762	ESC-spec	COL7A1	9	0	mRNA
L2_3244_hg19_inputLibs_chr3_+_115509946	ESC-spec	.	13	0	.
L2_3244_hg19_inputLibs_chr3_+_162430589	ESC-spec	.	11	0	.
L2_3244_hg19_inputLibs_chr3_+_189862813	ESC-spec	.	95	0	.
L2_3244_hg19_inputLibs_chr3_+_190638329	ESC-spec	.	18	0	.
L2_3244_hg19_inputLibs_chr3_+_38141428	ESC-spec	.	19	0	.
L2_3244_hg19_inputLibs_chr3_+_42201683	ESC-spec	TRAK1	15	0	mRNA
L2_3244_hg19_inputLibs_chr3_+_46619276	ESC-spec	TDGF1	38	0	mRNA
L2_3244_hg19_inputLibs_chr3_+_62936057	ESC-spec	.	32	0	.
L2_3244_hg19_inputLibs_chr4_-_113569855	ESC-spec	MIR302B	280	0	RNA
L2_3244_hg19_inputLibs_chr4_-_113569855	ESC-spec	MIR302C	280	0	RNA
L2_3244_hg19_inputLibs_chr4_-_122686485	ESC-spec	TMEM155	11	0	mRNA
L2_3244_hg19_inputLibs_chr4_-_168362334	ESC-spec	.	27	0	.
L2_3244_hg19_inputLibs_chr4_-_175443639	ESC-spec	.	7	0	.
L2_3244_hg19_inputLibs_chr4_-_40632869	ESC-spec	.	9	0	.
L2_3244_hg19_inputLibs_chr4_-_42294656	ESC-spec	.	31	0	.

L2_3244_hg19_inputLibs_chr4_-_62635365	ESC-spec	.	18	0	.
L2_3244_hg19_inputLibs_chr4_-_85791694	ESC-spec	.	15	0	.
L2_3244_hg19_inputLibs_chr4_+_104868089	ESC-spec	.	69	0	.
L2_3244_hg19_inputLibs_chr4_+_113573699	ESC-spec	.	44	0	.
L2_3244_hg19_inputLibs_chr4_+_12371205	ESC-spec	.	32	0	.
L2_3244_hg19_inputLibs_chr4_+_156942893	ESC-spec	.	39	0	.
L2_3244_hg19_inputLibs_chr4_+_187163423	ESC-spec	.	11	0	.
L2_3244_hg19_inputLibs_chr4_+_188916923	ESC-spec	ZFP42	22	0	mRNA
L2_3244_hg19_inputLibs_chr4_+_69313179	ESC-spec	TMPRSS11E	8	0	mRNA
L2_3244_hg19_inputLibs_chr4_+_79472922	ESC-spec	ANXA3	13	0	mRNA
L2_3244_hg19_inputLibs_chr4_+_84457638	ESC-spec	AGPAT9	9	0	mRNA
L2_3244_hg19_inputLibs_chr5_-_135072882	ESC-spec	.	19	0	.
L2_3244_hg19_inputLibs_chr5_-_36067006	ESC-spec	UGT3A2	15	0	mRNA
L2_3244_hg19_inputLibs_chr5_+_123833032	ESC-spec	.	29	0	.
L2_3244_hg19_inputLibs_chr5_+_128795376	ESC-spec	.	26	0	.
L2_3244_hg19_inputLibs_chr5_+_137419601	ESC-spec	WNT8A	89	0	mRNA
L2_3244_hg19_inputLibs_chr5_+_146939431	ESC-spec	JAKMIP2-AS1	21	0	RNA
L2_3244_hg19_inputLibs_chr5_+_146939557	ESC-spec	JAKMIP2-AS1	204	0	RNA
L2_3244_hg19_inputLibs_chr5_+_147251851	ESC-spec	.	22	0	.
L2_3244_hg19_inputLibs_chr5_+_149546342	ESC-spec	CDX1	19	0	mRNA
L2_3244_hg19_inputLibs_chr5_+_176558797	ESC-spec	.	124	0	.
L2_3244_hg19_inputLibs_chr5_+_176559861	ESC-spec	NSD1	21	0	mRNA
L2_3244_hg19_inputLibs_chr5_+_52777774	ESC-spec	.	12	0	.
L2_3244_hg19_inputLibs_chr5_+_52778873	ESC-spec	.	29	0	.
L2_3244_hg19_inputLibs_chr5_+_52779452	ESC-spec	.	29	0	.
L2_3244_hg19_inputLibs_chr5_+_52779945	ESC-spec	.	18	0	.
L2_3244_hg19_inputLibs_chr5_+_52780863	ESC-spec	.	22	0	.
L2_3244_hg19_inputLibs_chr5_+_52781858	ESC-spec	.	45	0	.
L2_3244_hg19_inputLibs_chr5_+_68710968	ESC-spec	MARVELD2	11	0	mRNA
L2_3244_hg19_inputLibs_chr6_-_137815531	ESC-spec	OLIG3	180	0	mRNA
L2_3244_hg19_inputLibs_chr6_-_138248623	ESC-spec	.	22	0	.
L2_3244_hg19_inputLibs_chr6_-_15022183	ESC-spec	.	16	0	.
L2_3244_hg19_inputLibs_chr6_-_166581343	ESC-spec	.	17	0	.
L2_3244_hg19_inputLibs_chr6_-_26018015	ESC-spec	HIST1H1A	18	0	mRNA
L2_3244_hg19_inputLibs_chr6_-_31138459	ESC-spec	POU5F1	83	0	mRNA
L2_3244_hg19_inputLibs_chr6_-_32634434	ESC-spec	HLA-DQB1	19	0	mRNA
L2_3244_hg19_inputLibs_chr6_-_42110348	ESC-spec	C6orf132	13	0	mRNA
L2_3244_hg19_inputLibs_chr6_-_74162010	ESC-spec	MB21D1	10	0	mRNA
L2_3244_hg19_inputLibs_chr6_+_10585993	ESC-spec	GCNT2	22	0	mRNA
L2_3244_hg19_inputLibs_chr6_+_131644779	ESC-spec	.	20	0	.
L2_3244_hg19_inputLibs_chr6_+_31430960	ESC-spec	HCP5	13	0	RNA
L2_3244_hg19_inputLibs_chr6_+_31545281	ESC-spec	.	18	0	.
L2_3244_hg19_inputLibs_chr6_+_4773262	ESC-spec	.	58	0	.
L2_3244_hg19_inputLibs_chr6_+_7541837	ESC-spec	DSP	12	0	mRNA
L2_3244_hg19_inputLibs_chr6_+_7569497	ESC-spec	.	7	0	.
L2_3244_hg19_inputLibs_chr7_-_102584358	ESC-spec	.	15	0	.
L2_3244_hg19_inputLibs_chr7_-_131196019	ESC-spec	.	11	0	.
L2_3244_hg19_inputLibs_chr7_-_143105957	ESC-spec	EPHA1	34	0	mRNA
L2_3244_hg19_inputLibs_chr7_-_20162717	ESC-spec	.	30	0	.

L2_3244_hg19_inputLibs_chr7_-_27135634	ESC-spec	HOXA1	40	0	mRNA
L2_3244_hg19_inputLibs_chr7_+_100770383	ESC-spec	SERPINE1	7	0	mRNA
L2_3244_hg19_inputLibs_chr7_+_100781800	ESC-spec	.	7	0	.
L2_3244_hg19_inputLibs_chr7_+_100951575	ESC-spec	.	15	0	.
L2_3244_hg19_inputLibs_chr7_+_102573985	ESC-spec	.	18	0	.
L2_3244_hg19_inputLibs_chr7_+_128349141	ESC-spec	FAM71F1	15	0	mRNA
L2_3244_hg19_inputLibs_chr8_-_110315725	ESC-spec	.	18	0	.
L2_3244_hg19_inputLibs_chr8_-_144815930	ESC-spec	FAM83H	8	0	mRNA
L2_3244_hg19_inputLibs_chr8_-_145013769	ESC-spec	PLEC	15	0	mRNA
L2_3244_hg19_inputLibs_chr8_-_70983449	ESC-spec	PRDM14	68	0	mRNA
L2_3244_hg19_inputLibs_chr8_-_81178412	ESC-spec	.	200	0	.
L2_3244_hg19_inputLibs_chr8_+_128428068	ESC-spec	POU5F1B	84	0	mRNA
L2_3244_hg19_inputLibs_chr8_+_132320860	ESC-spec	.	20	0	.
L2_3244_hg19_inputLibs_chr8_+_134420135	ESC-spec	.	15	0	.
L2_3244_hg19_inputLibs_chr8_+_143624336	ESC-spec	.	12	0	.
L2_3244_hg19_inputLibs_chr8_+_95653410	ESC-spec	ESRP1	33	0	mRNA
L2_3244_hg19_inputLibs_chr9_-_14722731	ESC-spec	CER1	108	0	mRNA
L2_3244_hg19_inputLibs_chr9_-_88499285	ESC-spec	.	20	0	.
L2_3244_hg19_inputLibs_chr9_+_118603166	ESC-spec	.	20	0	.
L2_3244_hg19_inputLibs_chr9_+_12948722	ESC-spec	.	11	0	.
L2_3244_hg19_inputLibs_chr9_+_131684572	ESC-spec	PHYHD1	31	0	mRNA
L2_3244_hg19_inputLibs_chr9_+_134020020	ESC-spec	.	24	0	.
L2_3244_hg19_inputLibs_chr9_+_27109271	ESC-spec	TEK	16	0	mRNA
L2_3244_hg19_inputLibs_chr9_+_34991345	ESC-spec	.	16	0	.
L2_3244_hg19_inputLibs_chr9_+_72435731	ESC-spec	C9orf135	20	0	mRNA
L2_3244_hg19_inputLibs_chrX_-_116059025	ESC-spec	.	22	0	.
L2_3244_hg19_inputLibs_chrX_-_131842664	ESC-spec	.	55	0	.
L2_3244_hg19_inputLibs_chrX_-_16202185	ESC-spec	.	13	0	.
L2_3244_hg19_inputLibs_chrX_-_16235031	ESC-spec	.	11	0	.
L2_3244_hg19_inputLibs_chrX_-_48326723	ESC-spec	.	8	0	.
L2_3244_hg19_inputLibs_chrX_-_48327777	ESC-spec	.	18	0	.
L2_3244_hg19_inputLibs_chrX_-_78622828	ESC-spec	ITM2A	9	0	mRNA
L2_3244_hg19_inputLibs_chrX_-_9733887	ESC-spec	GPR143	67	0	mRNA
L2_3244_hg19_inputLibs_chrX_+_72749800	ESC-spec	.	17	0	.
L2_3244_hg19_inputLibs_chrX_+_86937790	ESC-spec	.	15	0	.
L2_3244_hg19_inputLibs_chr1_-_103548477	NESC-spec	.	0	13	.
L2_3244_hg19_inputLibs_chr1_-_198906543	NESC-spec	MIR181A1HG	0	9	RNA
L2_3244_hg19_inputLibs_chr1_-_204320090	NESC-spec	.	0	13	.
L2_3244_hg19_inputLibs_chr1_-_206288236	NESC-spec	C1orf186	0	13	mRNA
L2_3244_hg19_inputLibs_chr1_-_98386585	NESC-spec	DPYD	0	25	mRNA
L2_3244_hg19_inputLibs_chr1_+_2005138	NESC-spec	PRKCZ	0	11	mRNA
L2_3244_hg19_inputLibs_chr1_+_25943311	NESC-spec	.	0	23	.
L2_3244_hg19_inputLibs_chr1_+_51434477	NESC-spec	CDKN2C	0	11	mRNA
L2_3244_hg19_inputLibs_chr1_+_59858839	NESC-spec	.	0	45	.
L2_3244_hg19_inputLibs_chr1_+_60560218	NESC-spec	.	0	80	.
L2_3244_hg19_inputLibs_chr10_-_17492284	NESC-spec	.	0	9	.
L2_3244_hg19_inputLibs_chr10_-_22218027	NESC-spec	.	0	53	.
L2_3244_hg19_inputLibs_chr10_-_48439106	NESC-spec	GDF10	0	15	mRNA
L2_3244_hg19_inputLibs_chr10_-_62332397	NESC-spec	ANK3	0	12	mRNA

L2_3244_hg19_inputLibs_chr10_+_11047370	NESC-spec	CELF2	0	26	mRNA
L2_3244_hg19_inputLibs_chr10_+_120967253	NESC-spec	GRK5	0	11	mRNA
L2_3244_hg19_inputLibs_chr10_+_63809020	NESC-spec	ARID5B	0	12	mRNA
L2_3244_hg19_inputLibs_chr10_+_92922613	NESC-spec	PCGF5	0	10	mRNA
L2_3244_hg19_inputLibs_chr10_+_92922748	NESC-spec	PCGF5	0	12	mRNA
L2_3244_hg19_inputLibs_chr11_-_121971156	NESC-spec	.	0	23	.
L2_3244_hg19_inputLibs_chr11_-_125929331	NESC-spec	.	0	25	.
L2_3244_hg19_inputLibs_chr11_-_31832870	NESC-spec	PAX6	0	35	mRNA
L2_3244_hg19_inputLibs_chr11_-_72433325	NESC-spec	ARAP1	0	9	mRNA
L2_3244_hg19_inputLibs_chr11_+_113930464	NESC-spec	ZBTB16	0	11	mRNA
L2_3244_hg19_inputLibs_chr11_+_118478318	NESC-spec	PHLDB1	0	11	mRNA
L2_3244_hg19_inputLibs_chr11_+_60680203	NESC-spec	.	0	23	.
L2_3244_hg19_inputLibs_chr11_+_7506758	NESC-spec	OLFML1	0	23	mRNA
L2_3244_hg19_inputLibs_chr11_+_7506926	NESC-spec	OLFML1	0	14	mRNA
L2_3244_hg19_inputLibs_chr11_+_7507073	NESC-spec	OLFML1	0	12	mRNA
L2_3244_hg19_inputLibs_chr12_-_122907850	NESC-spec	.	0	28	.
L2_3244_hg19_inputLibs_chr12_-_22063813	NESC-spec	.	0	13	.
L2_3244_hg19_inputLibs_chr12_-_22065857	NESC-spec	.	0	38	.
L2_3244_hg19_inputLibs_chr12_-_48398111	NESC-spec	COL2A1	0	21	mRNA
L2_3244_hg19_inputLibs_chr12_-_48398435	NESC-spec	COL2A1	0	37	mRNA
L2_3244_hg19_inputLibs_chr12_-_49579486	NESC-spec	.	0	10	.
L2_3244_hg19_inputLibs_chr12_-_58026982	NESC-spec	B4GALNT1	0	12	mRNA
L2_3244_hg19_inputLibs_chr12_-_9268507	NESC-spec	A2M	0	169	mRNA
L2_3244_hg19_inputLibs_chr12_-_9268773	NESC-spec	A2M	0	14	mRNA
L2_3244_hg19_inputLibs_chr12_-_99548542	NESC-spec	ANKS1B	0	10	mRNA
L2_3244_hg19_inputLibs_chr12_+_103351450	NESC-spec	ASCL1	0	29	mRNA
L2_3244_hg19_inputLibs_chr12_+_20521480	NESC-spec	.	0	19	.
L2_3244_hg19_inputLibs_chr12_+_41831487	NESC-spec	PDZRN4	0	12	mRNA
L2_3244_hg19_inputLibs_chr13_-_25746426	NESC-spec	.	0	10	.
L2_3244_hg19_inputLibs_chr13_-_33924817	NESC-spec	.	0	12	.
L2_3244_hg19_inputLibs_chr13_-_38172905	NESC-spec	POSTN	0	9	mRNA
L2_3244_hg19_inputLibs_chr13_-_88323423	NESC-spec	MIR4500HG	0	26	RNA
L2_3244_hg19_inputLibs_chr13_+_112721219	NESC-spec	.	0	39	.
L2_3244_hg19_inputLibs_chr13_+_63967431	NESC-spec	.	0	11	.
L2_3244_hg19_inputLibs_chr14_-_26514905	NESC-spec	.	0	17	.
L2_3244_hg19_inputLibs_chr14_-_26514997	NESC-spec	.	0	12	.
L2_3244_hg19_inputLibs_chr14_-_51027845	NESC-spec	.	0	11	.
L2_3244_hg19_inputLibs_chr14_+_22978172	NESC-spec	.	0	11	.
L2_3244_hg19_inputLibs_chr14_+_63671631	NESC-spec	.	0	64	.
L2_3244_hg19_inputLibs_chr14_+_70346131	NESC-spec	SMOC1	0	11	mRNA
L2_3244_hg19_inputLibs_chr15_-_52944165	NESC-spec	FAM214A	0	12	mRNA
L2_3244_hg19_inputLibs_chr15_-_53082533	NESC-spec	ONECUT1	0	22	mRNA
L2_3244_hg19_inputLibs_chr15_+_48009662	NESC-spec	.	0	12	.
L2_3244_hg19_inputLibs_chr15_+_93856433	NESC-spec	.	0	21	.
L2_3244_hg19_inputLibs_chr15_+_96876992	NESC-spec	NR2F2	0	13	mRNA
L2_3244_hg19_inputLibs_chr16_+_47999843	NESC-spec	.	0	16	.
L2_3244_hg19_inputLibs_chr17_-_15168643	NESC-spec	PMP22	0	63	mRNA
L2_3244_hg19_inputLibs_chr17_-_17726918	NESC-spec	.	0	9	.
L2_3244_hg19_inputLibs_chr17_-_33700640	NESC-spec	SLFN11	0	12	mRNA

L2_3244_hg19_inputLibs_chr17_-_46622471	NESC-spec	HOXB2	0	12	mRNA
L2_3244_hg19_inputLibs_chr17_-_48207536	NESC-spec	SAMD14	0	12	mRNA
L2_3244_hg19_inputLibs_chr17_-_50647562	NESC-spec	.	0	18	.
L2_3244_hg19_inputLibs_chr17_-_66951519	NESC-spec	ABCA8	0	32	mRNA
L2_3244_hg19_inputLibs_chr17_+_14204403	NESC-spec	HS3ST3B1	0	13	mRNA
L2_3244_hg19_inputLibs_chr17_+_59477246	NESC-spec	TBX2	0	28	mRNA
L2_3244_hg19_inputLibs_chr18_-_7117993	NESC-spec	LAMA1	0	11	mRNA
L2_3244_hg19_inputLibs_chr18_+_23113560	NESC-spec	.	0	11	.
L2_3244_hg19_inputLibs_chr18_+_49866565	NESC-spec	DCC	0	65	mRNA
L2_3244_hg19_inputLibs_chr18_+_49866638	NESC-spec	DCC	0	18	mRNA
L2_3244_hg19_inputLibs_chr18_+_49867049	NESC-spec	.	0	22	.
L2_3244_hg19_inputLibs_chr18_+_50985729	NESC-spec	.	0	12	.
L2_3244_hg19_inputLibs_chr18_+_6774010	NESC-spec	.	0	16	.
L2_3244_hg19_inputLibs_chr19_-_46477253	NESC-spec	.	0	16	.
L2_3244_hg19_inputLibs_chr19_-_54984412	NESC-spec	CDC42EP5	0	30	mRNA
L2_3244_hg19_inputLibs_chr19_+_54926636	NESC-spec	TTYH1	0	18	mRNA
L2_3244_hg19_inputLibs_chr2_-_105467911	NESC-spec	LOC100506421	0	149	RNA
L2_3244_hg19_inputLibs_chr2_-_105488816	NESC-spec	.	0	31	.
L2_3244_hg19_inputLibs_chr2_-_174830592	NESC-spec	SP3	0	10	mRNA
L2_3244_hg19_inputLibs_chr2_-_180427271	NESC-spec	ZNF385B	0	30	mRNA
L2_3244_hg19_inputLibs_chr2_-_183387388	NESC-spec	PDE1A	0	43	mRNA
L2_3244_hg19_inputLibs_chr2_-_218867718	NESC-spec	.	0	16	.
L2_3244_hg19_inputLibs_chr2_-_225847144	NESC-spec	.	0	15	.
L2_3244_hg19_inputLibs_chr2_-_288712	NESC-spec	FAM150B	0	16	mRNA
L2_3244_hg19_inputLibs_chr2_-_288836	NESC-spec	.	0	17	.
L2_3244_hg19_inputLibs_chr2_-_56241203	NESC-spec	.	0	16	.
L2_3244_hg19_inputLibs_chr2_+_105469494	NESC-spec	.	0	12	.
L2_3244_hg19_inputLibs_chr2_+_105469743	NESC-spec	.	0	22	.
L2_3244_hg19_inputLibs_chr2_+_105470541	NESC-spec	.	0	259	.
L2_3244_hg19_inputLibs_chr2_+_112895991	NESC-spec	FBLN7	0	11	mRNA
L2_3244_hg19_inputLibs_chr2_+_14772839	NESC-spec	FAM84A	0	14	mRNA
L2_3244_hg19_inputLibs_chr2_+_189838913	NESC-spec	COL3A1	0	13	mRNA
L2_3244_hg19_inputLibs_chr2_+_71127737	NESC-spec	VAX2	0	11	mRNA
L2_3244_hg19_inputLibs_chr20_-_23066961	NESC-spec	CD93	0	7	mRNA
L2_3244_hg19_inputLibs_chr20_-_36889113	NESC-spec	KIAA1755	0	15	mRNA
L2_3244_hg19_inputLibs_chr20_+_20348748	NESC-spec	INSM1	0	13	mRNA
L2_3244_hg19_inputLibs_chr21_-_15295639	NESC-spec	.	0	11	.
L2_3244_hg19_inputLibs_chr21_-_48024995	NESC-spec	S100B	0	12	mRNA
L2_3244_hg19_inputLibs_chr21_+_17652060	NESC-spec	.	0	13	.
L2_3244_hg19_inputLibs_chr22_+_32601145	NESC-spec	.	0	7	.
L2_3244_hg19_inputLibs_chr3_-_123518755	NESC-spec	.	0	17	.
L2_3244_hg19_inputLibs_chr3_-_148939844	NESC-spec	CP	0	43	mRNA
L2_3244_hg19_inputLibs_chr3_-_155364896	NESC-spec	.	0	43	.
L2_3244_hg19_inputLibs_chr3_-_155394124	NESC-spec	PLCH1	0	14	mRNA
L2_3244_hg19_inputLibs_chr3_-_157221175	NESC-spec	VEPH1	0	25	mRNA
L2_3244_hg19_inputLibs_chr3_-_171489108	NESC-spec	.	0	9	.
L2_3244_hg19_inputLibs_chr3_-_47620355	NESC-spec	CSPG5	0	48	mRNA
L2_3244_hg19_inputLibs_chr3_-_79817171	NESC-spec	ROBO1	0	15	mRNA
L2_3244_hg19_inputLibs_chr3_-_8686484	NESC-spec	SSUH2	0	52	mRNA

L2_3244_hg19_inputLibs_chr3_+_128720165	NESC-spec	EFCC1	0	36	mRNA
L2_3244_hg19_inputLibs_chr3_+_128720446	NESC-spec	EFCC1	0	11	mRNA
L2_3244_hg19_inputLibs_chr3_+_147127920	NESC-spec	.	0	24	.
L2_3244_hg19_inputLibs_chr3_+_157095458	NESC-spec	.	0	13	.
L2_3244_hg19_inputLibs_chr3_+_157154397	NESC-spec	PTX3	0	14	mRNA
L2_3244_hg19_inputLibs_chr3_+_157160369	NESC-spec	.	0	49	.
L2_3244_hg19_inputLibs_chr3_+_48507652	NESC-spec	.	0	8	.
L2_3244_hg19_inputLibs_chr3_+_89156839	NESC-spec	EPHA3	0	20	mRNA
L2_3244_hg19_inputLibs_chr4_-_116034776	NESC-spec	NDST4	0	12	mRNA
L2_3244_hg19_inputLibs_chr4_-_116034996	NESC-spec	NDST4	0	11	mRNA
L2_3244_hg19_inputLibs_chr4_-_176923564	NESC-spec	GPM6A	0	49	mRNA
L2_3244_hg19_inputLibs_chr4_-_180386695	NESC-spec	.	0	40	.
L2_3244_hg19_inputLibs_chr4_-_186733283	NESC-spec	SORBS2	0	32	mRNA
L2_3244_hg19_inputLibs_chr4_-_88415687	NESC-spec	.	0	16	.
L2_3244_hg19_inputLibs_chr4_-_96470124	NESC-spec	UNC5C	0	11	mRNA
L2_3244_hg19_inputLibs_chr4_+_52917583	NESC-spec	SPATA18	0	12	mRNA
L2_3244_hg19_inputLibs_chr4_+_55604692	NESC-spec	.	0	15	.
L2_3244_hg19_inputLibs_chr4_+_71587639	NESC-spec	RUFY3	0	36	mRNA
L2_3244_hg19_inputLibs_chr4_+_81951811	NESC-spec	BMP3	0	14	mRNA
L2_3244_hg19_inputLibs_chr4_+_90816003	NESC-spec	MMRN1	0	59	mRNA
L2_3244_hg19_inputLibs_chr4_+_91740311	NESC-spec	.	0	33	.
L2_3244_hg19_inputLibs_chr5_-_111755831	NESC-spec	.	0	20	.
L2_3244_hg19_inputLibs_chr5_-_11589049	NESC-spec	.	0	31	.
L2_3244_hg19_inputLibs_chr5_-_136834999	NESC-spec	SPOCK1	0	90	mRNA
L2_3244_hg19_inputLibs_chr5_-_87969136	NESC-spec	LINC00461	0	25	RNA
L2_3244_hg19_inputLibs_chr5_-_92957174	NESC-spec	.	0	12	.
L2_3244_hg19_inputLibs_chr5_+_14441200	NESC-spec	.	0	16	.
L2_3244_hg19_inputLibs_chr5_+_163723973	NESC-spec	.	0	69	.
L2_3244_hg19_inputLibs_chr5_+_38556988	NESC-spec	LIFR-AS1	0	11	RNA
L2_3244_hg19_inputLibs_chr5_+_8461925	NESC-spec	.	0	16	.
L2_3244_hg19_inputLibs_chr5_+_92918793	NESC-spec	NR2F1	0	13	mRNA
L2_3244_hg19_inputLibs_chr5_+_92919129	NESC-spec	NR2F1	0	23	mRNA
L2_3244_hg19_inputLibs_chr5_+_92922011	NESC-spec	.	0	11	.
L2_3244_hg19_inputLibs_chr5_+_92922809	NESC-spec	MIR548AO	0	16	RNA
L2_3244_hg19_inputLibs_chr5_+_92923087	NESC-spec	MIR548AO	0	12	RNA
L2_3244_hg19_inputLibs_chr5_+_92923424	NESC-spec	.	0	15	.
L2_3244_hg19_inputLibs_chr6_-_112575924	NESC-spec	LAMA4	0	11	mRNA
L2_3244_hg19_inputLibs_chr6_-_114194512	NESC-spec	LOC285758	0	42	RNA
L2_3244_hg19_inputLibs_chr6_-_25042396	NESC-spec	FAM65B	0	18	mRNA
L2_3244_hg19_inputLibs_chr6_+_50786064	NESC-spec	TFAP2B	0	27	mRNA
L2_3244_hg19_inputLibs_chr6_+_50789736	NESC-spec	.	0	12	.
L2_3244_hg19_inputLibs_chr6_+_99282601	NESC-spec	POU3F2	0	18	mRNA
L2_3244_hg19_inputLibs_chr7_-_150945743	NESC-spec	SMARCD3	0	12	mRNA
L2_3244_hg19_inputLibs_chr7_-_157483658	NESC-spec	.	0	106	.
L2_3244_hg19_inputLibs_chr7_-_19157260	NESC-spec	TWIST1	0	30	mRNA
L2_3244_hg19_inputLibs_chr7_-_92747293	NESC-spec	SAMD9	0	7	mRNA
L2_3244_hg19_inputLibs_chr7_+_102553448	NESC-spec	LRRC17	0	25	mRNA
L2_3244_hg19_inputLibs_chr7_+_114055089	NESC-spec	FOXP2	0	82	mRNA
L2_3244_hg19_inputLibs_chr7_+_155250259	NESC-spec	.	0	123	.

L2_3244_hg19_inputLibs_chr7_+_157257267	NESC-spec	.	0	41	.
L2_3244_hg19_inputLibs_chr7_+_16955813	NESC-spec	.	0	25	.
L2_3244_hg19_inputLibs_chr7_+_16955981	NESC-spec	.	0	27	.
L2_3244_hg19_inputLibs_chr7_+_85961153	NESC-spec	.	0	30	.
L2_3244_hg19_inputLibs_chr7_+_86273232	NESC-spec	GRM3	0	13	mRNA
L2_3244_hg19_inputLibs_chr7_+_86273904	NESC-spec	.	0	14	.
L2_3244_hg19_inputLibs_chr7_+_93550675	NESC-spec	GNG11	0	20	mRNA
L2_3244_hg19_inputLibs_chr8_-_124428598	NESC-spec	.	0	16	.
L2_3244_hg19_inputLibs_chr8_-_13372418	NESC-spec	DLC1	0	12	mRNA
L2_3244_hg19_inputLibs_chr8_-_17579485	NESC-spec	MTUS1	0	27	mRNA
L2_3244_hg19_inputLibs_chr8_-_28352916	NESC-spec	.	0	53	.
L2_3244_hg19_inputLibs_chr8_-_65281146	NESC-spec	.	0	11	.
L2_3244_hg19_inputLibs_chr8_-_72274480	NESC-spec	EYA1	0	11	mRNA
L2_3244_hg19_inputLibs_chr8_-_95961587	NESC-spec	TP53INP1	0	12	mRNA
L2_3244_hg19_inputLibs_chr8_+_104289639	NESC-spec	.	0	23	.
L2_3244_hg19_inputLibs_chr8_+_104290202	NESC-spec	.	0	28	.
L2_3244_hg19_inputLibs_chr8_+_19796765	NESC-spec	LPL	0	171	mRNA
L2_3244_hg19_inputLibs_chr8_+_40010999	NESC-spec	C8orf4	0	131	mRNA
L2_3244_hg19_inputLibs_chr8_+_54792977	NESC-spec	.	0	11	.
L2_3244_hg19_inputLibs_chr8_+_93029533	NESC-spec	.	0	53	.
L2_3244_hg19_inputLibs_chr9_-_113800750	NESC-spec	LPAR1	0	32	mRNA
L2_3244_hg19_inputLibs_chr9_-_14869015	NESC-spec	.	0	13	.
L2_3244_hg19_inputLibs_chr9_-_27497980	NESC-spec	.	0	11	.
L2_3244_hg19_inputLibs_chr9_-_38423997	NESC-spec	.	0	10	.
L2_3244_hg19_inputLibs_chrX_-_13835425	NESC-spec	GPM6B	0	25	mRNA
L2_3244_hg19_inputLibs_chrX_-_32173588	NESC-spec	DMD	0	25	mRNA
L2_3244_hg19_inputLibs_chrX_-_33229666	NESC-spec	DMD	0	20	mRNA
L2_3244_hg19_inputLibs_chrX_+_100265415	NESC-spec	.	0	63	.
L2_3244_hg19_inputLibs_chr1_-_103574039	upregulated	COL11A1	32	436	mRNA
L2_3244_hg19_inputLibs_chr1_-_151119143	upregulated	SEMA6C	1	11	mRNA
L2_3244_hg19_inputLibs_chr1_-_154164576	upregulated	TPM3	2	23	mRNA
L2_3244_hg19_inputLibs_chr1_-_1551177	upregulated	.	1	13	.
L2_3244_hg19_inputLibs_chr1_-_178840203	upregulated	ANGPTL1	1	52	mRNA
L2_3244_hg19_inputLibs_chr1_-_21113798	upregulated	.	15	124	.
L2_3244_hg19_inputLibs_chr1_-_236228390	upregulated	NID1	42	460	mRNA
L2_3244_hg19_inputLibs_chr1_-_31230621	upregulated	LAPTM5	1	15	mRNA
L2_3244_hg19_inputLibs_chr1_-_31381605	upregulated	SDC3	24	198	mRNA
L2_3244_hg19_inputLibs_chr1_-_79472384	upregulated	ELTD1	3	61	mRNA
L2_3244_hg19_inputLibs_chr1_-_94703140	upregulated	ARHGAP29	3	27	mRNA
L2_3244_hg19_inputLibs_chr1_+_101702573	upregulated	S1PR1	1	11	mRNA
L2_3244_hg19_inputLibs_chr1_+_145438488	upregulated	TXNIP	2	17	mRNA
L2_3244_hg19_inputLibs_chr1_+_155829291	upregulated	SYT11	2	20	mRNA
L2_3244_hg19_inputLibs_chr1_+_170632728	upregulated	.	1	15	.
L2_3244_hg19_inputLibs_chr1_+_203444946	upregulated	PRELP	1	12	mRNA
L2_3244_hg19_inputLibs_chr1_+_79086153	upregulated	IFI44L	3	66	mRNA
L2_3244_hg19_inputLibs_chr1_+_81771793	upregulated	.	1	14	.
L2_3244_hg19_inputLibs_chr1_+_81772004	upregulated	.	1	140	.
L2_3244_hg19_inputLibs_chr10_-_118031849	upregulated	GFRA1	2	20	mRNA
L2_3244_hg19_inputLibs_chr10_-_118032176	upregulated	GFRA1	2	17	mRNA

L2_3244_hg19_inputLibs_chr10_-_13043756	upregulated	CCDC3	1	11	mRNA
L2_3244_hg19_inputLibs_chr10_-_13389670	upregulated	.	2	26	.
L2_3244_hg19_inputLibs_chr10_-_21806761	upregulated	.	2	24	.
L2_3244_hg19_inputLibs_chr10_-_50323554	upregulated	VSTM4	2	41	mRNA
L2_3244_hg19_inputLibs_chr10_+_102107109	upregulated	SCD	6	109	mRNA
L2_3244_hg19_inputLibs_chr10_+_102133385	upregulated	LINC00263	12	162	RNA
L2_3244_hg19_inputLibs_chr10_+_111967361	upregulated	MXI1	2	22	mRNA
L2_3244_hg19_inputLibs_chr10_+_134000452	upregulated	DPYSL4	2	16	mRNA
L2_3244_hg19_inputLibs_chr10_+_63661456	upregulated	ARID5B	1	19	mRNA
L2_3244_hg19_inputLibs_chr10_+_82224710	upregulated	.	2	20	.
L2_3244_hg19_inputLibs_chr11_-_125932748	upregulated	CDON	7	68	mRNA
L2_3244_hg19_inputLibs_chr11_-_128392205	upregulated	ETS1	4	74	mRNA
L2_3244_hg19_inputLibs_chr11_-_47615935	upregulated	C1QTNF4	2	29	mRNA
L2_3244_hg19_inputLibs_chr11_-_65325712	upregulated	LTBP3	1	11	mRNA
L2_3244_hg19_inputLibs_chr11_-_85430016	upregulated	SYTL2	1	12	mRNA
L2_3244_hg19_inputLibs_chr11_-_94964364	upregulated	SESN3	4	38	mRNA
L2_3244_hg19_inputLibs_chr11_+_110001727	upregulated	.	1	45	.
L2_3244_hg19_inputLibs_chr11_+_61596027	upregulated	.	1	11	.
L2_3244_hg19_inputLibs_chr11_+_65340007	upregulated	FAM89B	1	10	mRNA
L2_3244_hg19_inputLibs_chr11_+_86511584	upregulated	PRSS23	5	66	mRNA
L2_3244_hg19_inputLibs_chr12_-_106532732	upregulated	.	7	67	.
L2_3244_hg19_inputLibs_chr12_-_15114525	upregulated	ARHGDIB	2	21	mRNA
L2_3244_hg19_inputLibs_chr12_-_24102591	upregulated	SOX5	2	31	mRNA
L2_3244_hg19_inputLibs_chr12_-_45269373	upregulated	NELL2	1	20	mRNA
L2_3244_hg19_inputLibs_chr12_-_48398259	upregulated	COL2A1	3	345	mRNA
L2_3244_hg19_inputLibs_chr12_-_91505257	upregulated	LUM	3	133	mRNA
L2_3244_hg19_inputLibs_chr12_-_91505362	upregulated	LUM	1	21	mRNA
L2_3244_hg19_inputLibs_chr12_+_106696593	upregulated	TCP11L2	2	20	mRNA
L2_3244_hg19_inputLibs_chr12_+_106976683	upregulated	RFX4	1	20	mRNA
L2_3244_hg19_inputLibs_chr12_+_106976779	upregulated	RFX4	23	266	mRNA
L2_3244_hg19_inputLibs_chr12_+_12938591	upregulated	APOLD1	2	16	mRNA
L2_3244_hg19_inputLibs_chr12_+_14538187	upregulated	ATF7IP	4	85	mRNA
L2_3244_hg19_inputLibs_chr12_+_14569832	upregulated	.	1	34	.
L2_3244_hg19_inputLibs_chr12_+_14570900	upregulated	.	2	32	.
L2_3244_hg19_inputLibs_chr12_+_20522212	upregulated	PDE3A	1	12	mRNA
L2_3244_hg19_inputLibs_chr12_+_20522628	upregulated	PDE3A	2	91	mRNA
L2_3244_hg19_inputLibs_chr12_+_48357388	upregulated	TMEM106C	6	59	mRNA
L2_3244_hg19_inputLibs_chr12_+_48577410	upregulated	C12orf68	1	17	mRNA
L2_3244_hg19_inputLibs_chr12_+_56136904	upregulated	GDF11	2	26	mRNA
L2_3244_hg19_inputLibs_chr12_+_64238479	upregulated	SRGAP1	1	12	mRNA
L2_3244_hg19_inputLibs_chr13_-_25745708	upregulated	AMER2	1	18	mRNA
L2_3244_hg19_inputLibs_chr13_-_36705467	upregulated	DCLK1	1	29	mRNA
L2_3244_hg19_inputLibs_chr13_-_40177473	upregulated	LHFP	1	10	mRNA
L2_3244_hg19_inputLibs_chr13_-_67804468	upregulated	PCDH9	4	79	mRNA
L2_3244_hg19_inputLibs_chr13_-_95364790	upregulated	SOX21	5	44	mRNA
L2_3244_hg19_inputLibs_chr13_+_111767677	upregulated	ARHGEF7	3	71	mRNA
L2_3244_hg19_inputLibs_chr13_+_113656096	upregulated	MCF2L	1	17	mRNA
L2_3244_hg19_inputLibs_chr13_+_24144579	upregulated	TNFRSF19	9	83	mRNA
L2_3244_hg19_inputLibs_chr13_+_38489359	upregulated	.	1	21	.

L2_3244_hg19_inputLibs_chr13_+_58204240	upregulated	.	3	59	.
L2_3244_hg19_inputLibs_chr13_+_95364967	upregulated	SOX21-AS1	2	37	RNA
L2_3244_hg19_inputLibs_chr14_-_52535776	upregulated	NID2	17	162	mRNA
L2_3244_hg19_inputLibs_chr14_+_23352429	upregulated	REM2	2	18	mRNA
L2_3244_hg19_inputLibs_chr14_+_58797995	upregulated	.	2	32	.
L2_3244_hg19_inputLibs_chr14_+_63671141	upregulated	RHOJ	1	121	mRNA
L2_3244_hg19_inputLibs_chr14_+_85996486	upregulated	FLRT2	2	21	mRNA
L2_3244_hg19_inputLibs_chr14_+_94577080	upregulated	IFI27	1	13	mRNA
L2_3244_hg19_inputLibs_chr14_+_94577080	upregulated	IFI27	1	13	mRNA
L2_3244_hg19_inputLibs_chr15_-_40401075	upregulated	BMF	3	31	mRNA
L2_3244_hg19_inputLibs_chr15_-_40401075	upregulated	BMF	3	31	mRNA
L2_3244_hg19_inputLibs_chr15_-_79103697	upregulated	ADAMTS7	1	12	mRNA
L2_3244_hg19_inputLibs_chr15_+_51973686	upregulated	SCG3	2	21	mRNA
L2_3244_hg19_inputLibs_chr15_+_57511623	upregulated	TCF12	21	175	mRNA
L2_3244_hg19_inputLibs_chr15_+_57543574	upregulated	.	2	18	.
L2_3244_hg19_inputLibs_chr15_+_80696738	upregulated	ARNT2	3	27	mRNA
L2_3244_hg19_inputLibs_chr15_+_96869274	upregulated	NR2F2	2	16	mRNA
L2_3244_hg19_inputLibs_chr16_-_52581034	upregulated	TOX3	5	42	mRNA
L2_3244_hg19_inputLibs_chr16_-_54320117	upregulated	IRX3	1	10	mRNA
L2_3244_hg19_inputLibs_chr16_-_54320667	upregulated	IRX3	4	38	mRNA
L2_3244_hg19_inputLibs_chr16_-_79633102	upregulated	.	1	21	.
L2_3244_hg19_inputLibs_chr16_-_79634631	upregulated	MAF	2	19	mRNA
L2_3244_hg19_inputLibs_chr16_+_30907934	upregulated	CTF1	2	16	mRNA
L2_3244_hg19_inputLibs_chr16_+_8768473	upregulated	ABAT	5	42	mRNA
L2_3244_hg19_inputLibs_chr17_-_15142827	upregulated	.	2	22	.
L2_3244_hg19_inputLibs_chr17_-_15165833	upregulated	PMP22	3	43	mRNA
L2_3244_hg19_inputLibs_chr17_-_48207171	upregulated	SAMD14	2	23	mRNA
L2_3244_hg19_inputLibs_chr17_-_48207435	upregulated	SAMD14	1	17	mRNA
L2_3244_hg19_inputLibs_chr17_+_32582302	upregulated	CCL2	34	309	mRNA
L2_3244_hg19_inputLibs_chr17_+_46088802	upregulated	.	4	54	.
L2_3244_hg19_inputLibs_chr17_+_57409014	upregulated	YPEL2	2	19	mRNA
L2_3244_hg19_inputLibs_chr17_+_61228447	upregulated	.	1	43	.
L2_3244_hg19_inputLibs_chr17_+_70117159	upregulated	SOX9	4	70	mRNA
L2_3244_hg19_inputLibs_chr17_+_7608545	upregulated	EFNB3	8	80	mRNA
L2_3244_hg19_inputLibs_chr18_-_500698	upregulated	COLEC12	1	28	mRNA
L2_3244_hg19_inputLibs_chr18_+_32290260	upregulated	DTNA	4	32	mRNA
L2_3244_hg19_inputLibs_chr18_+_67068101	upregulated	DOK6	1	12	mRNA
L2_3244_hg19_inputLibs_chr19_-_10679509	upregulated	CDKN2D	3	28	mRNA
L2_3244_hg19_inputLibs_chr19_-_17488154	upregulated	PLVAP	1	11	mRNA
L2_3244_hg19_inputLibs_chr19_-_18548941	upregulated	ISYNA1	14	125	mRNA
L2_3244_hg19_inputLibs_chr19_-_3062504	upregulated	AES	2	19	mRNA
L2_3244_hg19_inputLibs_chr19_-_571749	upregulated	.	3	24	.
L2_3244_hg19_inputLibs_chr19_-_822059	upregulated	LPPR3	1	11	mRNA
L2_3244_hg19_inputLibs_chr19_+_36359440	upregulated	APLP1	4	46	mRNA
L2_3244_hg19_inputLibs_chr19_+_41103116	upregulated	LTBP4	3	34	mRNA
L2_3244_hg19_inputLibs_chr19_+_7895105	upregulated	EVI5L	2	17	mRNA
L2_3244_hg19_inputLibs_chr2_-_183731317	upregulated	FRZB	8	105	mRNA
L2_3244_hg19_inputLibs_chr2_-_183731890	upregulated	FRZB	2	27	mRNA
L2_3244_hg19_inputLibs_chr2_-_188419162	upregulated	TFPI	13	241	mRNA

L2_3244_hg19_inputLibs_chr2_-_192711933	upregulated	SDPR	1	11	mRNA
L2_3244_hg19_inputLibs_chr2_-_197791431	upregulated	PGAP1	2	25	mRNA
L2_3244_hg19_inputLibs_chr2_-_198540194	upregulated	RFTN2	1	14	mRNA
L2_3244_hg19_inputLibs_chr2_-_198540497	upregulated	RFTN2	2	74	mRNA
L2_3244_hg19_inputLibs_chr2_-_214015171	upregulated	IKZF2	1	12	mRNA
L2_3244_hg19_inputLibs_chr2_-_217543631	upregulated	.	2	23	.
L2_3244_hg19_inputLibs_chr2_-_217559327	upregulated	.	2	19	.
L2_3244_hg19_inputLibs_chr2_-_217559838	upregulated	.	1	13	.
L2_3244_hg19_inputLibs_chr2_-_217560270	upregulated	IGFBP5	25	285	mRNA
L2_3244_hg19_inputLibs_chr2_-_230579245	upregulated	DNER	1	19	mRNA
L2_3244_hg19_inputLibs_chr2_-_238322807	upregulated	COL6A3	1	11	mRNA
L2_3244_hg19_inputLibs_chr2_-_287896	upregulated	FAM150B	2	17	mRNA
L2_3244_hg19_inputLibs_chr2_-_68546548	upregulated	CNRIP1	1	49	mRNA
L2_3244_hg19_inputLibs_chr2_-_68546548	upregulated	CNRIP1	1	49	mRNA
L2_3244_hg19_inputLibs_chr2_+_11886756	upregulated	LPIN1	1	13	mRNA
L2_3244_hg19_inputLibs_chr2_+_144361209	upregulated	.	1	15	.
L2_3244_hg19_inputLibs_chr2_+_181845851	upregulated	UBE2E3	6	201	mRNA
L2_3244_hg19_inputLibs_chr2_+_181848760	upregulated	.	2	16	.
L2_3244_hg19_inputLibs_chr2_+_182322112	upregulated	.	1	10	.
L2_3244_hg19_inputLibs_chr2_+_182756923	upregulated	SSFA2	4	73	mRNA
L2_3244_hg19_inputLibs_chr2_+_189839098	upregulated	COL3A1	55	766	mRNA
L2_3244_hg19_inputLibs_chr2_+_210444085	upregulated	MAP2	9	82	mRNA
L2_3244_hg19_inputLibs_chr2_+_210526221	upregulated	.	1	36	.
L2_3244_hg19_inputLibs_chr2_+_39664581	upregulated	LOC728730	2	25	RNA
L2_3244_hg19_inputLibs_chr2_+_39893095	upregulated	TMEM178A	4	66	mRNA
L2_3244_hg19_inputLibs_chr2_+_48757324	upregulated	STON1	2	16	mRNA
L2_3244_hg19_inputLibs_chr2_+_48757324	upregulated	STON1-GTF2A1L	2	16	mRNA
L2_3244_hg19_inputLibs_chr2_+_66662514	upregulated	MEIS1	2	23	mRNA
L2_3244_hg19_inputLibs_chr2_+_79740146	upregulated	CTNNA2	1	12	mRNA
L2_3244_hg19_inputLibs_chr20_-_14318220	upregulated	FLRT3	5	62	mRNA
L2_3244_hg19_inputLibs_chr20_-_62103954	upregulated	KCNQ2	2	17	mRNA
L2_3244_hg19_inputLibs_chr20_+_17207683	upregulated	PCSK2	9	76	mRNA
L2_3244_hg19_inputLibs_chr20_+_33464414	upregulated	ACSS2	4	36	mRNA
L2_3244_hg19_inputLibs_chr20_+_53092237	upregulated	DOK5	1	11	mRNA
L2_3244_hg19_inputLibs_chr21_-_16438177	upregulated	.	1	10	.
L2_3244_hg19_inputLibs_chr21_-_42540714	upregulated	.	1	18	.
L2_3244_hg19_inputLibs_chr21_+_18984042	upregulated	.	3	25	.
L2_3244_hg19_inputLibs_chr21_+_27011747	upregulated	JAM2	6	110	mRNA
L2_3244_hg19_inputLibs_chr21_+_27011747	upregulated	JAM2	6	110	mRNA
L2_3244_hg19_inputLibs_chr22_-_31003027	upregulated	PES1	1	11	mRNA
L2_3244_hg19_inputLibs_chr22_-_43739378	upregulated	SCUBE1	1	15	mRNA
L2_3244_hg19_inputLibs_chr22_-_50746073	upregulated	PLXNB2	9	90	mRNA
L2_3244_hg19_inputLibs_chr3_-_112357172	upregulated	.	1	29	.
L2_3244_hg19_inputLibs_chr3_-_112359965	upregulated	CCDC80	4	132	mRNA
L2_3244_hg19_inputLibs_chr3_-_122746580	upregulated	SEMA5B	7	66	mRNA
L2_3244_hg19_inputLibs_chr3_-_123603160	upregulated	MYLK	4	36	mRNA
L2_3244_hg19_inputLibs_chr3_-_132345951	upregulated	.	1	14	.
L2_3244_hg19_inputLibs_chr3_-_165555187	upregulated	BCHE	1	16	mRNA
L2_3244_hg19_inputLibs_chr3_-_93692691	upregulated	PROS1	4	38	mRNA

L2_3244_hg19_inputLibs_chr3_+_111717777	upregulated	TAGLN3	2	26	mRNA
L2_3244_hg19_inputLibs_chr3_+_111718071	upregulated	TAGLN3	1	18	mRNA
L2_3244_hg19_inputLibs_chr3_+_111718182	upregulated	TAGLN3	3	30	mRNA
L2_3244_hg19_inputLibs_chr3_+_13590625	upregulated	FBLN2	3	41	mRNA
L2_3244_hg19_inputLibs_chr3_+_147127149	upregulated	ZIC1	1	39	mRNA
L2_3244_hg19_inputLibs_chr3_+_157154637	upregulated	PTX3	8	367	mRNA
L2_3244_hg19_inputLibs_chr3_+_2140610	upregulated	CNTN4	2	17	mRNA
L2_3244_hg19_inputLibs_chr3_+_85008138	upregulated	CADM2	17	189	mRNA
L2_3244_hg19_inputLibs_chr4_-_110223814	upregulated	COL25A1	1	28	mRNA
L2_3244_hg19_inputLibs_chr4_-_141677513	upregulated	TBC1D9	1	15	mRNA
L2_3244_hg19_inputLibs_chr4_-_143326587	upregulated	.	2	41	.
L2_3244_hg19_inputLibs_chr4_-_153273892	upregulated	FBXW7	1	10	mRNA
L2_3244_hg19_inputLibs_chr4_-_157892278	upregulated	PDGFC	9	103	mRNA
L2_3244_hg19_inputLibs_chr4_-_157892506	upregulated	PDGFC	4	222	mRNA
L2_3244_hg19_inputLibs_chr4_-_157892941	upregulated	PDGFC	4	50	mRNA
L2_3244_hg19_inputLibs_chr4_-_159093525	upregulated	FAM198B	1	10	mRNA
L2_3244_hg19_inputLibs_chr4_-_186733378	upregulated	SORBS2	2	54	mRNA
L2_3244_hg19_inputLibs_chr4_-_77870718	upregulated	.	1	15	.
L2_3244_hg19_inputLibs_chr4_-_83719925	upregulated	SCD5	1	26	mRNA
L2_3244_hg19_inputLibs_chr4_-_8442450	upregulated	ACOX3	2	28	mRNA
L2_3244_hg19_inputLibs_chr4_-_87281181	upregulated	MAPK10	1	24	mRNA
L2_3244_hg19_inputLibs_chr4_-_88450375	upregulated	SPARCL1	1	236	mRNA
L2_3244_hg19_inputLibs_chr4_+_100496044	upregulated	.	2	28	.
L2_3244_hg19_inputLibs_chr4_+_124317989	upregulated	SPRY1	1	15	mRNA
L2_3244_hg19_inputLibs_chr4_+_156680189	upregulated	GUCY1B3	2	72	mRNA
L2_3244_hg19_inputLibs_chr4_+_20254511	upregulated	.	2	25	.
L2_3244_hg19_inputLibs_chr4_+_3294725	upregulated	.	1	11	.
L2_3244_hg19_inputLibs_chr4_+_41614916	upregulated	LIMCH1	8	144	mRNA
L2_3244_hg19_inputLibs_chr4_+_62065939	upregulated	.	1	12	.
L2_3244_hg19_inputLibs_chr4_+_78078394	upregulated	CCNG2	2	25	mRNA
L2_3244_hg19_inputLibs_chr4_+_90800678	upregulated	.	1	34	.
L2_3244_hg19_inputLibs_chr5_-_100238943	upregulated	ST8SIA4	5	57	mRNA
L2_3244_hg19_inputLibs_chr5_-_111093072	upregulated	NREP	1	19	mRNA
L2_3244_hg19_inputLibs_chr5_-_141337767	upregulated	.	1	12	.
L2_3244_hg19_inputLibs_chr5_-_147162215	upregulated	JAKMIP2	2	34	mRNA
L2_3244_hg19_inputLibs_chr5_-_149535405	upregulated	PDGFRB	4	59	mRNA
L2_3244_hg19_inputLibs_chr5_-_38556712	upregulated	LIFR	9	198	mRNA
L2_3244_hg19_inputLibs_chr5_-_44389522	upregulated	.	1	47	.
L2_3244_hg19_inputLibs_chr5_-_58883451	upregulated	.	1	19	.
L2_3244_hg19_inputLibs_chr5_-_81046867	upregulated	SSBP2	8	121	mRNA
L2_3244_hg19_inputLibs_chr5_-_88179031	upregulated	MEF2C	1	13	mRNA
L2_3244_hg19_inputLibs_chr5_+_140571960	upregulated	PCDHB10	1	20	mRNA
L2_3244_hg19_inputLibs_chr5_+_31317991	upregulated	.	1	11	.
L2_3244_hg19_inputLibs_chr5_+_71479308	upregulated	.	3	82	.
L2_3244_hg19_inputLibs_chr5_+_79287162	upregulated	.	1	10	.
L2_3244_hg19_inputLibs_chr5_+_92918960	upregulated	NR2F1	2	148	mRNA
L2_3244_hg19_inputLibs_chr5_+_95066866	upregulated	RHOBTB3	1	12	mRNA
L2_3244_hg19_inputLibs_chr5_+_95067066	upregulated	RHOBTB3	4	35	mRNA
L2_3244_hg19_inputLibs_chr6_-_11044565	upregulated	ELOVL2	1	15	mRNA

L2_3244_hg19_inputLibs_chr6_-_112575769	upregulated	LAMA4	9	735	mRNA
L2_3244_hg19_inputLibs_chr6_-_127840447	upregulated	SOGA3	2	25	mRNA
L2_3244_hg19_inputLibs_chr6_-_132722614	upregulated	MOXD1	2	32	mRNA
L2_3244_hg19_inputLibs_chr6_-_144329521	upregulated	PLAGL1	1	14	mRNA
L2_3244_hg19_inputLibs_chr6_-_170600016	upregulated	DLL1	1	23	mRNA
L2_3244_hg19_inputLibs_chr6_-_24911321	upregulated	FAM65B	4	76	mRNA
L2_3244_hg19_inputLibs_chr6_-_48078930	upregulated	.	2	18	.
L2_3244_hg19_inputLibs_chr6_-_55740388	upregulated	BMP5	1	37	mRNA
L2_3244_hg19_inputLibs_chr6_+_113201354	upregulated	.	2	59	.
L2_3244_hg19_inputLibs_chr6_+_114180427	upregulated	.	1	17	.
L2_3244_hg19_inputLibs_chr6_+_116832831	upregulated	FAM26E	4	40	mRNA
L2_3244_hg19_inputLibs_chr6_+_123100890	upregulated	FABP7	2	103	mRNA
L2_3244_hg19_inputLibs_chr6_+_33175142	upregulated	.	1	13	.
L2_3244_hg19_inputLibs_chr6_+_36644238	upregulated	CDKN1A	1	20	mRNA
L2_3244_hg19_inputLibs_chr6_+_36646489	upregulated	CDKN1A	4	47	mRNA
L2_3244_hg19_inputLibs_chr6_+_50786513	upregulated	TFAP2B	1	317	mRNA
L2_3244_hg19_inputLibs_chr6_+_86159809	upregulated	NT5E	2	22	mRNA
L2_3244_hg19_inputLibs_chr6_+_89827762	upregulated	.	1	18	.
L2_3244_hg19_inputLibs_chr6_+_99282447	upregulated	POU3F2	1	38	mRNA
L2_3244_hg19_inputLibs_chr7_-_104909497	upregulated	SRPK2	5	55	mRNA
L2_3244_hg19_inputLibs_chr7_-_136938342	upregulated	.	4	33	.
L2_3244_hg19_inputLibs_chr7_-_136939691	upregulated	.	4	43	.
L2_3244_hg19_inputLibs_chr7_-_137028403	upregulated	PTN	20	266	mRNA
L2_3244_hg19_inputLibs_chr7_-_28997923	upregulated	TRIL	1	19	mRNA
L2_3244_hg19_inputLibs_chr7_-_28998248	upregulated	TRIL	4	129	mRNA
L2_3244_hg19_inputLibs_chr7_-_45960871	upregulated	IGFBP3	1	17	mRNA
L2_3244_hg19_inputLibs_chr7_-_84816380	upregulated	.	1	10	.
L2_3244_hg19_inputLibs_chr7_-_99869824	upregulated	GATS	1	15	mRNA
L2_3244_hg19_inputLibs_chr7_+_130131983	upregulated	MEST	55	684	mRNA
L2_3244_hg19_inputLibs_chr7_+_20371081	upregulated	ITGB8	1	12	mRNA
L2_3244_hg19_inputLibs_chr7_+_90893766	upregulated	FZD1	7	58	mRNA
L2_3244_hg19_inputLibs_chr7_+_93551045	upregulated	GNG11	48	673	mRNA
L2_3244_hg19_inputLibs_chr7_+_93551364	upregulated	GNG11	13	252	mRNA
L2_3244_hg19_inputLibs_chr7_+_94285680	upregulated	PEG10	62	500	mRNA
L2_3244_hg19_inputLibs_chr7_+_94293003	upregulated	.	10	85	.
L2_3244_hg19_inputLibs_chr7_+_94294095	upregulated	.	3	55	.
L2_3244_hg19_inputLibs_chr8_-_102803189	upregulated	NCALD	13	438	mRNA
L2_3244_hg19_inputLibs_chr8_-_120685615	upregulated	.	1	61	.
L2_3244_hg19_inputLibs_chr8_-_124286507	upregulated	ZHX1	7	61	mRNA
L2_3244_hg19_inputLibs_chr8_-_124286507	upregulated	ZHX1-C8ORF76	7	61	mRNA
L2_3244_hg19_inputLibs_chr8_-_125740714	upregulated	MTSS1	8	82	mRNA
L2_3244_hg19_inputLibs_chr8_-_12990793	upregulated	DLC1	3	26	mRNA
L2_3244_hg19_inputLibs_chr8_-_24814126	upregulated	NEFL	20	291	mRNA
L2_3244_hg19_inputLibs_chr8_-_40755343	upregulated	ZMAT4	1	27	mRNA
L2_3244_hg19_inputLibs_chr8_-_54164134	upregulated	OPRK1	1	15	mRNA
L2_3244_hg19_inputLibs_chr8_-_61193957	upregulated	CA8	11	100	mRNA
L2_3244_hg19_inputLibs_chr8_-_65281251	upregulated	.	1	23	.
L2_3244_hg19_inputLibs_chr8_+_104152977	upregulated	BAALC	2	239	mRNA
L2_3244_hg19_inputLibs_chr8_+_107738333	upregulated	OXR1	2	23	mRNA

L2_3244_hg19_inputLibs_chr8_+_24771272	upregulated	NEFM	6	84	mRNA
L2_3244_hg19_inputLibs_chr8_+_24774993	upregulated	.	1	12	.
L2_3244_hg19_inputLibs_chr8_+_38089147	upregulated	DDHD2	2	20	mRNA
L2_3244_hg19_inputLibs_chr8_+_54792637	upregulated	.	1	18	.
L2_3244_hg19_inputLibs_chr8_+_54793430	upregulated	RGS20	2	43	mRNA
L2_3244_hg19_inputLibs_chr8_+_97505880	upregulated	SDC2	3	35	mRNA
L2_3244_hg19_inputLibs_chr8_+_97506477	upregulated	SDC2	20	161	mRNA
L2_3244_hg19_inputLibs_chr9_-_107690436	upregulated	ABCA1	3	65	mRNA
L2_3244_hg19_inputLibs_chr9_-_130533572	upregulated	SH2D3C	1	14	mRNA
L2_3244_hg19_inputLibs_chr9_-_23821818	upregulated	ELAVL2	1	19	mRNA
L2_3244_hg19_inputLibs_chr9_-_34589760	upregulated	CNTFR	2	17	mRNA
L2_3244_hg19_inputLibs_chr9_-_38424458	upregulated	IGFBPL1	21	264	mRNA
L2_3244_hg19_inputLibs_chr9_+_112887791	upregulated	AKAP2	4	34	mRNA
L2_3244_hg19_inputLibs_chr9_+_133884559	upregulated	LAMC3	3	42	mRNA
L2_3244_hg19_inputLibs_chr9_+_2158468	upregulated	.	2	123	.
L2_3244_hg19_inputLibs_chr9_+_2622130	upregulated	VLDLR	2	23	mRNA
L2_3244_hg19_inputLibs_chr9_+_71320150	upregulated	PIP5K1B	3	33	mRNA
L2_3244_hg19_inputLibs_chr9_+_71320621	upregulated	PIP5K1B	1	39	mRNA
L2_3244_hg19_inputLibs_chr9_+_91606381	upregulated	S1PR3	2	63	mRNA
L2_3244_hg19_inputLibs_chr9_+_92219925	upregulated	GADD45G	1	17	mRNA
L2_3244_hg19_inputLibs_chrX_-_10645766	upregulated	MID1	6	49	mRNA
L2_3244_hg19_inputLibs_chrX_-_128788933	upregulated	APLN	46	717	mRNA
L2_3244_hg19_inputLibs_chrX_-_139590219	upregulated	.	1	11	.
L2_3244_hg19_inputLibs_chrX_-_76234925	upregulated	.	1	68	.
L2_3244_hg19_inputLibs_chrX_-_99665271	upregulated	PCDH19	2	22	mRNA
L2_3244_hg19_inputLibs_chrX_+_152760438	upregulated	BGN	20	1678	mRNA
L2_3244_hg19_inputLibs_chrX_+_152770156	upregulated	.	1	44	.
