# Supplementary material for: Genome-Wide Definition of Promoter and Enhancer Usage during Neural Induction of Human Embryonic Stem Cells
Source: PLoS One. 2015 May 15;10(5):e0126590. doi: 10.1371/journal.pone.0126590 (PMC4433211; doi:10.1371/journal.pone.0126590)
Supplement: S4 Table — (PDF) [file pone.0126590.s014.pdf]

TABLE S4

Table of CAGE promoters characterized by epigenetic profile of active promoter (in a window of ± 2 kb from CAGE-promoter ends)

Active promoters:

- $H3K4me3^{+}/H3K4me1^{-}/H3K27me3^{-}$ or
- $H3K4me3^{high}/H3K4me1^{low}/H3K27me3^{-}$

Poised promoters:

- $H3K4me3^{+}/H3K4me1^{-}/H3K27me3^{+}$ or
- $H3K4me3^{high}/H3K4me1^{low}/H3K27me3^{+}$

ESC CAGE promoters	TOT promoters	ACTIVE	POISED	TOT (H3K4me3+)	% TOT (H3K4me3+)
**All**	13730	8577	2206	10783	79%
**protein-coding**	10571	7388	1906	9294	88%
**non-coding**	319	225	34	259	81%
**un-annotated**	2840	964	266	1230	43%

NESC CAGE promoters	TOT promoters	ACTIVE	POISED in ESCs	TOT (H3K4me3+)	% TOT (H3K4me3+)
**All**	13569	8136	2003	10139	75%
**protein-coding**	10466	6964	1686	8650	83%
**non-coding**	323	224	37	261	81%
**un-annotated**	2780	948	280	1228	44%
