# Supplementary material for: Genome-Wide Definition of Promoter and Enhancer Usage during Neural Induction of Human Embryonic Stem Cells
Source: PLoS One. 2015 May 15;10(5):e0126590. doi: 10.1371/journal.pone.0126590 (PMC4433211; doi:10.1371/journal.pone.0126590)
Supplement: S5 Table — (PDF) [file pone.0126590.s015.pdf]

TABLE S5

Table of CAGE promoters that transit from a poised to a transcriptionally active state during neural commitment of ESCs.

CAGEchr: genomic coordinate of CAGE promoter (chromosome)
CAGEstart: genomic coordinate of CAGE promoter (start)
CAGEend: genomic coordinate of CAGE promoter (end)
CAGEstrand: genomic coordinate of CAGE promoter (strand)
CAGE_ID: univocal identification code of CAGE promoter
Prom_Type: classification of CAGE promoter on the basis of its activity level
TMP ESCs: TPM (Tags Per Million) determined by CAGE-seq in ESCs
TPM NESCs: TPM (Tags Per Million) determined by CAGE-seq in NESCs

CAGE chr	CAGE start	CAGE end	CAGE strand	CAGE_ID	Prom_Type	TPM ESCs	TPM NESCs
chr2	105470483	105470865	+	L2_3244_hg19_inputLibs_chr2_+_105470541	NESC-spec	0	259
chr8	19796720	19796867	+	L2_3244_hg19_inputLibs_chr8_+_19796765	NESC-spec	0	171
chr2	105467871	105467942	-	L2_3244_hg19_inputLibs_chr2_-_105467911	NESC-spec	0	149
chr7	157483628	157483694	-	L2_3244_hg19_inputLibs_chr7_-_157483658	NESC-spec	0	106
chr5	136834904	136835072	-	L2_3244_hg19_inputLibs_chr5_-_136834999	NESC-spec	0	90
chr18	49866493	49866594	+	L2_3244_hg19_inputLibs_chr18_+_49866565	NESC-spec	0	65
chr17	15168584	15168646	-	L2_3244_hg19_inputLibs_chr17_-_15168643	NESC-spec	0	63
chr4	176923484	176923599	-	L2_3244_hg19_inputLibs_chr4_-_176923564	NESC-spec	0	49
chr3	47620271	47620407	-	L2_3244_hg19_inputLibs_chr3_-_47620355	NESC-spec	0	48
chr12	48398383	48398479	-	L2_3244_hg19_inputLibs_chr12_-_48398435	NESC-spec	0	37
chr9	113800675	113800871	-	L2_3244_hg19_inputLibs_chr9_-_113800750	NESC-spec	0	32
chr2	105488791	105488866	-	L2_3244_hg19_inputLibs_chr2_-_105488816	NESC-spec	0	31
chr7	19157232	19157301	-	L2_3244_hg19_inputLibs_chr7_-_19157260	NESC-spec	0	30
chr13	88323392	88323556	-	L2_3244_hg19_inputLibs_chr13_-_88323423	NESC-spec	0	26
chr5	87969107	87969165	-	L2_3244_hg19_inputLibs_chr5_-_87969136	NESC-spec	0	25
chr11	60680194	60680298	+	L2_3244_hg19_inputLibs_chr11_+_60680203	NESC-spec	0	23
chr5	92919033	92919179	+	L2_3244_hg19_inputLibs_chr5_+_92919129	NESC-spec	0	23
chr15	53082502	53082594	-	L2_3244_hg19_inputLibs_chr15_-_53082533	NESC-spec	0	22
chr18	49867009	49867173	+	L2_3244_hg19_inputLibs_chr18_+_49867049	NESC-spec	0	22
chr2	105469697	105469836	+	L2_3244_hg19_inputLibs_chr2_+_105469743	NESC-spec	0	22
chr12	48398089	48398217	-	L2_3244_hg19_inputLibs_chr12_-_48398111	NESC-spec	0	21
chr3	89156769	89156896	+	L2_3244_hg19_inputLibs_chr3_+_89156839	NESC-spec	0	20

chr5	111755804	111755885	-	L2_3244_hg19_inputLibs_chr5_-_111755831	NESC-spec	0	20
chr12	20521437	20521504	+	L2_3244_hg19_inputLibs_chr12_+_20521480	NESC-spec	0	19
chr18	49866614	49866866	+	L2_3244_hg19_inputLibs_chr18_+_49866638	NESC-spec	0	18
chr8	124428557	124428666	-	L2_3244_hg19_inputLibs_chr8_-_124428598	NESC-spec	0	16
chr10	48439106	48439204	-	L2_3244_hg19_inputLibs_chr10_-_48439106	NESC-spec	0	15
chr3	79817102	79817231	-	L2_3244_hg19_inputLibs_chr3_-_79817171	NESC-spec	0	15
chr2	14772786	14772868	+	L2_3244_hg19_inputLibs_chr2_+_14772839	NESC-spec	0	14
chr3	157154373	157154409	+	L2_3244_hg19_inputLibs_chr3_+_157154397	NESC-spec	0	14
chr4	81951780	81951851	+	L2_3244_hg19_inputLibs_chr4_+_81951811	NESC-spec	0	14
chr7	86273829	86273962	+	L2_3244_hg19_inputLibs_chr7_+_86273904	NESC-spec	0	14
chr17	14204323	14204459	+	L2_3244_hg19_inputLibs_chr17_+_14204403	NESC-spec	0	13
chr20	20348745	20348749	+	L2_3244_hg19_inputLibs_chr20_+_20348748	NESC-spec	0	13
chr5	92918755	92918798	+	L2_3244_hg19_inputLibs_chr5_+_92918793	NESC-spec	0	13
chr7	86273186	86273242	+	L2_3244_hg19_inputLibs_chr7_+_86273232	NESC-spec	0	13
chr10	92922684	92922814	+	L2_3244_hg19_inputLibs_chr10_+_92922748	NESC-spec	0	12
chr12	58026904	58027004	-	L2_3244_hg19_inputLibs_chr12_-_58026982	NESC-spec	0	12
chr13	33924764	33924874	-	L2_3244_hg19_inputLibs_chr13_-_33924817	NESC-spec	0	12
chr15	48009550	48009690	+	L2_3244_hg19_inputLibs_chr15_+_48009662	NESC-spec	0	12
chr17	33700632	33700650	-	L2_3244_hg19_inputLibs_chr17_-_33700640	NESC-spec	0	12
chr17	48207471	48207569	-	L2_3244_hg19_inputLibs_chr17_-_48207536	NESC-spec	0	12
chr2	105469440	105469502	+	L2_3244_hg19_inputLibs_chr2_+_105469494	NESC-spec	0	12
chr4	52917442	52917596	+	L2_3244_hg19_inputLibs_chr4_+_52917583	NESC-spec	0	12
chr5	92957164	92957259	-	L2_3244_hg19_inputLibs_chr5_-_92957174	NESC-spec	0	12
chr7	150945639	150945796	-	L2_3244_hg19_inputLibs_chr7_-_150945743	NESC-spec	0	12
chr8	95961553	95961649	-	L2_3244_hg19_inputLibs_chr8_-_95961587	NESC-spec	0	12
chr1	51434446	51434510	+	L2_3244_hg19_inputLibs_chr1_+_51434477	NESC-spec	0	11
chr10	120967234	120967286	+	L2_3244_hg19_inputLibs_chr10_+_120967253	NESC-spec	0	11
chr11	113930405	113930482	+	L2_3244_hg19_inputLibs_chr11_+_113930464	NESC-spec	0	11
chr11	118478283	118478361	+	L2_3244_hg19_inputLibs_chr11_+_118478318	NESC-spec	0	11
chr14	51027800	51027897	-	L2_3244_hg19_inputLibs_chr14_-_51027845	NESC-spec	0	11
chr14	70346076	70346136	+	L2_3244_hg19_inputLibs_chr14_+_70346131	NESC-spec	0	11
chr18	7117978	7118069	-	L2_3244_hg19_inputLibs_chr18_-_7117993	NESC-spec	0	11
chr2	112895919	112896003	+	L2_3244_hg19_inputLibs_chr2_+_112895991	NESC-spec	0	11

chr4	96470124	96470195	-	L2_3244_hg19_inputLibs_chr4_-_96470124	NESC-spec	0	11
chr6	112575913	112575973	-	L2_3244_hg19_inputLibs_chr6_-_112575924	NESC-spec	0	11
chr8	54792924	54793016	+	L2_3244_hg19_inputLibs_chr8_+_54792977	NESC-spec	0	11
chr8	65281090	65281171	-	L2_3244_hg19_inputLibs_chr8_-_65281146	NESC-spec	0	11
chr10	92922565	92922663	+	L2_3244_hg19_inputLibs_chr10_+_92922613	NESC-spec	0	10
chr2	68546493	68547119	-	L2_3244_hg19_inputLibs_chr2_-_68546548	upregulated_promoter	1	49
chr5	44389472	44389576	-	L2_3244_hg19_inputLibs_chr5_-_44389522	upregulated_promoter	1	47
chr9	71320548	71320652	+	L2_3244_hg19_inputLibs_chr9_+_71320621	upregulated_promoter	1	39
chr13	36705449	36705520	-	L2_3244_hg19_inputLibs_chr13_-_36705467	upregulated_promoter	1	29
chr18	500627	500751	-	L2_3244_hg19_inputLibs_chr18_-_500698	upregulated_promoter	1	28
chr4	83719797	83720085	-	L2_3244_hg19_inputLibs_chr4_-_83719925	upregulated_promoter	1	26
chr6	170600013	170600018	-	L2_3244_hg19_inputLibs_chr6_-_170600016	upregulated_promoter	1	23
chr8	65281190	65281318	-	L2_3244_hg19_inputLibs_chr8_-_65281251	upregulated_promoter	1	23
chr16	79632947	79633103	-	L2_3244_hg19_inputLibs_chr16_-_79633102	upregulated_promoter	1	21
chr12	106976642	106976720	+	L2_3244_hg19_inputLibs_chr12_+_106976683	upregulated_promoter	1	20
chr10	63661402	63661463	+	L2_3244_hg19_inputLibs_chr10_+_63661456	upregulated_promoter	1	19
chr2	230579157	230579288	-	L2_3244_hg19_inputLibs_chr2_-_230579245	upregulated_promoter	1	19
chr7	28997878	28997943	-	L2_3244_hg19_inputLibs_chr7_-_28997923	upregulated_promoter	1	19
chr9	23821785	23821864	-	L2_3244_hg19_inputLibs_chr9_-_23821818	upregulated_promoter	1	19
chr21	42540709	42540755	-	L2_3244_hg19_inputLibs_chr21_-_42540714	upregulated_promoter	1	18
chr6	89827753	89827764	+	L2_3244_hg19_inputLibs_chr6_+_89827762	upregulated_promoter	1	18
chr8	54792474	54792729	+	L2_3244_hg19_inputLibs_chr8_+_54792637	upregulated_promoter	1	18
chr12	48577379	48577466	+	L2_3244_hg19_inputLibs_chr12_+_48577410	upregulated_promoter	1	17
chr17	48207312	48207444	-	L2_3244_hg19_inputLibs_chr17_-_48207435	upregulated_promoter	1	17
chr6	114180278	114180491	+	L2_3244_hg19_inputLibs_chr6_+_114180427	upregulated_promoter	1	17
chr7	45960860	45960929	-	L2_3244_hg19_inputLibs_chr7_-_45960871	upregulated_promoter	1	17
chr9	92219924	92219929	+	L2_3244_hg19_inputLibs_chr9_+_92219925	upregulated_promoter	1	17
chr4	77870695	77870796	-	L2_3244_hg19_inputLibs_chr4_-_77870718	upregulated_promoter	1	15
chr4	141677404	141677596	-	L2_3244_hg19_inputLibs_chr4_-_141677513	upregulated_promoter	1	15
chr6	11044490	11044764	-	L2_3244_hg19_inputLibs_chr6_-_11044565	upregulated_promoter	1	15
chr8	54164092	54164212	-	L2_3244_hg19_inputLibs_chr8_-_54164134	upregulated_promoter	1	15
chr6	144329299	144329599	-	L2_3244_hg19_inputLibs_chr6_-_144329521	upregulated_promoter	1	14
chr5	88178852	88179129	-	L2_3244_hg19_inputLibs_chr5_-_88179031	upregulated_promoter	1	13

chr6	33175079	33175189	+	L2_3244_hg19_inputLibs_chr6_+_33175142	upregulated_promoter	1	13
chr12	20522171	20522251	+	L2_3244_hg19_inputLibs_chr12_+_20522212	upregulated_promoter	1	12
chr12	64238457	64238628	+	L2_3244_hg19_inputLibs_chr12_+_64238479	upregulated_promoter	1	12
chr15	79103674	79103750	-	L2_3244_hg19_inputLibs_chr15_-_79103697	upregulated_promoter	1	12
chr18	67068073	67068178	+	L2_3244_hg19_inputLibs_chr18_+_67068101	upregulated_promoter	1	12
chr4	62065839	62065958	+	L2_3244_hg19_inputLibs_chr4_+_62065939	upregulated_promoter	1	12
chr5	95066827	95066898	+	L2_3244_hg19_inputLibs_chr5_+_95066866	upregulated_promoter	1	12
chr7	20370953	20371193	+	L2_3244_hg19_inputLibs_chr7_+_20371081	upregulated_promoter	1	12
chr8	24774934	24775075	+	L2_3244_hg19_inputLibs_chr8_+_24774993	upregulated_promoter	1	12
chr1	101702567	101702618	+	L2_3244_hg19_inputLibs_chr1_+_101702573	upregulated_promoter	1	11
chr1	151119074	151119146	-	L2_3244_hg19_inputLibs_chr1_-_151119143	upregulated_promoter	1	11
chr10	13043736	13043809	-	L2_3244_hg19_inputLibs_chr10_-_13043756	upregulated_promoter	1	11
chr11	65325625	65325864	-	L2_3244_hg19_inputLibs_chr11_-_65325712	upregulated_promoter	1	11
chr20	53092139	53092254	+	L2_3244_hg19_inputLibs_chr20_+_53092237	upregulated_promoter	1	11
chr4	3294719	3294799	+	L2_3244_hg19_inputLibs_chr4_+_3294725	upregulated_promoter	1	11
chrX	139590213	139590243	-	L2_3244_hg19_inputLibs_chrX_-_139590219	upregulated_promoter	1	11
chr13	40177426	40177528	-	L2_3244_hg19_inputLibs_chr13_-_40177473	upregulated_promoter	1	10
chr16	54320098	54320215	-	L2_3244_hg19_inputLibs_chr16_-_54320117	upregulated_promoter	1	10
chr2	182322065	182322160	+	L2_3244_hg19_inputLibs_chr2_+_182322112	upregulated_promoter	1	10
chr21	16438162	16438345	-	L2_3244_hg19_inputLibs_chr21_-_16438177	upregulated_promoter	1	10
chr7	84816372	84816447	-	L2_3244_hg19_inputLibs_chr7_-_84816380	upregulated_promoter	1	10
chr8	104152875	104153154	+	L2_3244_hg19_inputLibs_chr8_+_104152977	upregulated_promoter	2	239
chr5	92918863	92918998	+	L2_3244_hg19_inputLibs_chr5_+_92918960	upregulated_promoter	2	148
chr12	20522565	20522688	+	L2_3244_hg19_inputLibs_chr12_+_20522628	upregulated_promoter	2	91
chr4	156680114	156680263	+	L2_3244_hg19_inputLibs_chr4_+_156680189	upregulated_promoter	2	72
chr9	91606317	91606414	+	L2_3244_hg19_inputLibs_chr9_+_91606381	upregulated_promoter	2	63
chr8	54793398	54793655	+	L2_3244_hg19_inputLibs_chr8_+_54793430	upregulated_promoter	2	43
chr10	50323528	50323599	-	L2_3244_hg19_inputLibs_chr10_-_50323554	upregulated_promoter	2	41
chr13	95364931	95364999	+	L2_3244_hg19_inputLibs_chr13_+_95364967	upregulated_promoter	2	37
chr6	132722577	132722687	-	L2_3244_hg19_inputLibs_chr6_-_132722614	upregulated_promoter	2	32
chr11	47615842	47616002	-	L2_3244_hg19_inputLibs_chr11_-_47615935	upregulated_promoter	2	29
chr2	183731765	183731918	-	L2_3244_hg19_inputLibs_chr2_-_183731890	upregulated_promoter	2	27
chr12	56136866	56136909	+	L2_3244_hg19_inputLibs_chr12_+_56136904	upregulated_promoter	2	26

chr4	20254500	20254515	+	L2_3244_hg19_inputLibs_chr4_+_20254511	upregulated_promoter	2	25
chr10	21806736	21806775	-	L2_3244_hg19_inputLibs_chr10_-_21806761	upregulated_promoter	2	24
chr17	48207118	48207190	-	L2_3244_hg19_inputLibs_chr17_-_48207171	upregulated_promoter	2	23
chr2	66662442	66662660	+	L2_3244_hg19_inputLibs_chr2_+_66662514	upregulated_promoter	2	23
chr10	111967301	111967444	+	L2_3244_hg19_inputLibs_chr10_+_111967361	upregulated_promoter	2	22
chr6	86159671	86160067	+	L2_3244_hg19_inputLibs_chr6_+_86159809	upregulated_promoter	2	22
chrX	99665263	99665285	-	L2_3244_hg19_inputLibs_chrX_-_99665271	upregulated_promoter	2	22
chr14	85996322	85996625	+	L2_3244_hg19_inputLibs_chr14_+_85996486	upregulated_promoter	2	21
chr10	118031651	118031877	-	L2_3244_hg19_inputLibs_chr10_-_118031849	upregulated_promoter	2	20
chr8	38089084	38089351	+	L2_3244_hg19_inputLibs_chr8_+_38089147	upregulated_promoter	2	20
chr16	79634567	79634673	-	L2_3244_hg19_inputLibs_chr16_-_79634631	upregulated_promoter	2	19
chr17	57408891	57409115	+	L2_3244_hg19_inputLibs_chr17_+_57409014	upregulated_promoter	2	19
chr10	118032113	118032261	-	L2_3244_hg19_inputLibs_chr10_-_118032176	upregulated_promoter	2	17
chr3	2140539	2140632	+	L2_3244_hg19_inputLibs_chr3_+_2140610	upregulated_promoter	2	17
chr9	34589691	34589788	-	L2_3244_hg19_inputLibs_chr9_-_34589760	upregulated_promoter	2	17
chr10	134000385	134000487	+	L2_3244_hg19_inputLibs_chr10_+_134000452	upregulated_promoter	2	16
chr12	48398242	48398344	-	L2_3244_hg19_inputLibs_chr12_-_48398259	upregulated_promoter	3	345
chr13	111767474	111767707	+	L2_3244_hg19_inputLibs_chr13_+_111767677	upregulated_promoter	3	71
chr9	107690334	107690548	-	L2_3244_hg19_inputLibs_chr9_-_107690436	upregulated_promoter	3	65
chr13	58204230	58204266	+	L2_3244_hg19_inputLibs_chr13_+_58204240	upregulated_promoter	3	59
chr17	15165790	15165949	-	L2_3244_hg19_inputLibs_chr17_-_15165833	upregulated_promoter	3	43
chr19	41103068	41103145	+	L2_3244_hg19_inputLibs_chr19_+_41103116	upregulated_promoter	3	34
chr9	71320104	71320216	+	L2_3244_hg19_inputLibs_chr9_+_71320150	upregulated_promoter	3	33
chr1	94703054	94703193	-	L2_3244_hg19_inputLibs_chr1_-_94703140	upregulated_promoter	3	27
chr15	80696607	80696746	+	L2_3244_hg19_inputLibs_chr15_+_80696738	upregulated_promoter	3	27
chr8	12990732	12990804	-	L2_3244_hg19_inputLibs_chr8_-_12990793	upregulated_promoter	3	26
chr4	157892418	157892567	-	L2_3244_hg19_inputLibs_chr4_-_157892506	upregulated_promoter	4	222
chr7	28998109	28998378	-	L2_3244_hg19_inputLibs_chr7_-_28998248	upregulated_promoter	4	129
chr13	67804378	67804516	-	L2_3244_hg19_inputLibs_chr13_-_67804468	upregulated_promoter	4	79
chr11	128392069	128392347	-	L2_3244_hg19_inputLibs_chr11_-_128392205	upregulated_promoter	4	74
chr17	70117124	70117272	+	L2_3244_hg19_inputLibs_chr17_+_70117159	upregulated_promoter	4	70
chr2	39892915	39893235	+	L2_3244_hg19_inputLibs_chr2_+_39893095	upregulated_promoter	4	66
chr4	157892681	157893115	-	L2_3244_hg19_inputLibs_chr4_-_157892941	upregulated_promoter	4	50

chr19	36359410	36359543	+	L2_3244_hg19_inputLibs_chr19_+_36359440	upregulated_promoter	4	46
chr11	94964164	94964430	-	L2_3244_hg19_inputLibs_chr11_-_94964364	upregulated_promoter	4	38
chr16	54320517	54320753	-	L2_3244_hg19_inputLibs_chr16_-_54320667	upregulated_promoter	4	38
chr3	93692595	93692831	-	L2_3244_hg19_inputLibs_chr3_-_93692691	upregulated_promoter	4	38
chr5	95066973	95067137	+	L2_3244_hg19_inputLibs_chr5_+_95067066	upregulated_promoter	4	35
chr11	86511520	86511662	+	L2_3244_hg19_inputLibs_chr11_+_86511584	upregulated_promoter	5	66
chr13	95364767	95364855	-	L2_3244_hg19_inputLibs_chr13_-_95364790	upregulated_promoter	5	44
chr16	8768413	8768533	+	L2_3244_hg19_inputLibs_chr16_+_8768473	upregulated_promoter	5	42
chr16	52580951	52581107	-	L2_3244_hg19_inputLibs_chr16_-_52581034	upregulated_promoter	5	42
chr21	27011512	27011799	+	L2_3244_hg19_inputLibs_chr21_+_27011747	upregulated_promoter	6	110
chr8	24771209	24771287	+	L2_3244_hg19_inputLibs_chr8_+_24771272	upregulated_promoter	6	84
chr12	48357313	48357471	+	L2_3244_hg19_inputLibs_chr12_+_48357388	upregulated_promoter	6	59
chr3	122746529	122746651	-	L2_3244_hg19_inputLibs_chr3_-_122746580	upregulated_promoter	7	66
chr8	124286385	124286903	-	L2_3244_hg19_inputLibs_chr8_-_124286507	upregulated_promoter	7	61
chr7	90893703	90893985	+	L2_3244_hg19_inputLibs_chr7_+_90893766	upregulated_promoter	7	58
chr3	157154538	157154825	+	L2_3244_hg19_inputLibs_chr3_+_157154637	upregulated_promoter	8	367
chr5	81046816	81046999	-	L2_3244_hg19_inputLibs_chr5_-_81046867	upregulated_promoter	8	121
chr2	183731277	183731448	-	L2_3244_hg19_inputLibs_chr2_-_183731317	upregulated_promoter	8	105
chr8	125740641	125740955	-	L2_3244_hg19_inputLibs_chr8_-_125740714	upregulated_promoter	8	82
chr6	112575660	112575858	-	L2_3244_hg19_inputLibs_chr6_-_112575769	upregulated_promoter	9	735
chr4	157892059	157892399	-	L2_3244_hg19_inputLibs_chr4_-_157892278	upregulated_promoter	9	103
chr22	50746005	50746125	-	L2_3244_hg19_inputLibs_chr22_-_50746073	upregulated_promoter	9	90
chr20	17207624	17207706	+	L2_3244_hg19_inputLibs_chr20_+_17207683	upregulated_promoter	9	76
chr8	61193906	61194023	-	L2_3244_hg19_inputLibs_chr8_-_61193957	upregulated_promoter	11	100
chr14	52535697	52535845	-	L2_3244_hg19_inputLibs_chr14_-_52535776	upregulated_promoter	17	162
chr8	24814116	24814250	-	L2_3244_hg19_inputLibs_chr8_-_24814126	upregulated_promoter	20	291
chr12	106976743	106976850	+	L2_3244_hg19_inputLibs_chr12_+_106976779	upregulated_promoter	23	266
chr1	236228349	236228548	-	L2_3244_hg19_inputLibs_chr1_-_236228390	upregulated_promoter	42	460
chrX	128788877	128789012	-	L2_3244_hg19_inputLibs_chrX_-_128788933	upregulated_promoter	46	717
