# Supplementary material for: Genome-Wide Definition of Promoter and Enhancer Usage during Neural Induction of Human Embryonic Stem Cells
Source: PLoS One. 2015 May 15;10(5):e0126590. doi: 10.1371/journal.pone.0126590 (PMC4433211; doi:10.1371/journal.pone.0126590)
Supplement: S6 Table — (PDF) [file pone.0126590.s016.pdf]

TABLE S6

Table of CAGE-enhancers expressed in ESCs and NESCs.

Chr.: genomic coordinate of CAGE enhancer (chromosome)
start: genomic coordinate of CAGE enhancer (start)
end: genomic coordinate of CAGE enhancer (end)
TMP ESCs: TPM (Tags Per Million) determined by CAGE-seq in ESCs
TPM NESCs: TPM (Tags Per Million) determined by CAGE-seq in NESCs
p value: p value as determined by EdgeR

Chr.	start	end	TPM ESCs	TPM NESCs	p value
chr1	1549579	1549898	2	0	0.5215311
chr1	1840523	1840802	0	2	0.5215311
chr1	3387817	3388189	4	1	0.276758318
chr1	7764605	7764930	2	11	0.181027085
chr1	8472306	8472514	0	1	1
chr1	8772776	8772966	0	1	1
chr1	9987372	9987723	0	2	0.5215311
chr1	10269885	10270090	2	3	1
chr1	11791895	11792022	0	1	1
chr1	11968981	11969095	1	0	1
chr1	15930032	15930245	2	9	0.130835707
chr1	16126756	16126932	1	0	1
chr1	16175899	16176166	3	2	1
chr1	16176618	16176981	0	1	1
chr1	16339361	16339539	5	0	0.053865713
chr1	16481494	16481843	0	3	0.282296651
chr1	16694229	16694406	0	1	1
chr1	16825721	16825927	2	11	0.181027085
chr1	16840784	16841175	1	6	0.276758318
chr1	17231259	17231698	5	25	0.041616251
chr1	24648114	24648495	2	0	0.5215311
chr1	25566369	25566676	2	2	1
chr1	25756651	25756834	2	1	1
chr1	26827446	26827656	3	2	1
chr1	26947124	26947625	7	4	0.324634247
chr1	26947923	26948272	2	0	0.5215311
chr1	27024676	27025123	4	1	0.276758318
chr1	27113442	27113867	3	0	0.282296651
chr1	27337576	27337827	3	0	0.282296651
chr1	27852093	27852307	1	0	1
chr1	28845432	28845616	0	1	1
chr1	29214783	29215127	1	1	1
chr1	31627764	31628022	3	16	0.083265755
chr1	32409978	32410283	1	1	1
chr1	32816971	32817288	0	11	0.005401553
chr1	33077810	33077997	5	0	0.053865713
chr1	33592839	33593271	0	6	0.091014481
chr1	33813315	33813531	0	5	0.157936893

chr1	36555579	36555622	1	0	1
chr1	37942895	37943266	1	0	1
chr1	39571533	39571769	3	3	1
chr1	42127837	42128121	0	2	0.5215311
chr1	42344342	42344354	1	1	1
chr1	43390565	43390824	0	4	0.157936893
chr1	43586174	43586229	3	3	1
chr1	44495687	44496119	0	4	0.157936893
chr1	53791753	53792125	2	4	1
chr1	64240800	64240982	10	2	0.093674783
chr1	65532520	65533171	1	4	0.655375925
chr1	65533180	65533557	17	19	0.91897796
chr1	66822426	66822507	0	7	0.053865713
chr1	69520903	69521214	0	10	0.008244476
chr1	76081724	76082024	0	27	5.10E-05
chr1	78224692	78224953	0	1	1
chr1	78443729	78444112	2	1	1
chr1	82268769	82268985	11	50	0.015789123
chr1	82269047	82269297	4	5	1
chr1	84970932	84971156	0	1	1
chr1	85049012	85049201	1	5	0.425626541
chr1	87171169	87171366	1	0	1
chr1	87511041	87511114	2	4	1
chr1	89990896	89991174	1	0	1
chr1	95332076	95332257	0	16	0.001152138
chr1	100818884	100818975	1	1	1
chr1	100828527	100828818	2	1	1
chr1	109947329	109947561	1	0	1
chr1	114311660	114311839	1	18	0.003334058
chr1	114576042	114576392	10	0	0.008244476
chr1	115641946	115642305	1	0	1
chr1	120255293	120255505	1	3	0.655375925
chr1	121485051	121485391	2	1	1
chr1	143743452	143743544	0	1	1
chr1	144941259	144941507	1	17	0.004567361
chr1	147071421	147071622	4	1	0.276758318
chr1	149224321	149224626	1	8	0.079773226
chr1	149911073	149911176	3	0	0.282296651
chr1	150228396	150228564	4	4	0.766225946
chr1	151918135	151918551	0	1	1
chr1	151965048	151965304	4	2	0.515885228
chr1	153505756	153505917	1	0	1
chr1	153581372	153581942	3	2	1
chr1	154927663	154927972	0	7	0.053865713
chr1	154976040	154976512	0	1	1
chr1	155945981	155946195	2	0	0.5215311
chr1	156076024	156076520	2	5	0.723362987
chr1	156426351	156426610	3	2	1
chr1	156643780	156644059	0	7	0.053865713
chr1	156678222	156678290	5	0	0.053865713
chr1	156716847	156717159	7	2	0.183689854
chr1	158969448	158969855	12	14	1

chr1	159158154	159158483	6	8	1
chr1	159835883	159836198	3	0	0.282296651
chr1	160345685	160345985	10	4	0.181027085
chr1	160377715	160378322	1	0	1
chr1	161166954	161167217	7	8	1
chr1	161170780	161171063	3	0	0.282296651
chr1	161359762	161359997	0	3	0.282296651
chr1	161369114	161369631	1	8	0.079773226
chr1	165567506	165567607	1	0	1
chr1	165797702	165797848	0	1	1
chr1	167189272	167189555	1	1	1
chr1	167789520	167789684	2	3	1
chr1	168213156	168213397	0	4	0.157936893
chr1	173992682	173992998	2	1	1
chr1	178695412	178695755	1	0	1
chr1	180991048	180991393	1	1	1
chr1	183009232	183009594	1	5	0.425626541
chr1	185285043	185285517	1	4	0.655375925
chr1	192216372	192216881	7	1	0.079773226
chr1	198126679	198126930	1	2	1
chr1	198905612	198905711	0	1	1
chr1	201472677	201472996	0	8	0.020240968
chr1	202113059	202113130	2	3	1
chr1	205262861	205263089	6	0	0.032658739
chr1	205902815	205902946	4	0	0.157936893
chr1	209571962	209572306	0	1	1
chr1	209943538	209943707	1	0	1
chr1	212732230	212732525	3	3	1
chr1	213088259	213088366	4	0	0.157936893
chr1	214612904	214613134	1	1	1
chr1	224180241	224180461	23	44	0.423785028
chr1	226271210	226271346	0	8	0.032658739
chr1	228652317	228652562	7	8	1
chr1	228783419	228783556	4	8	0.663732448
chr1	234735742	234736193	1	2	1
chr1	234746690	234747464	1	5	0.425626541
chr1	234748376	234748793	0	1	1
chr1	234859028	234859276	0	1	1
chr1	244013490	244013714	4	26	0.019830568
chr1	244013800	244014158	1	21	0.00182029
chr1	244978965	244979134	4	0	0.157936893
chr2	2617165	2617546	0	8	0.032658739
chr2	4561958	4562243	3	2	1
chr2	8818941	8819462	4	4	0.766225946
chr2	9694826	9695338	1	0	1
chr2	25438968	25438984	2	0	0.5215311
chr2	25473046	25473381	3	0	0.282296651
chr2	25473990	25474246	15	1	0.004567361
chr2	26258137	26258469	3	0	0.282296651
chr2	26523579	26523969	0	4	0.157936893
chr2	26980804	26981095	3	1	0.655375925
chr2	29118169	29118450	0	1	1

chr2	29353202	29353443	2	2	1
chr2	32583047	32583203	1	1	1
chr2	38348894	38349260	0	1	1
chr2	39351342	39351699	1	5	0.425626541
chr2	40078945	40079169	0	6	0.091014481
chr2	42328400	42328580	4	0	0.157936893
chr2	43036987	43037375	1	1	1
chr2	43054731	43055003	7	4	0.324634247
chr2	43445685	43446105	2	0	0.5215311
chr2	44395153	44395387	3	4	1
chr2	45878104	45878489	2	33	0.001246943
chr2	46768749	46768995	1	0	1
chr2	47195184	47195354	1	0	1
chr2	48131651	48131798	0	1	1
chr2	54787582	54787734	0	10	0.008244476
chr2	54900772	54901112	1	0	1
chr2	54950513	54950848	0	2	0.5215311
chr2	60782418	60783023	1	0	1
chr2	61764034	61764453	4	1	0.276758318
chr2	61991015	61991045	0	1	1
chr2	61991336	61991654	6	5	0.626627063
chr2	63842292	63842532	4	0	0.157936893
chr2	64872338	64872461	0	2	0.5215311
chr2	64880092	64880113	1	0	1
chr2	64977940	64978539	1	5	0.425626541
chr2	65663429	65663962	4	3	0.766225946
chr2	65804198	65804347	1	0	1
chr2	68478265	68478650	3	1	0.655375925
chr2	68478652	68479043	0	2	0.5215311
chr2	70369635	70370121	1	6	0.276758318
chr2	70528439	70528574	3	0	0.282296651
chr2	74347645	74347970	2	1	1
chr2	74686202	74686541	0	2	0.5215311
chr2	75062006	75062505	6	10	0.850964239
chr2	76804434	76804484	10	0	0.008244476
chr2	85200246	85200385	4	0	0.157936893
chr2	85645552	85645901	1	4	0.655375925
chr2	86114905	86115267	0	1	1
chr2	86790049	86790140	0	1	1
chr2	100721195	100721310	1	9	0.053794069
chr2	101435591	101435686	0	1	1
chr2	104018207	104018535	5	1	0.181107555
chr2	105461383	105461712	0	18	0.00057327
chr2	105469181	105469572	0	8	0.032658739
chr2	121493069	121493479	5	4	0.579206704
chr2	121494291	121494499	4	27	0.016203559
chr2	121624976	121625087	1	46	3.37E-05
chr2	121650749	121650964	4	3	0.766225946
chr2	127783225	127783382	6	8	1
chr2	133104840	133105160	4	10	0.427620295
chr2	145107831	145107988	3	3	1
chr2	145267910	145268206	7	2	0.183689854

chr2	149403630	149403740	1	0	1
chr2	157198929	157199068	2	8	0.258917843
chr2	164204716	164204961	6	5	0.626627063
chr2	172960149	172960531	0	1	1
chr2	178258995	178259123	1	0	1
chr2	178516585	178516875	1	8	0.079773226
chr2	181070510	181070738	0	8	0.020240968
chr2	183371357	183371671	0	15	0.001661934
chr2	191746701	191747181	0	1	1
chr2	192043486	192043824	0	1	1
chr2	192110809	192111325	6	7	1
chr2	200800300	200800360	0	1	1
chr2	201727219	201727543	1	0	1
chr2	201981555	201982080	1	3	0.655375925
chr2	206949873	206950109	20	11	0.209850608
chr2	207872884	207873134	0	5	0.157936893
chr2	208199072	208199522	1	0	1
chr2	208199956	208200108	4	1	0.276758318
chr2	213400865	213401221	0	6	0.091014481
chr2	214053032	214053181	2	4	1
chr2	217500609	217500813	4	8	0.663732448
chr2	218027397	218027528	0	2	0.5215311
chr2	223184143	223184477	4	4	0.766225946
chr2	224590239	224590526	9	1	0.053794069
chr2	228337506	228337706	1	2	1
chr2	232478897	232479242	1	1	1
chr2	232479465	232479901	1	1	1
chr2	232531119	232531362	10	4	0.181027085
chr2	232571720	232572140	6	3	0.258917843
chr2	237081098	237081420	5	0	0.053865713
chr3	5064937	5065367	3	0	0.282296651
chr3	8721554	8721708	0	6	0.091014481
chr3	9774068	9774206	1	0	1
chr3	11683976	11684400	2	5	0.723362987
chr3	13493286	13493422	4	5	1
chr3	14186018	14186254	0	4	0.157936893
chr3	15838088	15838466	0	1	1
chr3	17783415	17783748	2	1	1
chr3	25197162	25197484	3	4	1
chr3	25198320	25198706	0	4	0.157936893
chr3	25790927	25791027	3	1	0.655375925
chr3	37285604	37285823	4	0	0.157936893
chr3	39425710	39425956	0	2	0.5215311
chr3	42067059	42067374	1	12	0.025273489
chr3	42108265	42108417	0	8	0.032658739
chr3	44040595	44040824	1	0	1
chr3	49045273	49045629	4	5	1
chr3	50328943	50329011	3	0	0.282296651
chr3	52311371	52311818	2	8	0.258917843
chr3	52332906	52333176	0	8	0.032658739
chr3	52333459	52333734	0	4	0.157936893
chr3	57741613	57742045	2	3	1

chr3	63897672	63897869	0	4	0.157936893
chr3	64000112	64000448	1	0	1
chr3	69058163	69058360	7	0	0.020240968
chr3	71629255	71629450	1	0	1
chr3	71631724	71631765	0	1	1
chr3	99614871	99614964	1	0	1
chr3	101444504	101444843	1	12	0.025273489
chr3	105086623	105086988	2	3	1
chr3	105087566	105087804	2	0	0.5215311
chr3	112137603	112137867	4	0	0.157936893
chr3	113464079	113464317	2	1	1
chr3	115377789	115377984	10	18	0.608414826
chr3	115503589	115503786	3	1	0.655375925
chr3	119048478	119048663	0	14	0.002427802
chr3	119814513	119814759	2	3	1
chr3	123304965	123305363	5	7	1
chr3	125076098	125076183	4	3	0.766225946
chr3	125709348	125709467	15	46	0.095012377
chr3	128719595	128719792	1	14	0.012384085
chr3	134092482	134092694	6	5	0.626627063
chr3	138553376	138553403	2	0	0.5215311
chr3	140795750	140795979	0	4	0.157936893
chr3	141030581	141030840	2	0	0.5215311
chr3	141596260	141596607	0	5	0.157936893
chr3	149104881	149105054	1	0	1
chr3	149686798	149687294	21	9	0.096627188
chr3	150479320	150479754	1	2	1
chr3	152879008	152879428	0	3	0.282296651
chr3	155464118	155464316	0	5	0.157936893
chr3	155589369	155589752	0	1	1
chr3	156174726	156175037	1	1	1
chr3	156806425	156806708	0	2	0.5215311
chr3	156892642	156893097	4	8	0.663732448
chr3	157053499	157053913	0	7	0.053865713
chr3	159480696	159480930	1	0	1
chr3	170074151	170074450	3	0	0.282296651
chr3	170075984	170076359	9	1	0.053794069
chr3	174159220	174159560	0	1	1
chr3	181444608	181444826	0	9	0.012799435
chr3	181959987	181960273	0	10	0.008244476
chr3	182982882	182983109	0	2	0.5215311
chr3	183354355	183354592	0	1	1
chr3	186490734	186490889	1	2	1
chr3	191645058	191645431	14	0	0.001152138
chr3	193852867	193853278	1	1	1
chr3	193859201	193859364	10	4	0.181027085
chr3	195634639	195635000	0	1	1
chr3	195638765	195638962	1	0	1
chr3	196359040	196359921	1	3	0.655375925
chr4	570075	570469	0	8	0.020240968
chr4	1871836	1872343	1	5	0.425626541
chr4	2537976	2538457	1	3	0.655375925

chr4	5367439	5367695	1	0	1
chr4	6785132	6785508	0	5	0.157936893
chr4	9154755	9155064	21	19	0.639772602
chr4	10110074	10110375	1	0	1
chr4	10457929	10458199	0	5	0.157936893
chr4	24585399	24585457	3	2	1
chr4	25863115	25863459	0	1	1
chr4	38133909	38134252	0	2	0.5215311
chr4	38320554	38320744	0	1	1
chr4	38668646	38669113	1	11	0.036673705
chr4	49497130	49497219	0	7	0.053865713
chr4	56814064	56814428	0	3	0.282296651
chr4	57276482	57276707	2	16	0.044159014
chr4	69242140	69242362	5	0	0.053865713
chr4	69271612	69272009	9	0	0.012799435
chr4	73969950	73970076	4	1	0.276758318
chr4	76650334	76650523	6	8	1
chr4	77380507	77380906	5	8	1
chr4	77507224	77507431	5	8	0.836639738
chr4	77511418	77511611	4	3	0.766225946
chr4	79102525	79102723	39	7	0.001356276
chr4	80979913	80980226	0	1	1
chr4	81000754	81000893	0	3	0.282296651
chr4	89204957	89205089	0	5	0.157936893
chr4	93365408	93365782	49	0	1.56E-07
chr4	98417536	98417833	0	14	0.002427802
chr4	99578768	99578978	3	3	1
chr4	99849403	99849687	3	2	1
chr4	106394527	106394657	1	2	1
chr4	108746737	108746874	1	0	1
chr4	111117502	111117773	7	8	1
chr4	111536700	111537100	7	0	0.020240968
chr4	128543991	128544448	0	10	0.008244476
chr4	129308296	129308446	1	0	1
chr4	138466745	138466966	3	3	1
chr4	140004398	140004661	5	0	0.053865713
chr4	140097511	140097883	1	1	1
chr4	140970501	140970652	0	3	0.282296651
chr4	154179990	154180154	4	2	0.515885228
chr4	160024788	160025397	0	8	0.020240968
chr4	165458099	165458221	0	4	0.157936893
chr4	169514523	169514877	1	0	1
chr4	169553727	169553992	0	16	0.001152138
chr4	174292971	174293266	2	4	1
chr4	176922295	176922469	0	11	0.005401553
chr4	180310567	180310889	0	3	0.282296651
chr4	184365037	184365283	1	1	1
chr4	184909319	184909473	6	3	0.258917843
chr4	185393981	185394319	0	2	0.5215311
chr4	186760269	186760670	9	1	0.053794069
chr5	344315	344494	2	1	1
chr5	399876	400441	0	3	0.282296651

chr5	784822	785022	13	18	0.906368533
chr5	1884189	1884634	0	1	1
chr5	2750161	2750499	10	17	0.608414826
chr5	6765926	6766041	0	1	1
chr5	14264873	14265078	3	2	1
chr5	16713466	16713820	1	8	0.079773226
chr5	18664431	18664441	5	0	0.053865713
chr5	34930349	34930408	0	1	1
chr5	38555160	38555231	2	3	1
chr5	42994875	42995232	0	1	1
chr5	44809928	44810037	2	0	0.5215311
chr5	52786463	52786761	1	8	0.079773226
chr5	57137481	57137841	1	3	0.655375925
chr5	58232888	58233171	21	1	0.000592165
chr5	58423422	58423780	3	9	0.380224116
chr5	66311575	66311912	0	1	1
chr5	67512299	67512629	0	5	0.157936893
chr5	71404044	71404173	3	10	0.294764191
chr5	71659095	71659357	2	2	1
chr5	72792838	72792996	0	4	0.157936893
chr5	76103190	76103429	14	0	0.001152138
chr5	76104913	76105236	1	0	1
chr5	79543040	79543270	4	2	0.515885228
chr5	79553525	79553731	9	1	0.053794069
chr5	81147747	81147896	0	9	0.012799435
chr5	87693343	87693641	0	6	0.091014481
chr5	90677542	90678041	2	24	0.005834359
chr5	93227597	93227648	0	4	0.157936893
chr5	95965654	95965814	0	1	1
chr5	98263728	98264267	7	12	0.739382098
chr5	98274323	98274727	0	36	7.02E-06
chr5	99383421	99383489	1	5	0.425626541
chr5	102091711	102091876	1	4	0.655375925
chr5	107005591	107005952	5	4	0.579206704
chr5	107007996	107008475	3	5	1
chr5	108749269	108749552	0	5	0.157936893
chr5	115909219	115909476	5	3	0.365680796
chr5	115911680	115912085	6	0	0.032658739
chr5	119801290	119801335	0	8	0.032658739
chr5	126113909	126114245	10	6	0.427620295
chr5	126691099	126691332	0	19	0.00057327
chr5	132388526	132388912	0	1	1
chr5	133303540	133303646	4	3	0.766225946
chr5	133863319	133863454	3	4	1
chr5	134260875	134261619	1	2	1
chr5	136482110	136482424	0	9	0.012799435
chr5	137800323	137800628	19	4	0.009412945
chr5	138397695	138397781	1	8	0.119583141
chr5	139935789	139935915	0	1	1
chr5	140893785	140893951	5	4	0.579206704
chr5	147253572	147253800	18	0	0.00029737
chr5	151186903	151187108	1	0	1

chr5	158525070	158525401	0	9	0.012799435
chr5	167126596	167126943	3	0	0.282296651
chr5	172411442	172411563	3	0	0.282296651
chr5	175970446	175970582	0	6	0.091014481
chr5	177543107	177543512	2	3	1
chr6	3231969	3232101	4	10	0.427620295
chr6	4134644	4134699	4	0	0.157936893
chr6	4317355	4317635	1	0	1
chr6	6407633	6407919	0	1	1
chr6	6657680	6658009	0	2	0.5215311
chr6	6687142	6687522	1	0	1
chr6	7141281	7141517	2	0	0.5215311
chr6	7261587	7261718	1	2	1
chr6	7347736	7347857	5	0	0.053865713
chr6	7540852	7541040	3	0	0.282296651
chr6	11203131	11203431	0	5	0.157936893
chr6	12011397	12011810	0	3	0.282296651
chr6	12577736	12577950	1	0	1
chr6	14302630	14302929	4	0	0.157936893
chr6	16759907	16760069	0	1	1
chr6	18023227	18023389	3	3	1
chr6	19804790	19805159	3	4	1
chr6	20404914	20405030	1	1	1
chr6	26030294	26030408	1	0	1
chr6	26126040	26126313	0	1	1
chr6	26195568	26195851	4	5	1
chr6	26198556	26198739	0	1	1
chr6	26205546	26205686	1	5	0.425626541
chr6	26572897	26572943	1	2	1
chr6	27101766	27101832	4	0	0.157936893
chr6	27145959	27146135	2	1	1
chr6	27205426	27205803	1	1	1
chr6	27807335	27807764	0	2	0.5215311
chr6	27862929	27863102	1	2	1
chr6	28457232	28457363	6	9	0.850964239
chr6	28457747	28457874	1	8	0.119583141
chr6	28863360	28863704	1	0	1
chr6	28948717	28949082	1	2	1
chr6	29932758	29932894	4	1	0.276758318
chr6	30292108	30292362	1	0	1
chr6	30650325	30650792	5	0	0.053865713
chr6	30786256	30786485	0	3	0.282296651
chr6	31706444	31706670	1	2	1
chr6	32937568	32937840	2	8	0.183689854
chr6	34992489	34992855	1	0	1
chr6	35457912	35458261	6	2	0.258917843
chr6	36391170	36391315	0	3	0.282296651
chr6	37105519	37105906	12	4	0.07672706
chr6	37109428	37109692	11	0	0.005401553
chr6	37137156	37137361	4	0	0.157936893
chr6	37139955	37140357	1	0	1
chr6	41041411	41041634	5	1	0.181107555

chr6	42409510	42409743	3	3	1
chr6	42750565	42750976	6	15	0.348673561
chr6	42751111	42751510	0	3	0.282296651
chr6	42897799	42897994	0	1	1
chr6	44215588	44215995	13	13	0.895427898
chr6	45391235	45391607	0	1	1
chr6	53224147	53224369	1	4	0.655375925
chr6	53412763	53413115	4	2	0.515885228
chr6	53658190	53658592	1	6	0.276758318
chr6	64283359	64283664	0	2	0.5215311
chr6	74290176	74290567	6	3	0.258917843
chr6	82648143	82648414	1	0	1
chr6	83449905	83450061	0	5	0.157936893
chr6	86113468	86113584	2	1	1
chr6	87861995	87862404	3	0	0.282296651
chr6	90193060	90193227	1	8	0.119583141
chr6	90928191	90928478	2	8	0.258917843
chr6	94126525	94126791	4	3	0.766225946
chr6	94544930	94545284	5	0	0.053865713
chr6	100013022	100013326	0	1	1
chr6	105401504	105401762	30	8	0.013781452
chr6	108278449	108278682	16	22	0.922442218
chr6	109702554	109702734	0	1	1
chr6	112210645	112210790	1	5	0.425626541
chr6	114176188	114176618	0	3	0.282296651
chr6	114179412	114179753	0	8	0.032658739
chr6	114179753	114180491	9	54	0.005059182
chr6	119255722	119255801	1	4	0.655375925
chr6	119417911	119418241	0	7	0.053865713
chr6	121759480	121759718	14	3	0.019387991
chr6	126279142	126279247	0	1	1
chr6	127980869	127981176	2	1	1
chr6	129811704	129811870	1	2	1
chr6	129822488	129822857	1	0	1
chr6	136610278	136610595	1	3	0.655375925
chr6	137106493	137106739	0	1	1
chr6	137539036	137539172	2	0	0.5215311
chr6	138427044	138427737	5	0	0.053865713
chr6	138914413	138914521	9	3	0.130835707
chr6	139348926	139349245	6	0	0.032658739
chr6	143265547	143265862	3	4	1
chr6	143268312	143268448	1	0	1
chr6	147523633	147523841	1	0	1
chr6	149866341	149866483	2	1	1
chr6	151712060	151712284	9	15	0.562262661
chr6	154664945	154665270	4	0	0.157936893
chr6	157342370	157342733	0	3	0.282296651
chr6	159107924	159108086	0	1	1
chr6	159270976	159271290	1	0	1
chr6	159274330	159274843	1	0	1
chr6	160391065	160391180	0	7	0.053865713
chr6	164364796	164365174	0	5	0.157936893

chr6	164365197	164365393	2	8	0.258917843
chr6	164506544	164506871	0	16	0.001152138
chr6	167643939	167644090	14	0	0.001152138
chr6	170054778	170054961	1	1	1
chr7	1576940	1577209	3	4	1
chr7	4681324	4681624	0	1	1
chr7	5464974	5465404	1	7	0.181107555
chr7	5466703	5466913	0	8	0.032658739
chr7	6121125	6121353	0	5	0.157936893
chr7	17453810	17453890	6	1	0.119583141
chr7	17978938	17979247	1	0	1
chr7	20160597	20160639	7	1	0.079773226
chr7	22894393	22894894	2	0	0.5215311
chr7	23507530	23507700	3	0	0.282296651
chr7	23513821	23514332	9	11	1
chr7	27153596	27153915	1	7	0.181107555
chr7	32930202	32930309	0	1	1
chr7	38375146	38375524	2	0	0.5215311
chr7	40756946	40757151	1	12	0.025273489
chr7	43617119	43617318	0	2	0.5215311
chr7	64541567	64541767	7	2	0.183689854
chr7	65958322	65958883	58	116	0.260566631
chr7	72395518	72395970	7	6	0.663732448
chr7	75635203	75635241	0	4	0.157936893
chr7	75795866	75796068	10	8	0.718488007
chr7	75947320	75947579	2	4	1
chr7	75986756	75987413	0	1	1
chr7	75993852	75994107	1	2	1
chr7	79081553	79081716	0	4	0.157936893
chr7	92464580	92464948	1	1	1
chr7	92673632	92673980	0	5	0.157936893
chr7	94003693	94003808	0	6	0.091014481
chr7	94331291	94331435	0	5	0.157936893
chr7	95099932	95099987	0	5	0.157936893
chr7	95950737	95950857	3	1	0.655375925
chr7	97602649	97602932	7	6	0.663732448
chr7	104584095	104584314	2	6	0.515885228
chr7	104585647	104585968	0	1	1
chr7	106808235	106808584	1	2	1
chr7	106809996	106810462	3	8	0.626627063
chr7	110174672	110174898	1	3	0.655375925
chr7	115851518	115851659	1	0	1
chr7	126341159	126341344	4	1	0.276758318
chr7	128898638	128898869	4	8	0.663732448
chr7	129589225	129589755	0	1	1
chr7	129781495	129781566	2	0	0.5215311
chr7	129937447	129937812	0	5	0.157936893
chr7	134405287	134405398	0	8	0.032658739
chr7	137846387	137846527	0	5	0.157936893
chr7	137928274	137928637	4	30	0.00898965
chr7	138917042	138917166	3	0	0.282296651
chr7	140178201	140178305	2	3	1

chr7	140339620	140339692	3	6	1
chr7	140624015	140624151	0	6	0.091014481
chr7	144531962	144532375	0	1	1
chr7	150080908	150081288	1	6	0.276758318
chr7	150811983	150812385	2	2	1
chr7	151156996	151157302	0	3	0.282296651
chr7	152065016	152065252	1	5	0.425626541
chr7	155302115	155302254	0	8	0.032658739
chr7	157190777	157190850	0	1	1
chr7	157477385	157477475	0	8	0.032658739
chr7	157482717	157482925	0	4	0.157936893
chr7	158611053	158611523	1	2	1
chr8	1845042	1845411	0	17	0.000808216
chr8	12611522	12611740	4	1	0.276758318
chr8	19614746	19614834	0	13	0.003595014
chr8	21775760	21776061	1	0	1
chr8	21777927	21778245	1	0	1
chr8	21867756	21867888	1	2	1
chr8	23389968	23390042	1	0	1
chr8	25272283	25272600	1	0	1
chr8	28350766	28351155	0	6	0.091014481
chr8	28353299	28353650	0	13	0.003595014
chr8	28355131	28355408	0	30	2.32E-05
chr8	29595033	29595166	0	3	0.282296651
chr8	29627880	29628174	0	4	0.157936893
chr8	29939759	29940009	0	1	1
chr8	38627887	38628032	1	6	0.276758318
chr8	41997722	41998093	2	4	1
chr8	48424442	48424804	2	1	1
chr8	53853693	53853807	6	2	0.258917843
chr8	54570355	54570746	0	5	0.157936893
chr8	60020455	60020755	0	7	0.053865713
chr8	61851932	61852208	5	3	0.365680796
chr8	63776646	63776831	1	1	1
chr8	66753050	66753226	1	3	0.655375925
chr8	71314592	71315068	0	2	0.5215311
chr8	72582353	72582638	7	0	0.020240968
chr8	75232729	75232884	2	0	0.5215311
chr8	82193224	82193363	1	3	0.655375925
chr8	95704204	95704653	1	0	1
chr8	100024801	100024828	1	0	1
chr8	101426668	101426752	1	0	1
chr8	101880687	101880910	0	1	1
chr8	102138575	102138918	1	1	1
chr8	102167568	102167883	3	21	0.031743678
chr8	103136050	103136227	1	8	0.079773226
chr8	103875108	103875688	7	3	0.183689854
chr8	116679753	116679874	1	0	1
chr8	128909181	128909601	2	0	0.5215311
chr8	131027799	131028139	0	1	1
chr8	141474395	141474593	1	1	1
chr8	141608165	141608486	1	0	1

chr8	143530937	143531089	1	0	1
chr8	144678369	144678736	1	0	1
chr8	144959478	144959538	0	8	0.032658739
chr8	145652910	145653372	0	1	1
chr8	145911157	145911424	6	9	0.850964239
chr9	2015819	2016051	2	3	1
chr9	3498726	3498942	0	7	0.053865713
chr9	3524682	3524950	0	8	0.020240968
chr9	6681240	6681463	1	2	1
chr9	14204497	14204515	3	1	0.655375925
chr9	14322327	14322930	6	30	0.02705372
chr9	27526822	27527188	0	1	1
chr9	27528232	27528576	0	2	0.5215311
chr9	27529087	27529343	0	2	0.5215311
chr9	33165861	33166008	1	0	1
chr9	33818786	33818998	0	2	0.5215311
chr9	34665330	34665683	4	5	1
chr9	35114142	35114458	2	6	0.515885228
chr9	35646805	35647004	1	7	0.181107555
chr9	35731654	35731748	0	1	1
chr9	37464626	37464777	2	1	1
chr9	37472423	37472609	4	7	1
chr9	37885379	37885829	0	1	1
chr9	67340613	67340788	0	8	0.020240968
chr9	71736879	71736919	4	1	0.276758318
chr9	71737082	71737525	2	1	1
chr9	73033942	73034150	3	1	0.655375925
chr9	73034975	73035392	1	0	1
chr9	75142375	75142568	2	0	0.5215311
chr9	76700400	76700644	4	0	0.157936893
chr9	94710992	94711376	9	2	0.130835707
chr9	97302227	97302518	12	0	0.003595014
chr9	100746459	100746666	4	8	0.818932857
chr9	100746978	100747324	3	3	1
chr9	101916985	101917092	0	4	0.157936893
chr9	103065212	103065293	0	1	1
chr9	105967134	105967532	3	0	0.282296651
chr9	107730537	107730916	3	3	1
chr9	108737485	108737661	0	16	0.001152138
chr9	109623229	109623539	0	8	0.020240968
chr9	109624354	109624817	0	7	0.053865713
chr9	109627296	109627685	3	0	0.282296651
chr9	110400003	110400164	3	0	0.282296651
chr9	116385284	116385467	10	0	0.008244476
chr9	121248044	121248306	6	0	0.032658739
chr9	123638652	123638974	0	1	1
chr9	127421434	127421910	1	12	0.025273489
chr9	130856719	130856912	1	3	0.655375925
chr9	132175960	132176363	1	5	0.425626541
chr9	132499986	132500086	5	0	0.053865713
chr9	134248793	134249076	18	23	1
chr9	134268817	134269198	1	6	0.276758318

chr9	135544634	135544971	0	3	0.282296651
chr9	140117847	140118171	7	5	0.489265782
chr10	3509592	3509911	2	7	0.365680796
chr10	3528650	3528886	0	1	1
chr10	4283983	4284338	0	8	0.032658739
chr10	10914100	10914269	0	7	0.053865713
chr10	13800549	13800676	0	6	0.091014481
chr10	17071654	17071768	0	4	0.157936893
chr10	21242029	21242253	4	0	0.157936893
chr10	21389323	21389497	0	4	0.157936893
chr10	21570992	21571356	10	0	0.008244476
chr10	21815961	21816316	6	6	0.818932857
chr10	30073402	30073681	4	0	0.157936893
chr10	32666541	32666796	1	0	1
chr10	33250927	33251178	4	1	0.276758318
chr10	33269500	33269833	0	2	0.5215311
chr10	53798976	53799373	20	0	0.000118909
chr10	61632746	61633129	0	1	1
chr10	61665302	61665611	4	0	0.157936893
chr10	61899497	61899724	27	1	0.000131977
chr10	65024964	65025080	0	2	0.5215311
chr10	71093971	71094473	1	0	1
chr10	76179288	76179303	0	4	0.157936893
chr10	79685316	79685454	2	1	1
chr10	88517239	88517543	3	3	1
chr10	93643662	93644013	4	0	0.157936893
chr10	93647024	93647309	6	14	0.419604142
chr10	94332607	94333009	2	4	1
chr10	94351396	94351581	2	4	1
chr10	94448546	94448797	0	3	0.282296651
chr10	94459498	94459705	0	5	0.157936893
chr10	95241144	95241459	1	0	1
chr10	97026400	97026765	1	3	0.655375925
chr10	104402946	104403165	1	5	0.425626541
chr10	104952218	104952446	0	1	1
chr10	105499238	105499340	1	1	1
chr10	105880732	105881062	0	5	0.157936893
chr10	112260378	112260591	1	0	1
chr10	121276013	121276400	1	0	1
chr10	123681158	123681341	0	8	0.032658739
chr10	126430010	126430088	1	0	1
chr11	1824977	1825015	2	3	1
chr11	3443735	3443965	0	1	1
chr11	4010644	4010827	0	3	0.282296651
chr11	7698422	7698637	2	0	0.5215311
chr11	7894497	7894758	0	5	0.157936893
chr11	8227828	8227969	0	1	1
chr11	9385942	9386178	4	3	0.766225946
chr11	9587450	9588045	2	1	1
chr11	9634851	9634966	1	0	1
chr11	10325286	10325593	4	0	0.157936893
chr11	10953183	10953457	0	4	0.157936893

chr11	12107891	12108203	0	4	0.157936893
chr11	12884765	12884981	4	4	0.766225946
chr11	14541294	14541431	1	3	0.655375925
chr11	16629188	16629719	0	25	6.72E-05
chr11	17229860	17230110	4	3	0.766225946
chr11	19799048	19799380	66	0	1.05E-08
chr11	22850570	22851011	1	4	0.655375925
chr11	35104039	35104441	1	0	1
chr11	36521974	36522224	3	8	0.489265782
chr11	46004524	46004864	17	0	0.00041087
chr11	47573712	47574042	1	0	1
chr11	56978367	56978552	0	5	0.157936893
chr11	57406705	57407185	10	3	0.093674783
chr11	57546007	57546358	7	12	0.739382098
chr11	61581931	61582298	9	7	0.693699509
chr11	62319298	62319562	0	1	1
chr11	62575923	62575928	2	0	0.5215311
chr11	65186495	65186781	1	0	1
chr11	65187497	65187783	3	0	0.282296651
chr11	65244591	65245189	3	6	1
chr11	67140426	67140561	0	3	0.282296651
chr11	70217464	70217641	1	1	1
chr11	71638745	71638991	2	0	0.5215311
chr11	75203346	75203495	2	0	0.5215311
chr11	75273742	75273908	10	6	0.427620295
chr11	95524181	95524553	1	1	1
chr11	95788572	95788913	0	8	0.032658739
chr11	96040237	96040412	0	11	0.005401553
chr11	102962198	102962309	0	3	0.282296651
chr11	110001444	110001788	1	30	0.00016724
chr11	111849144	111849372	1	6	0.276758318
chr11	117777992	117778225	1	0	1
chr11	118490219	118490488	0	6	0.091014481
chr11	118740896	118741079	0	1	1
chr11	118780065	118780161	11	0	0.005401553
chr11	118782966	118783341	11	0	0.005401553
chr11	118795313	118795683	0	3	0.282296651
chr11	118796519	118797248	0	2	0.5215311
chr11	123468959	123469255	9	3	0.130835707
chr11	128204161	128204188	1	15	0.008799071
chr11	128294754	128294909	1	4	0.655375925
chr11	133788177	133788878	24	4	0.002940477
chr12	2905042	2905180	2	3	1
chr12	3309317	3309517	7	4	0.324634247
chr12	4140474	4140706	2	3	1
chr12	6404257	6404585	0	2	0.5215311
chr12	6445838	6446030	3	1	0.655375925
chr12	6450592	6450695	1	0	1
chr12	6642660	6642807	1	1	1
chr12	7034733	7034837	2	2	1
chr12	8179185	8179462	1	8	0.079773226
chr12	10870117	10870324	1	1	1

chr12	12713825	12714272	2	1	1
chr12	12868919	12869312	0	7	0.053865713
chr12	15105315	15105464	0	2	0.5215311
chr12	19283475	19283647	15	9	0.321946112
chr12	20124310	20124596	3	8	0.626627063
chr12	22141031	22141195	0	8	0.032658739
chr12	25539015	25539423	5	5	0.796342034
chr12	26427704	26427972	1	0	1
chr12	28343580	28344183	0	1	1
chr12	29543515	29543857	0	2	0.5215311
chr12	31523882	31524066	12	4	0.07672706
chr12	31902122	31902509	1	1	1
chr12	43460125	43460514	0	10	0.008244476
chr12	45626821	45627051	1	0	1
chr12	46275051	46275183	0	2	0.5215311
chr12	49524470	49524719	4	4	0.766225946
chr12	49581416	49581686	0	3	0.282296651
chr12	49595071	49595208	0	7	0.053865713
chr12	49626770	49626953	0	4	0.157936893
chr12	49628350	49628504	1	7	0.181107555
chr12	52242494	52242842	5	4	0.579206704
chr12	52426736	52427006	16	1	0.003334058
chr12	52890344	52890791	1	0	1
chr12	53339404	53339737	9	0	0.012799435
chr12	53615230	53615593	0	2	0.5215311
chr12	53886746	53887056	0	4	0.157936893
chr12	54752788	54753182	2	8	0.258917843
chr12	57480417	57480583	2	0	0.5215311
chr12	57481596	57482013	0	8	0.032658739
chr12	57487784	57487960	3	4	1
chr12	57852792	57853177	10	0	0.008244476
chr12	66135258	66135456	0	1	1
chr12	66216803	66216958	3	3	1
chr12	66290015	66290373	2	5	0.723362987
chr12	69928213	69928510	0	12	0.005401553
chr12	85304956	85305251	1	3	0.655375925
chr12	90485096	90485339	1	0	1
chr12	94954711	94954943	1	0	1
chr12	95466463	95466955	3	7	0.796342034
chr12	95599476	95599733	3	0	0.282296651
chr12	106975717	106975865	2	12	0.135532242
chr12	106977367	106977421	0	6	0.091014481
chr12	109083701	109083944	0	8	0.032658739
chr12	109232151	109232504	1	0	1
chr12	109896593	109896891	23	6	0.016203559
chr12	110718269	110718488	6	2	0.258917843
chr12	113494488	113494540	3	3	1
chr12	116893427	116893732	0	4	0.157936893
chr12	120851942	120852092	1	3	0.655375925
chr12	122020672	122020887	6	0	0.032658739
chr12	122235096	122235338	3	0	0.282296651
chr12	123205714	123205979	0	1	1

chr12	123753901	123754384	6	34	0.016266363
chr12	125401701	125402105	3	3	1
chr12	125424269	125424636	1	2	1
chr12	133022620	133022875	1	0	1
chr12	133337749	133337914	1	2	1
chr13	22104910	22105317	1	2	1
chr13	31038646	31039124	6	19	0.198864194
chr13	31192407	31192910	0	1	1
chr13	34116443	34116939	11	4	0.135532242
chr13	36992120	36992514	0	2	0.5215311
chr13	41633968	41634674	0	1	1
chr13	42032968	42033273	0	2	0.5215311
chr13	42037572	42037760	0	1	1
chr13	45009239	45009599	2	4	1
chr13	60253581	60253725	0	4	0.157936893
chr13	72438632	72438998	1	1	1
chr13	74709488	74709785	3	3	1
chr13	79979282	79979383	2	0	0.5215311
chr13	89165184	89165239	5	0	0.053865713
chr13	90015415	90015652	4	3	0.766225946
chr13	97877325	97877469	0	2	0.5215311
chr13	98085144	98085529	0	8	0.020240968
chr13	100374414	100374754	1	4	0.655375925
chr13	100632831	100633122	12	70	0.00293397
chr13	103498914	103499292	0	1	1
chr13	106571427	106571722	0	2	0.5215311
chr13	109923586	109923670	1	0	1
chr13	109924354	109924770	4	0	0.157936893
chr13	110789290	110789412	6	0	0.032658739
chr13	113241796	113241876	0	2	0.5215311
chr14	21776916	21777260	29	3	0.000341486
chr14	22007360	22007646	0	7	0.053865713
chr14	23449019	23449266	0	1	1
chr14	23476264	23476564	18	21	1
chr14	35342755	35343037	2	0	0.5215311
chr14	41985046	41985483	6	0	0.032658739
chr14	42074007	42074228	88	1	7.23E-09
chr14	48145260	48145412	0	7	0.053865713
chr14	48729196	48729319	4	0	0.157936893
chr14	50066549	50066872	1	4	0.655375925
chr14	50328500	50328653	10	12	1
chr14	52313595	52314018	0	8	0.020240968
chr14	55140659	55140801	0	4	0.157936893
chr14	55144152	55144371	0	14	0.002427802
chr14	59800569	59800729	0	2	0.5215311
chr14	69618243	69618555	3	3	1
chr14	70309426	70309526	1	1	1
chr14	71375525	71375967	1	0	1
chr14	75725558	75726079	2	19	0.025886364
chr14	77330514	77330625	0	2	0.5215311
chr14	81636723	81636840	9	20	0.300448231
chr14	81685579	81685962	29	24	0.553339429

chr14	85881763	85881928	0	1	1
chr14	89017799	89017937	3	1	0.655375925
chr14	90849508	90849868	0	3	0.282296651
chr14	94425733	94425910	4	0	0.157936893
chr14	96459422	96459739	32	0	5.61E-06
chr14	99439261	99439650	1	2	1
chr14	100046438	100046873	1	0	1
chr14	102414331	102414712	5	8	0.836639738
chr14	105282321	105282580	3	8	0.626627063
chr14	105948317	105948520	1	3	0.655375925
chr15	24007262	24007562	0	8	0.020240968
chr15	25201773	25201958	10	2	0.093674783
chr15	34880647	34880739	5	2	0.365680796
chr15	37387243	37387545	5	0	0.053865713
chr15	38365259	38365671	4	32	0.00615382
chr15	38545850	38546011	1	5	0.425626541
chr15	43415042	43415466	1	0	1
chr15	45076541	45076665	3	1	0.655375925
chr15	52971853	52972053	0	16	0.001152138
chr15	57678191	57678423	7	0	0.020240968
chr15	59161937	59162197	0	1	1
chr15	65596686	65597013	3	3	1
chr15	65714314	65714567	2	5	0.723362987
chr15	66993297	66993561	6	6	0.818932857
chr15	69110522	69110928	1	3	0.655375925
chr15	69111357	69111492	0	2	0.5215311
chr15	69112017	69112260	4	2	0.515885228
chr15	69112518	69112795	2	0	0.5215311
chr15	70391857	70392150	3	3	1
chr15	75162374	75162680	1	0	1
chr15	75491069	75491317	1	3	0.655375925
chr15	85887677	85888013	1	0	1
chr15	86162258	86162663	1	0	1
chr15	86211311	86211623	0	4	0.157936893
chr15	86294716	86295091	3	6	1
chr15	89166426	89166694	2	0	0.5215311
chr15	90577575	90577842	0	1	1
chr15	90625679	90626025	0	1	1
chr15	90944185	90944361	1	6	0.276758318
chr15	91446452	91446660	0	2	0.5215311
chr15	93352331	93352853	5	6	1
chr15	93353892	93354067	2	4	1
chr15	93363577	93363784	5	10	0.693699509
chr15	96883862	96884005	2	4	1
chr15	99396499	99396668	0	3	0.282296651
chr16	3148549	3148694	7	0	0.020240968
chr16	3156536	3156814	2	4	1
chr16	4321878	4321969	1	0	1
chr16	4666160	4666596	1	2	1
chr16	10711030	10711380	1	0	1
chr16	13446558	13446861	6	0	0.032658739
chr16	14727900	14727944	19	1	0.0010241

chr16	21831641	21831891	1	1	1
chr16	26081944	26082240	1	4	0.655375925
chr16	27730096	27730332	2	8	0.183689854
chr16	30411986	30412095	2	8	0.183689854
chr16	47006510	47006928	2	2	1
chr16	49734783	49734889	6	3	0.258917843
chr16	49736384	49736514	3	3	1
chr16	51670878	51671223	2	0	0.5215311
chr16	57770552	57770689	0	2	0.5215311
chr16	68271440	68271789	3	4	1
chr16	68685367	68685537	1	0	1
chr16	68685812	68686213	1	0	1
chr16	69165298	69165486	6	2	0.258917843
chr16	69790578	69790824	3	9	0.380224116
chr16	70409636	70409862	0	3	0.282296651
chr16	70462238	70462586	1	3	0.655375925
chr16	74331368	74331677	5	1	0.181107555
chr16	75589165	75589320	3	3	1
chr16	81572706	81573015	0	1	1
chr16	85588939	85589260	16	9	0.271056473
chr16	89365988	89366342	1	0	1
chr16	89555593	89555744	4	1	0.276758318
chr17	1394300	1394661	5	6	1
chr17	2295885	2296211	0	2	0.5215311
chr17	2302779	2303335	3	4	1
chr17	4811661	4811955	1	5	0.425626541
chr17	4853564	4853842	1	3	0.655375925
chr17	7738786	7739212	7	14	0.534866334
chr17	7745925	7746236	0	1	1
chr17	7790719	7791142	0	4	0.157936893
chr17	8054499	8054792	2	1	1
chr17	8057330	8057971	4	2	0.515885228
chr17	8059825	8060168	1	2	1
chr17	8532655	8532731	1	2	1
chr17	15394031	15394391	1	2	1
chr17	17204554	17204775	3	1	0.655375925
chr17	17655546	17655794	1	2	1
chr17	18127981	18128283	3	6	1
chr17	21179145	21179274	1	3	0.655375925
chr17	29035109	29035511	1	2	1
chr17	29422603	29422824	2	4	1
chr17	30470074	30470477	0	5	0.157936893
chr17	30846376	30846674	0	5	0.157936893
chr17	33775242	33775540	1	2	1
chr17	36607279	36607638	2	1	1
chr17	36608126	36608440	15	8	0.321946112
chr17	36860111	36860394	0	2	0.5215311
chr17	40427584	40427831	0	3	0.282296651
chr17	40672195	40672354	5	5	0.796342034
chr17	41172193	41172347	1	0	1
chr17	42215473	42215959	1	0	1
chr17	42277320	42277798	17	15	0.661899386

chr17	42589148	42589447	14	13	0.798325232
chr17	43660812	43661071	2	5	0.723362987
chr17	43661970	43662245	2	2	1
chr17	43662346	43662645	1	13	0.017601561
chr17	44344188	44344603	3	2	1
chr17	44805020	44805268	0	5	0.157936893
chr17	45609508	45609595	2	0	0.5215311
chr17	46073485	46073859	0	12	0.005401553
chr17	46079921	46080207	3	8	0.489265782
chr17	46080674	46080895	9	5	0.380224116
chr17	46081211	46081467	5	9	0.693699509
chr17	46089518	46089935	1	18	0.003334058
chr17	46091480	46091875	0	6	0.091014481
chr17	46133114	46133483	28	5	0.002982252
chr17	46631559	46631855	1	1	1
chr17	46643674	46643967	0	5	0.157936893
chr17	47072744	47072795	5	2	0.365680796
chr17	47073377	47073972	6	18	0.2398915
chr17	47270241	47270526	2	3	1
chr17	55952019	55952295	1	8	0.119583141
chr17	56031833	56032239	4	5	1
chr17	56064504	56064712	10	5	0.294764191
chr17	57923695	57923901	1	0	1
chr17	60897166	60897493	0	4	0.157936893
chr17	60947604	60947866	0	8	0.020240968
chr17	61229300	61229493	0	23	0.000118909
chr17	62501289	62501664	0	9	0.012799435
chr17	62832792	62832879	0	1	1
chr17	63640120	63640513	2	8	0.258917843
chr17	65714588	65714805	4	0	0.157936893
chr17	66030334	66030546	5	6	1
chr17	71306265	71306421	3	3	1
chr17	73453552	73453640	7	1	0.079773226
chr17	75422524	75422936	2	1	1
chr17	77179430	77179738	26	1	0.000212999
chr17	77752483	77752614	2	8	0.258917843
chr17	78120246	78120260	0	1	1
chr17	78807068	78807189	0	1	1
chr17	78992038	78992243	0	8	0.020240968
chr17	79071186	79071424	4	2	0.515885228
chr17	79370909	79371616	7	8	1
chr17	79481647	79482067	2	7	0.365680796
chr17	80250639	80250877	3	7	0.796342034
chr18	3594945	3595182	9	1	0.053794069
chr18	3624057	3624485	10	1	0.036673705
chr18	3624916	3625098	2	0	0.5215311
chr18	5896017	5896524	0	36	7.02E-06
chr18	7823036	7823398	0	5	0.157936893
chr18	13382409	13382585	0	5	0.157936893
chr18	13500579	13500791	2	7	0.365680796
chr18	24812276	24812565	7	0	0.020240968
chr18	25755012	25755423	2	13	0.101785371

chr18	25755707	25756021	1	7	0.181107555
chr18	29265802	29266023	9	3	0.130835707
chr18	29673345	29673357	1	0	1
chr18	43620394	43620627	12	0	0.003595014
chr18	48722110	48722218	3	2	1
chr18	51796802	51797176	1	1	1
chr18	72783074	72783193	0	1	1
chr18	74205780	74205987	2	2	1
chr18	74277244	74277404	1	3	0.655375925
chr19	1236712	1236817	2	4	1
chr19	1876132	1876305	4	8	0.663732448
chr19	1942083	1942481	2	4	1
chr19	4035839	4036241	0	1	1
chr19	6066779	6067118	0	7	0.053865713
chr19	6108953	6109123	0	3	0.282296651
chr19	6970570	6970736	0	2	0.5215311
chr19	10982844	10983074	3	6	1
chr19	11201017	11201308	2	6	0.515885228
chr19	13048269	13048430	3	0	0.282296651
chr19	13262495	13262885	16	12	0.53285792
chr19	13273763	13274074	6	2	0.258917843
chr19	13734949	13734958	3	1	0.655375925
chr19	13977003	13977139	0	1	1
chr19	14089229	14089592	6	0	0.032658739
chr19	14732908	14733135	9	6	0.534051981
chr19	15442780	15443066	0	2	0.5215311
chr19	16189750	16189956	0	3	0.282296651
chr19	16190179	16190441	1	0	1
chr19	18133047	18133231	5	1	0.181107555
chr19	18414350	18414476	0	4	0.157936893
chr19	19644313	19644713	2	3	1
chr19	34286539	34286949	3	4	1
chr19	34396926	34397258	4	2	0.515885228
chr19	34625324	34625516	2	5	0.723362987
chr19	34850253	34850639	1	2	1
chr19	36135980	36136154	0	9	0.012799435
chr19	36390611	36390765	2	4	1
chr19	36618755	36619084	1	0	1
chr19	36748524	36748632	6	0	0.032658739
chr19	39149280	39149465	1	0	1
chr19	39647074	39647343	1	0	1
chr19	41332354	41332689	5	4	0.579206704
chr19	42757533	42757662	2	2	1
chr19	42787895	42788273	15	36	0.23698892
chr19	44172719	44173156	0	1	1
chr19	45947414	45947761	2	4	1
chr19	45954021	45954410	1	1	1
chr19	45959038	45959239	0	2	0.5215311
chr19	45972449	45972792	3	5	1
chr19	46012616	46012827	1	7	0.181107555
chr19	47289606	47289877	3	0	0.282296651
chr19	47613174	47613340	2	1	1

chr19	47616314	47616715	5	7	1
chr19	48707192	48707637	2	19	0.025886364
chr19	48829288	48829700	3	2	1
chr19	49466927	49467258	4	1	0.276758318
chr19	51198367	51198509	0	7	0.053865713
chr19	52192253	52192670	1	2	1
chr19	52642308	52642717	6	4	0.434449816
chr19	54483562	54483780	2	12	0.135532242
chr19	55852686	55852800	4	0	0.157936893
chr19	55880631	55880993	1	0	1
chr19	56116418	56116798	1	5	0.425626541
chr19	56117497	56117916	1	1	1
chr19	56166911	56167029	3	0	0.282296651
chr19	58378641	58378719	2	0	0.5215311
chr19	58826158	58826684	0	1	1
chr19	58912615	58912937	4	32	0.00615382
chr19	59049715	59049898	1	2	1
chr19	59050121	59050280	3	2	1
chr20	1791861	1792021	2	1	1
chr20	2632310	2632563	1	0	1
chr20	3184352	3184723	1	4	0.655375925
chr20	10652157	10652415	3	0	0.282296651
chr20	17948011	17948391	0	2	0.5215311
chr20	19738542	19738938	4	13	0.272065337
chr20	19973654	19973902	2	0	0.5215311
chr20	20724123	20724398	0	7	0.053865713
chr20	23342283	23342876	12	9	0.534866334
chr20	23603490	23603639	0	1	1
chr20	25229420	25229607	0	2	0.5215311
chr20	31044863	31045002	1	4	0.655375925
chr20	33412283	33412437	3	7	0.796342034
chr20	34329553	34329652	1	1	1
chr20	35464083	35464324	0	2	0.5215311
chr20	40321483	40321696	3	1	0.655375925
chr20	42838603	42838678	3	0	0.282296651
chr20	45312676	45312745	3	1	0.655375925
chr20	45946105	45946373	6	0	0.032658739
chr20	45986646	45986982	0	8	0.032658739
chr20	45987539	45987730	7	6	0.663732448
chr20	45989585	45989758	9	4	0.242318479
chr20	48763559	48763716	0	3	0.282296651
chr20	48782598	48782975	9	4	0.242318479
chr20	49545982	49546212	3	0	0.282296651
chr20	50417061	50417298	20	3	0.002800544
chr20	50418112	50418506	4	6	1
chr20	52210635	52211030	4	8	0.663732448
chr20	52355126	52355268	1	1	1
chr20	52557664	52558019	1	8	0.119583141
chr20	61568729	61568918	13	8	0.348673561
chr20	62490119	62490483	4	2	0.515885228
chr21	15754871	15755060	5	3	0.365680796
chr21	17652375	17652832	0	9	0.012799435

chr21	17678168	17678329	0	7	0.053865713
chr21	17681579	17681973	0	7	0.053865713
chr21	17682177	17682230	0	7	0.053865713
chr21	17682680	17682704	0	8	0.032658739
chr21	32926892	32927153	7	4	0.324634247
chr21	37802414	37802688	2	0	0.5215311
chr21	38122541	38122841	5	8	1
chr21	40420095	40420340	0	1	1
chr21	42741782	42742079	10	2	0.093674783
chr21	46292417	46292604	0	1	1
chr21	46825913	46825966	3	4	1
chr21	46974896	46975226	0	1	1
chr22	17679669	17679817	3	5	1
chr22	18917000	18917375	4	3	0.766225946
chr22	19418329	19418605	2	0	0.5215311
chr22	20142927	20143191	0	25	6.72E-05
chr22	20863743	20864079	1	1	1
chr22	22006192	22006515	10	27	0.176199896
chr22	24255998	24256405	1	5	0.425626541
chr22	35654347	35654528	2	3	1
chr22	36725044	36725849	17	12	0.406279661
chr22	36727225	36728154	3	3	1
chr22	38036218	38036521	4	0	0.157936893
chr22	38294863	38295045	0	1	1
chr22	38900578	38900940	6	2	0.258917843
chr22	41048189	41048279	4	0	0.157936893
chr22	41417958	41418140	3	3	1
chr22	41809914	41810048	1	1	1
chr22	42224532	42224863	0	1	1
chr22	50747064	50747243	0	6	0.091014481
chrM	9254	9630	26	23	0.572880601
chrX	228858	229090	2	1	1
chrX	1512321	1512622	2	2	1
chrX	2846661	2847057	5	2	0.365680796
chrX	2878854	2879108	11	0	0.005401553
chrX	4628732	4629054	0	7	0.053865713
chrX	10547577	10547644	1	0	1
chrX	10628277	10628516	0	6	0.091014481
chrX	10737837	10738234	0	7	0.053865713
chrX	11455772	11455926	0	7	0.053865713
chrX	12968323	12968534	0	1	1
chrX	23902148	23902227	3	0	0.282296651
chrX	27537380	27537609	0	1	1
chrX	29678169	29678340	1	0	1
chrX	34673281	34673506	1	8	0.119583141
chrX	39952070	39952369	12	0	0.003595014
chrX	40704359	40704763	27	1	0.000131977
chrX	40856330	40856698	3	5	1
chrX	40943706	40944104	9	8	1
chrX	41194260	41194445	5	5	0.796342034
chrX	45626790	45627013	0	1	1
chrX	45666477	45666524	0	2	0.5215311

chrX	46184702	46185122	5	0	0.053865713
chrX	46828195	46828358	3	3	1
chrX	47180874	47181032	1	3	0.655375925
chrX	47415549	47415754	4	3	0.766225946
chrX	47467991	47468159	0	6	0.091014481
chrX	48595667	48595795	0	2	0.5215311
chrX	48659269	48659599	0	1	1
chrX	52950030	52950315	4	3	0.766225946
chrX	52964280	52964519	0	8	0.032658739
chrX	53077548	53077992	0	3	0.282296651
chrX	53710746	53711525	4	2	0.515885228
chrX	70401924	70402165	0	5	0.157936893
chrX	73833280	73833412	1	1	1
chrX	82760186	82760390	0	7	0.053865713
chrX	85340326	85340670	1	1	1
chrX	86936727	86936988	5	0	0.053865713
chrX	91034174	91034621	7	11	0.862832683
chrX	93957780	93957839	6	0	0.032658739
chrX	94754800	94754997	0	1	1
chrX	96415016	96415214	0	13	0.003595014
chrX	99672718	99672989	0	6	0.091014481
chrX	106954234	106954564	0	1	1
chrX	122648341	122648651	2	0	0.5215311
chrX	123351390	123351591	1	8	0.079773226
chrX	124338173	124338415	7	9	1
chrX	128225153	128225407	4	3	0.766225946
chrX	133307356	133307594	2	4	1
chrX	133682986	133683438	3	9	0.380224116
chrX	135992459	135992604	2	4	1
chrX	136646161	136646465	15	0	0.000808216
chrX	139015664	139015790	9	7	0.693699509
chrX	139795014	139795245	1	4	0.655375925
chrX	149737629	149737738	1	0	1
chrX	150564710	150565072	0	1	1
chrX	153094447	153094783	4	0	0.157936893
chrX	153193780	153194089	3	12	0.228336139
chrX	153597787	153598195	9	34	0.055760107
